# The Discovery of Acremochlorins O-R from an *Acremonium* sp. through Integrated Genomic and Molecular Networking

**DOI:** 10.3390/jof10050365

**Published:** 2024-05-20

**Authors:** Ge Cui, Luning Zhou, Hanwei Liu, Xuan Qian, Pengfei Yang, Leisha Cui, Pianpian Wang, Dehai Li, Jaclyn M. Winter, Guangwei Wu

**Affiliations:** 1 College of Chemical Engineering, Nanjing Forestry University, Nanjing 210037, China; gecui@njfu.edu.cn (G.C.); xuanqian2002@163.com (X.Q.); 2 Key Laboratory of Marine Drugs, Chinese Ministry of Education, School of Medicine and Pharmacy, Ocean University of China, 5 Yushan Road, Qingdao 266003, China; 18895692529@163.com (L.Z.); dehaili@ouc.edu.cn (D.L.); 3 Ningbo Customs District Technology Center, Ningbo 315100, China; 13646621936@163.com; 4 Ningbo Institute of Marine Medicine, Peking University, Ningbo 315832, China; yangpf0202@163.com (P.Y.); leisha.cui@pkunimm.com (L.C.); wpian@bjmu.edu.cn (P.W.); 5 Department of Pharmacology and Toxicology, College of Pharmacy, University of Utah, Salt Lake City, UT 84112, USA

**Keywords:** *Acremonium*, ascochlorins, antibacterial activity, diasteroisomer, GNPS

## Abstract

The fermentation of a soil-derived fungus *Acremonium* sp. led to the isolation of thirteen ascochlorin congeners through integrated genomic and Global Natural Product Social (GNPS) molecular networking. Among the isolated compounds, we identified two unusual bicyclic types, acremochlorins O (**1**) and P (**2**), as well as two linear types, acremochlorin Q (**3**) and R (**4**). Compounds **1** and **2** contain an unusual benzopyran moiety and are diastereoisomers of each other, the first reported for the ascochlorins. Additionally, we elucidated the structure of **5**, a 4-chloro-5-methylbenzene-1,3-diol with a linear farnesyl side chain, and confirmed the presence of eight known ascochlorin analogs (**6**–**13**). The structures were determined by the detailed interpretation of 1D and 2D NMR spectroscopy, MS, and ECD calculations. Compounds **3** and **9** showed potent antibacterial activity against *Staphylococcus aureus* and *Bacillus cereus*, with MIC values ranging from 2 to 16 μg/mL.

## 1. Introduction

Ascochlorins (ASCs) and their congeners are primarily produced by filamentous fungi and represent a unique class of polyketide–terpenoid hybrid natural products. They are generally characterized by the presence of an orsellinic acid unit combined with a sesquiterpene (C_15_) moiety [1]. They have attracted extensive attention from pharmacologists. Owing to their distinctive structural diversity, they have been reported to exhibit a range of promising biological activities including antitumor [2], anti-inflammatory [3], antimicrobial [4,5], and anti-trypanosome [6,7]. Recent studies indicated that they showed potent hDHODH inhibitory activity, which means they have been involved in the treatment of cancers.

A recent review summarized their structure isolation and identification, biosynthesis, and biological activity in 2023. To date, about 71 ASCs have been reported from filamentous fungi and are classified into three main chemotypes, the linear type, the monocyclic type, and the bicyclic type, which account for about 30%, 65%, and 0.6% of all the ASCs, respectively [8]. In addition, the gene cluster and biosynthesis of the representative products of ASCs, such as ascofuranone and ascochlorin, have been characterized, which are conducive to the discovery of more ASC congeners by genomic mining [1].

The filamentous fungus *Acremonium* sp. was isolated from soil collected on the University of Utah campus, UT, USA, and was shown to produce a rare class of 15-residue peptaibols [9]. In that study, the 38 Mbp genome of the strain was also reported to harbor 44 putative biosynthetic gene clusters, including 1 predicted for ASCs’ biosynthesis. As no ASCs had been previously reported from the target strain, we implemented genomic and GNPS molecular networking to determine if this orphan biosynthetic cluster was indeed responsible for the production of ASCs. Guided by UV absorption and MS data, we identified four undescribed ASCs (**1**–**4**), a newly natural product (**5**), and eight known ASC analogs (**6**–**13**). Herein, details of their isolation, structure elucidation, and antibiotic activities are described.

## 2. Material and Methods

### 2.1. General Experimental Procedures

Optical rotations were measured on an Anton Paar MCP 5500 polarimeter equipped with a sodium lamp (589 nm) and a 25 mm microcell. A Jasco J1500 spectrometer (Jasco Inc., Tokyo, Japan) was used to obtain the electric circular dichroism (ECD) spectra. The 1D and 2D NMR experiments (^1^H, ^13^C, NOESY, COSY, HSQC, and HMBC) were performed at 300 K in CDCl_3_ on a Bruker Avance Neo 600MHz spectrometer (Bruker BioSpin, GmBH) equipped with a Bruker 5 mm PI HR-BBO600s3 Probe. HR-ESIMS was utilized on an LTQ Orbitrap XL mass spectrometer or an Orbitrap Eclipse mass spectrometer (Thermo Fisher Scientific, Waltham, MA, USA) equipped with Xcalibur 4.0 software. The 200–300 mesh silica gel (Shanghai, China), ODS (12 nm, YMC*GEL), and TOYOPEARL HW-40F were employed for column chromatography. HPLC separations were conducted on an Agilent 1260 series pumping system equipped with an Agilent DAD-G7115A refractive index detector on an X-bridge C_18_ column (4.6 × 250 mm, 5 µm, flow rate 1.0 mL/min). RP-HPLC separations were conducted on a Shimadzu LC-20AP series pumping system equipped with a Shimadzu SPD-20A refractive index detector and Shimadzu injector on an X-bridge C_18_ column (10 × 250 mm, 5 µm, flow rate 4.0 mL/min). TLC analyses were carried out using precoated HF254 (0.20 mm thickness) plates (Nuotai, Shanghai, China); compounds were detected by 10% sulfuric acid/ethanol (Sigma-Aldrich, St. Louis, MO, USA). All MS/MS data were converted to mzXML format files by ProteoWizard 3.0 software [10]. Molecular networking was performed using the GNPS data analysis workflow and the spectral clustering algorithm. The spectral networking was imported into Cytoscape (ver. 3.6.1) for visualization.

### 2.2. Fungal Material

The fungal strain *Acremonium* sp. (GenBank accession number MT053262) was originally isolated from soil collected on the University of Utah campus, UT, USA, and formally characterized as an *Acremonium* sp. in a previous publication from our group [9].

### 2.3. Incubation and Extraction

*Acremonium* sp. was cultured on the seed medium Potato Dextrose Agar plates (PDA medium: 20 g of potato extract powder, 20 g of glucose, 18 g of agar in 1 L of tap distilled H_2_O) at 28 °C for four to five days. Subsequently, the large-scale fermentation of *Acremonium* sp. was performed using modified rice solid medium (80 g of rice, 3.0 g/L of NaNO_3_, and 120 mL of H_2_O). Briefly, 120 mL of rice medium was added to a 48 × 1 L Erlenmeyer flask and inoculated using two 5 × 5 mm^2^ agar plugs from the PDA plates. The cultures were incubated at room temperature under static conditions, and after 30 days, they were harvested for chemical analysis. The fermented cultures were extracted three times with equal volumes of EtOAc, and the organic extracts were combined and concentrated under vacuo to provide a crude extract (87.1 g).

### 2.4. Isolation and Purification

Using normal silica gel chromatography, the crude extract was fractionated into nine fractions using different concentrations of petroleum ether, dichloromethane, and methanol. Fr.2 (19.1 g) eluted with 100% dichloromethane was further separated into 7 subfractions (Frs.2-1∼2-7) via ODS silica gel elution using a mixture of H_2_O/MeOH. Fr.2-6 eluted with MeOH/H_2_O (*v*/*v*, 80:20) was separated into twelve subfractions (Frs.2-6-1∼2-6-12) via preparative HPLC (85:15 MeCN–H_2_O with 0.1% formic acid, 4 mL/min, 205 nm and 254 nm) using an ODS column. Fr.2-6-6 was purified by preparative HPLC (60:40 MeCN–H_2_O with 0.1% formic acid, 4 mL/min, 205 nm and 254 nm) to afford **10** (7 mg, *t*_R_ 27 min), **12** (69 mg, *t*_R_ 32 min), **13** (2.2 mg, *t*_R_ 43 min). Fr.2-6-7 was purified by preparative HPLC (65:35 MeCN–H_2_O with 0.1% formic acid, 4 mL/min, 205 nm and 254 nm) to yield **3** (48.9 mg, *t*_R_ 43 min). Fr.2-6-8 was purified by preparative HPLC (70:30 MeCN–H_2_O with 0.1% formic acid, 4 mL/min, 205 nm and 254 nm) to afford **11** (27.3 mg, *t*_R_ 27 min) and **4** (1.1 mg, *t*_R_ 38 min). Fr.2-7 was further separated into seven subfractions (Frs.2-7-1∼2-7-7) via HW-40F silica gel elution with CH_2_Cl_2_/MeOH (*v*/*v*, 1:1) according to HPLC profiles. Among them, Fr.2-7-6 was purified by preparative HPLC (70–100% MeCN–H_2_O with 0.1% formic acid, 4 mL/min, 205 nm and 254 nm) to yield **9** (2.9 mg, *t*_R_ 60 min), **1** (1.3 mg, *t*_R_ 41 min), **2** (1.2 mg, *t*_R_ 42 min), **5** (4.2 mg, *t*_R_ 50 min), **6** (4.2 mg, *t*_R_ 51.7 min), **7** (2.1 mg, *t*_R_ 53.5 min), **8** (2.1 mg, *t*_R_ 58.5 min).

Acremochlorin O (**1**)*:* yellow oil; [*α*]^25^_D_ + 8 (*c* 0.1, MeOH); UV (MeOH) *λ*_max_ (log *ε*) 270 (4.06), 322 (1.09), 198 (2.33); ECD (0.15 mg/mL, MeOH) *λ*_max_ (Δ *ε*) 207 (+14.40), 232 (−8.99), 269 (+0.88), 318 (−7.15); ^1^H and ^13^C NMR data, Table 1, Figures S6–S17; HR-ESIMS *m*/*z* 405.1840/407.1804 (3:1) ([M+H]^+^/[M+2+H]^+^, calcd for C_23_H_30_ClO_4_, 405.1827/407.1798).

Acremochlorin P (**2**): yellow oil; [*α*]^25^_D_—35.6 (*c* 0.18, MeOH); UV (MeOH) *λ*_max_ (log *ε*) 270 (4.81), 322 (1.99), 200 (2.82); ECD (0.15 mg/mL, MeOH) *λ*_max_ (Δ *ε*) 204 (−20.44), 232 (+3.41), 272 (−10.58), 322 (+6.25); ^1^H and ^13^C NMR data, Table 1, Figures S18–S29; HR-ESIMS *m*/*z* 405.1843/407.1807 (3:1) ([M+H]^+^/[M+2+H]^+^, calcd for C_23_H_30_ClO_4_, 405.1827/407.1798).

Acremochlorin Q (**3**): brown amorphous powder; [*α*]^25^_D_—42.8 (*c* 0.29, CH_2_CL_2_); UV (MeOH) *λ*_max_ (log *ε*) 270 (4.06), 322 (1.09), 198 (2.33); ECD (0.15 mg/mL, MeOH) *λ*_max_ (Δ *ε*) 205 (+9.01), 305 (−5.25); UV (MeOH) *λ*_max_ 298 (3.52), 240 (3.18), 338 (1.43); ^1^H and ^13^C NMR data, Table 1, Figures S30–S37; HR-ESIMS *m*/*z* 421.1775/423.1741 (3:1) ([M-H_2_O+H]^+^/[M+2-H_2_O+H]^+^, calcd for C_23_H_30_ClO_5_, 421.1776/423.1747).

Acremochlorin R (**4**): yellow oil; [*α*]^25^_D_—3.6 (*c* 0.1, CH_2_CL_2_); UV (MeOH) *λ*_max_ (log *ε*) 294 (4.06), 336 (3.87), 232 (4.16); ^1^H and ^13^C NMR data, Table 1; HR-ESIMS *m*/*z* 465.2039/467.2012 (3:1) ([M-H_2_O+H]^+^/[M+2-H_2_O+H]^+^, calcd for C_25_H_34_ClO_6_, 465.2038/467.2009).

4-chloro-5-methyl-2-((2*E*,6*E*)-3,7,11-trimethyldodeca-2,6,10-trien-1-yl)benzene-1,3-diol (**5**): brown oil; UV (MeOH) *λ*_max_ (log *ε*) 198 (1.22), 282 (2.43), 342 (1.70); ^1^H and ^13^C NMR data, Table 1, Figures S46–S51; HR-ESIMS *m*/*z* 363.2085/365.2055 (3:1) ([M+H]^+^/[M+2+H]^+^, calcd for C_22_H_32_ClO_2_, 363.2085/365.2056).

### 2.5. Computation Section

Conformational searches were carried out using Spartan’14 (Wavefunction Inc., Irvine CA USA), based on the MMFF94. All conformers were optimized with DFT calculations at the B3LYP/6-31+G(d) level using the Gaussian 09 program [11,12]. For ECD calculations, TDDFT calculations were performed on the two lowest-energy conformations for **1** and **2** (>5% population) at the B3LYP/6-31+G(d) levels. In addition, the four lowest-energy conformations for **3** (>5% population) were calculated.

### 2.6. Antimicrobial Activities

The minimum inhibitory concentrations (MICs) were determined in 96-well plates using the microdilution method to screen compounds **1**–**9** and **11** for bioactivity. To prepare the inoculum for susceptibility testing, bacteria were streaked independently onto LB agar plates and incubated overnight at 37 °C. Individual colonies were then isolated and transferred to 50 mL of LB liquid medium and incubated at 37 °C for 4–6 h. The culture density was adjusted with LB liquid medium so that a concentration of 5 × 10^6^ cfu/mL was achieved. Compounds **1**–**9** and **11** were tested for their individual activity against *S. aureus*, MRSA, MRCNS, *B. Subtilis*, and *B. cereus* using chloramphenicol as a positive control (64 μg/mL dissolved in DMSO). Briefly, **1**–**9** and **11** were dissolved in DMSO to generate 128 mg/mL stock solutions. The stock solutions were then serially diluted with LB liquid medium to afford working concentrations of 128 to 2 μg/mL. More specifically, to a 96-well microtiter plate, 2 μL stock solutions mixed with 98 μL of LB liquid medium was added to well A1. From this mixture, 50 μL was transferred to well A2 and mixed with 50 μL of fresh LB media. This process was repeated across the 96-well plate, and 50 μL of the appropriate bacterial cultures was then added to each well. The plates were incubated at 37 °C for 16–20 h. MIC values were determined by visual inspection and verified with an OD_600_ measurement using a BioTek Neo2 plate reader (Agilent, Winooski, VT, USA). The respective MIC values for **1**–**9** and **11** are reported in Table 2. All the pathogenic strains were clinical isolates and donated by the Marine Medicinal Biological Resources Center, Ocean University of China. Specific strain information can be found in Table S2.

### 2.7. Hydroxyl Radical Scavenging Activity

The Fenton reaction was used to produce hydroxyl radicals, which reacted with salicylic acid to form 2,3-dihydroxybenzoic acid with special absorption at 510 nm.

The test compound was prepared into a 200 mM solution with DMSO as the solvent. Then, 25 μL of the 200 mM sample solution, 25 μL of 9 mM FeSO_4_·7H_2_O, 25 μL of 9 mM salicylic acid, and 25 μL of 8.8 mM H_2_O_2_ were added into the 96-well plate successively and mixed well. After heating in a 37 °C water bath for 30 min, it was taken out and its absorbance at 510 nm was measured using a BioTek Neo2 plate reader.

## 3. Results and Discussion

**Figure 1:**
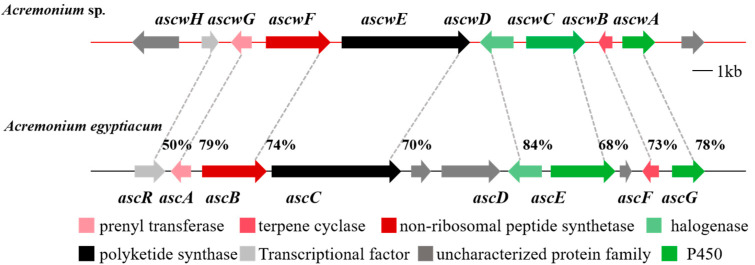
Genomic analysis of *Acremonium* sp. Organization of the ASCs’ biosynthetic gene cluster identified in *Acremonium* sp. (*ascw*) (GenBank Accession number PP795974) in comparison to the ASC gene cluster from *A. egyptiacum* (*asc-1*) (GenBank Accession number LC406756).

The 38 Mbp genome of *Acremonium* sp. was previously sequenced and assembled, and the antiSMASH analysis revealed that a gene cluster, hereby named *ascw*, showed high similarity (87%) at the amino acid level to the characterized *asc-1* gene cluster (Figure 1 and Figure S1). A more detailed bioinformatic analysis of the *ascw* gene cluster revealed that all eight of the genes encoding enzymatic machinery responsible for ASCs’ assembly in *A. egyptiacum* were present in *ascw*, suggesting the ability of *Acremonium* sp. to produce ASCs [1].

**Figure 2:**
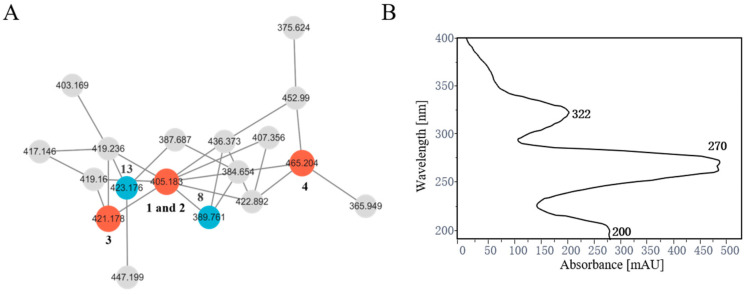
Ascochlorin (ASC) derivatives observed. (**A**) GNPS molecular networking highlighting the cluster associated with ASCs. Acremochlorins O–R (**1**–**4**) are shown as red nodes, known analogs ilicicolin A (**8**) and ascofuranone (**13**) are shown as blue nodes, and unknown compounds are shown in gray. (**B**) UV profile of ASCs.

To evaluate whether the strain significantly produced ASCs or not, the fungal strain was cultured in rice media (80 g of rice, 3.0 g/L of NaNO_3_, and 120 mL of H_2_O) for 30 days. The EtOAc extract was evaluated by LC-MS/MS in the positive mode, and the data were processed through GNPS (http://gnps.ucsd.edu, accessed on 22 December 2023). The obtained molecular networking featured 13 clusters and 91 nodes, with GNPS analysis uncovering a cluster of 23 nodes matching ASC compounds, which displayed typical isotopic peaks for monochloride compounds in the grouped structure (Figure 2, Figure S2 and Figure S3). Known compounds **8** and **13** were directly identified by molecular networking. Based on the UV absorption of known compounds, further targeted isolation resulted in a total of 13 Ascochlorin (ASC) derivatives (Figure 2). Four compounds were new, including **1** and **2** (*m*/*z*: 405. 1840 and 405.1843), **3** (*m*/*z*: 421. 1775), and **4** (*m*/*z*: 465. 2039).

Compound **1** was obtained as a yellow oil. The HR-ESIMS exhibited a characteristic pseudomolecular ion at *m*/*z* 405.1840/407.1804 in a ratio of 3:1 ([M+H]^+^/[M+2+H]^+^, calcd for C_23_H_30_ClO_4_, 405.1827/407.1798), suggesting the presence of a chlorine atom in **1** and supporting the molecular formula of C_23_H_31_ClO_5_ containing nine degrees of unsaturation. Further analysis of the 1D NMR and HSQC data showed the presence of a hexasubstituted benzene moiety (*δ*_C_ 113.6, 158.9, 107.9, 156.3, 116.2, and 140.8), a non-conjugated ketone carbon (*δ*_C_ 213.8), one aldehyde carbon (*δ*_C/H_ 193.5/10.13), a double bond (*δ*_C/H_ 116.2/6.73 and 126.5/5.53), five methyls, four sp^3^ methylenes, two sp^3^ methines, and two quaternary carbons (one oxygenated carbon *δ*_C_ 82.0 and one sp^3^ *δ*_C_ 43.3), indicating the existence of two additional ring systems in the structure of **1** (Table 1).

**Figure 3:**
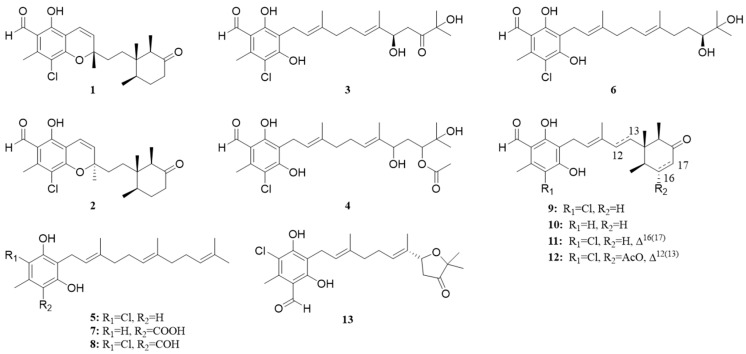
Structures of compounds **1**–**13** isolated from *Acremonium* sp.

**Figure 4:**
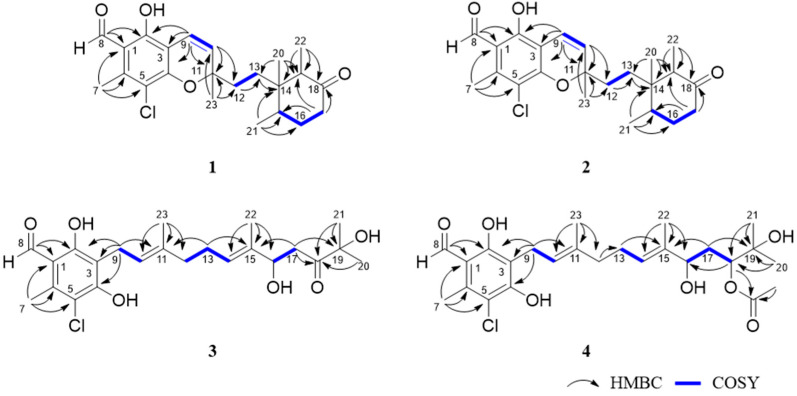
Key HMBC and COSY correlations of compounds **1**–**4**.

The comparison of NMR data between **1** and co-isolated ilicicolin C (**9**) revealed that both compounds share identical monochlorinated benzaldehyde and cyclohexanone moieties (Figure 3). In **1**, the double bond is located between C-9 and C-10, whereas it is in position between C-10 and C-11 in **9**. This was confirmed by the ^1^H-^1^H COSY correlations of H-9/H-10. In addition, an oxygenated quaternary carbon was confirmed at C-11 (*δ*_C_ 82.0), supported by the HMBC correlations from Me-23 (*δ*_C/H_ 27.6/1.49) to C-10 (*δ*_C_ 126.5), C-11 and C-12 (*δ*_C_ 34.6), and H-9 (*δ*_C/H_ 116.2/6.73) to C-10, and C-11 (Figure 4). Subsequently, the HMBC correlations of H-9 with C-2 (*δ*_C_ 158.9), C-3 (*δ*_C_ 107.9), C-4 (*δ*_C_ 156.3), and Me-20 (*δ*_C/H_ 15.6/0.58) with C-13 (*δ*_C_ 30.8) allowed us to establish the connections of the monochlorinated benzaldehyde and cyclohexanone group by a single bond between C-3 and C-9. To satisfy the degree of unsaturation, the molecular formula and downfield chemical shift of C-11 (*δ*_C_ 82.0), a benzopyran moiety, was proposed, thus assigning the planar structure of **1**.

Compound **2** was obtained as yellow oil and was determined by HR-ESIMS data to be at 405.1843/407.1807 (3:1) ([M+H]^+^/[M+2+H]^+^, calcd for C_23_H_30_ClO_4_, 405.1827/407.1798) and to have the same molecular formula of C_23_H_29_ClO_4_ as **1**. When isolated using HPLC, compounds **1** and **2** eluted as adjacent peaks (Figure S4). The detailed inspection of 1D and 2D NMR data revealed that compounds **1** and **2** share identical planar structures. Slight differences in chemical shifts, primarily within the cyclohexanone moiety between **1** and **2,** were observed, including Me-21 (*δ*_C/H_ 15.1/0.85 for **1** vs. 15.0/0.90 for **2**), Me-22 (*δ*_C/H_ 7.5/0.93 for **1** vs. 7.6/0.88 for **2**), H-19 (*δ*_C/H_ 50.6/2.44 for **1** vs. 50.6/2.40 for **2**), H-15 (*δ*_C/H_ 36.3/1.94 for **1** vs. 36.4/1.97 for **2**), and H-12 (*δ*_C/H_ 34.6/1.70 for **1** vs. 34.6/1.86 for **2**) (Figure S5). Thus, **1** and **2** are isomers of each other.

**Figure 5:**
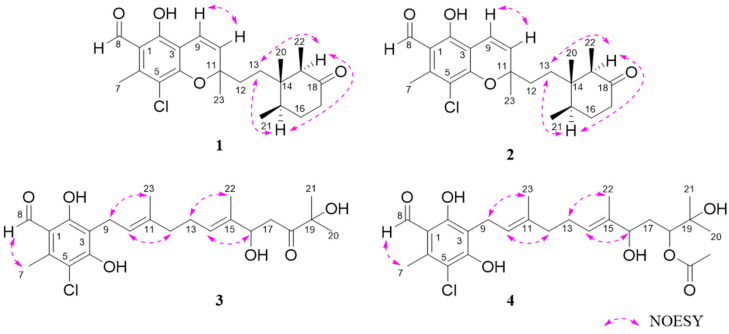
Key NOESY correlations of compounds **1**–**4**.

The relative configurations of **1** and **2** were assigned by key NOESY correlations and coupling constants (Table 1 and Figure 5). The signal intensity of H-15 and H-13 (*δ*_C/H_ 30.8/1.61 for **1** and 30.6/1.57 for **2**) increased after the irradiation of H-19, indicating a similar relative configuration of the cyclohexanone moiety in **1** and **2**. The *Z* configuration of the *Δ*^9(10)^ double bond of **1** and **2** was deduced through a strong NOESY correlation between H-9 and H-10 and relatively small coupling constants (*J*_H-9/H-10_ = 10.1 Hz). The relative configuration of C-11 was not deduced.

**Figure 6:**
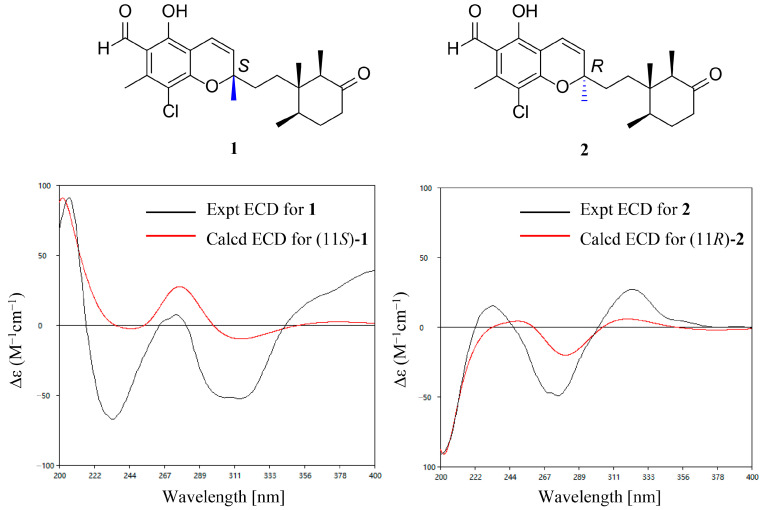
Experimental and calculated ECD spectra for **1** and **2**.

The ECD calculation and biosynthetic origin were involved in the assignment of the absolute configuration of **1** and **2**. Surprisingly, the experimental ECD curves of **1** and **2** showed almost opposite cotton effects (Figure 6). We propose that the observed differences in the ECD cotton effects are primarily due to the benzopyran moiety, rather than the cyclohexanone group, as shown in a previous study, and the ECD method is suitable for the assignment of C-11 [13]. Thus, theoretical ECD calculations were performed using the time-dependent density functional theory (TD-DFT) approach. As shown in Figure 6, the experimental ECD spectrum of **1** displayed a good match with the calculated spectrum of 11*S*, while the curve of **2** was in accord with that of the 11*R*. The 14*S*, 15*R*, and 19*R* of **1** and **2** were deduced on the basis of the enzyme-mediated formation of the cyclohexanone group [1,2] and the same relative configuration of this moiety to that of co-isolated compound **9**. In fact, the literature survey revealed that all the cyclohexanone groups in ASCs share ommon stereochemistry without exception [8]. Thus, the absolute configurations were finally determined to be 9*Z*, 11*S*, 14*S*, 15*R*, 19*R*-**1** and 9*Z*, 11*R*, 14*S*, 15*R*, 19*R*-**2**, respectively, indicating that compounds **1** and **2** are diastereoisomers.

**Figure 7:**
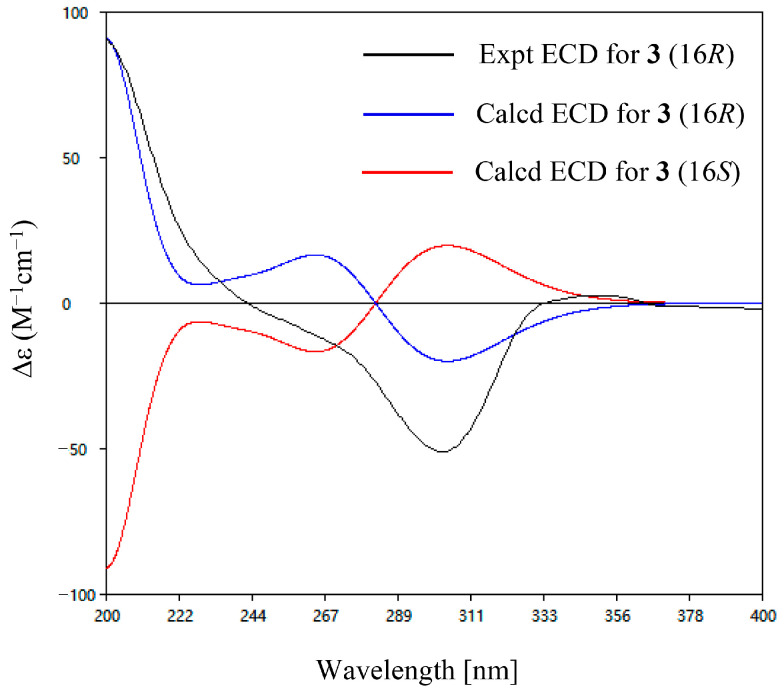
Experimental and calculated ECD spectra of compound **3**.

Compound **3** was obtained as a brown, amorphous powder. The molecular formula C_23_H_31_ClO_6_ was established by HR-ESIMS at *m*/*z* 421.1775/423.1741 ([M-H_2_O+H]^+^/[M+2-H_2_O+H]^+^, calcd for C_23_H_30_ClO_5_, 421.1776/423.1747). The NMR data of **3** were highly similar to those of the co-isolated chlorocylindrocarpol (**6**), suggesting that **3** contained an acyclic sesquiterpene moiety (Figure 3) [14]. The only difference between **3** and **6** is that the double bond group of C-16 and C-18 was substituted by a hydroxyl group and a non-conjugated ketone moiety in **3**. The differences were supported by the COSY correlations of H-16/H-17 and HMBC correlations of Me-20 (*δ*_C/H_ 24.4/1.28) with C-18 (*δ*_C_ 218.1) and Me-22 (*δ*_C/H_ 11.3/1.63) with C-14 (*δ*_C/H_ 128.7/5.5), C-15 (*δ*_C_ 133.1), C-16 (*δ*_C/H_ 77.9/4.52) (Figure 4). The *E* configuration of both *Δ*^10^ and *Δ*^14^ double bonds was assigned by NOESY correlations between H_2_-9 and Me-23, H-10 and H_2_-12, H_2_-13 and Me-22, and H-14 and H-16, respectively (Figure 5). The ECD calculation was used to address the absolute configuration of C-16; the calculated ECD curve of **3** showed positive Cotton effects at around 200–250 nm and negative Cotton effects at around 280–320 nm, coinciding well with the experimental ECD spectrum and suggesting a 16*R*-configuration in **3** (Figure 7).

Compound **4** was obtained as a yellow oil. Analysis of the HR-ESIMS data showed a characteristic pseudomolecular ion indicative of a monochloroinated compound at *m*/*z* 465.2039/467.2012 in a ratio of 3:1 ([M-H_2_O+H]^+^/[M+2-H_2_O+H]^+^) and gave the molecular formula of C_25_H_35_ClO_7_. The high similarity of NMR spectroscopic data of **4** to compound **3** suggested that both structures were closely related. The difference between compound **3** and **4** was the *O*-acetylation of the ketone moiety at C-18 in **3**, which was confirmed by COSY correlations between H-16, H-17 and H-18, and the key HMBC between Me-21 (*δ*_C/H_ 25.5/1.23) and H-18 (*δ*_C/H_ 79.6/4.99). Additionally, the acetyl group was determined to be attached to OH-18 through HMBC correlations between H-1′ (*δ*_C/H_ 21.2/2.07) and H-18 with C-2′ (*δ*_C_ 170.8). Both *Δ*^10^ and *Δ*^14^ double bonds were also assigned as *E* configuration by NOESY correlations (Figure 5). Attempts to obtain crystals for further analysis were unsuccessful.

Compound **5** was isolated as a newly natural product, which was initially reported as a chemically synthesized product, named 4-chloro-5-methyl-2-((2*E*,6*E*)-3,7,11-trimethyldodeca-2,6,10-trien-1-yl)benzene-1,3-diol (**5**). The structure of **5** was determined by comparing NMR data [15]. In addition to the five new structures, eight known ascochlorin derivatives, chlorocylindrocarpol (**6**) [14], grifolic acid (**7**) [16], ilicicolin A (**8**) [17], ilicicolin C (**9**) [18], LL-Z 1272e (**10**) [19], cylindrochlorin (**11**) [20], ilicicolin F (**12**) [19], and ascofuranone (**13**) [21], were identified by comparison with published NMR data.

Compounds **1**–**9** and **11** were assayed for their antimicrobial activities against Gram-positive pathogenic bacteria *Staphylococcus aureus* ATCC29213, methicillin-resistant *Staphylococcus aureus* (MRSA), methicillin-resistant coagulase-negative *staphylococci* (MRCNS), and *Bacillus cereus*, as well as the plant pathogenic fungi *Botrytis cinerea*, *Fusarium graminearum*, *Colletotrichum*, *Fusarium oxysporum*, and *Exobasidium vexans*. In summary, all compounds lacked inhibitory effects against the plant pathogenic fungi, whereas compound **3** exhibited potent inhibitory antibacterial effects with MIC values ranging from 2 to 8 μg/mL (Table 2), which exceeded the positive control, chloramphenicol [22,23]. The preliminary analysis of the structure–activity relationship revealed that the ketone moiety, rather than the acetoxyl group at C-18, is helpful to improve the antibacterial activity. In addition, compounds **1**–**5** also underwent testing for antioxidant activity. They displayed moderate antioxidant properties with hydroxyl radical clearance rates of 64.14%, 65.77%, 67.04%, 68.61%, and 69.32%, respectively, while the positive control, vitamin C, exhibited an 81.69% hydroxyl radical clearance rate at a concentration of 50 μM.

## 4. Conclusions

In summary, using integrated genomic and GNPS molecular networking, four undescribed ASC congeners, a newly natural product, and seven known ones were discovered from the soil-derived fungus *Acremonium* sp. Particularly, Acremochlorin O (**1**) and Acremochlorin P (**2**) possessed an unusual benzopyran moiety and were diastereoisomers of each other that had not been discovered in ASCs to date. Our finding indicated that ASCs have promising potential as lead compounds for developing new antibacterial agents.

## Figures and Tables

**Table 1:** The ^1^H NMR and ^13^C NMR data of compounds **1**–**4** in CDCl_3_.

Compound	1		2		3		4	
Position	*δ*_C_	*δ*_H_ (*J* in Hz)	*δ*_C_	*δ*_H_ (*J* in Hz)	*δ*_C_	*δ*_H_ (*J* in Hz)	*δ*_C_	*δ*_H_ (*J* in Hz)
**1**	113.6		113.6		113.7		113.7	
**2**	158.9		158.9		162.3		162.3	
**3**	107.9		107.9		114.5		114.5	
**4**	156.3		156.3		156.5		156.6	
**5**	116.2		116.2		113.3		113.4	
**6**	140.8		140.8		137.8		137.8	
**7**	14.7	2.60, s	14.7	2.60, s	14.6	2.61, s	14.6	
**8**	193.5	10.13, s	193.5	10.13, s	193.5	10.14, s	193.4	10.14, s
**9**	116.2	6.73, d (10.1)	116.2	6.73, d (10.1)	22.1	3.39, d (7.1)	22.1	3.39, d (7.1)
**10**	126.5	5.53, d (10.1)	126.6	5.53, d (10.1)	121.4	5.21, t (7.3)	121.2	5.20, t (7.2)
**11**	82.0		82.0		136.1		136.5	
**12**	34.6	1.70, m overlapping	34.6	1.86, m overlapping	39.1	2.04, m	39.2	2.01, m
**13**	30.8	1.61, 1.44, m overlapping	30.8	1.57 1.44, m overlapping	26.1	2.16, m	26.2	2.11, m
**14**	43.3		43.3		128.7	5.50, t (7.2)	127.5	5.41, t (7.0)
**15**	36.3	1.94, m	36.4	1.97, m	133.1		134.3	
**16**	31.0	1.84, 1.62, m overlapping	31.0	1.84, 1.63, m overlapping	77.9	4.52, m	80.9	4.31, t (7.7)
**17**	41.7	2.33, m	41.7	2.33, m	40.1	2.38, m	37.1	1.73, 2.44, m
**18**	213.8		213.7		218.1		79.6	4.99, dd (4.4, 7.0)
**19**	50.6	2.44, q (6.7)	50.6	2.40, q (6.8)	80.9		81.9	
**20**	15.6	0.58, s	15.6	0.58, s	24.4	1.28, s	22.8	1.22, s
**21**	15.1	0.85, d (6.8)	15.0	0.90, d (7.0)	22.1	1.22, s	25.5	1.23, s
**22**	7.5	0.93, d (6.7)	7.6	0.88, d (7.1)	11.3	1.63, s	11.1	1.59, s
**23**	27.6	1.49, s	27.5	1.49, s	16.3	1.79, s	16.3	1.77, s
**1′-OAc**							21.2	2.07, s
**2′**							170.8	
**2-OH**		12.71, s		12.70, s		12.69, s		12.69, s
**4-OH**						6.43, s		6.48, s

**Table 2:** Antimicrobial activities of compounds **1**–**9** and **11** (MIC, µg/mL).

	Strain	*S. aureus* ATCC29213	*S. aureus* MRSA	*S. aureus* MRCNS	*B. cereus*
Compounds					
1		>128	>128	>128	>128
2		>128	>128	>128	>128
3		4	8	2	4
4		32	64	32	32
5		>128	>128	>128	16
6		32	32	32	16
7		64	64	32	16
8		>128	>128	>128	>128
9		4	16	4	16
11		>128	64	32	32
DMSO		>128	>128	>128	>128
chloramphenicol		8	8	4	4

All assays were performed in triplicate.

## Data Availability

Data are contained within the article and Supplementary Materials.

## Supplementary Materials

The following supporting information can be downloaded at https://www.mdpi.com/article/10.3390/jof10050365/s1: The GNPS, NMR, HRESIMS, IR, and ECD data for **1**–**5** are included.
