# Supplementary material for: The Discovery of Acremochlorins O-R from an Acremonium sp. through Integrated Genomic and Molecular Networking
Source: J Fungi (Basel). 2024 May 20;10(5):365. doi: 10.3390/jof10050365 (PMC11122259; doi:10.3390/jof10050365)
Supplement: Supplementary file 1 [file jof-10-00365-s001.zip › jof-2969475-supplementary.pdf]

Supporting Information

The Discovery of Acremochlorins O-R from an *Acremonium* sp. through Integrated Genomic and Molecular Networking

Ge Cui [1,†], Luning Zhou [2,†], Hanwei Liu [3], Xuan Qian [1], Pengfei Yang [4], Leisha Cui [4], Pianpian Wang [4], Dehai Li [2], Jaclyn M. Winter [5,*] and Guangwei Wu [1,*]

[1] *College of Chemical Engineering, Nanjing Forestry University, Nanjing 210037, China*

[2] *Key Laboratory of Marine Drugs, Chinese Ministry of Education, School of Medicine and Pharmacy, Ocean University of China, 5 Yushan Road, Qingdao 266003, China*

[3] *Ningbo Customs District Technology Center, Ningbo 315100, China*

[4] *Ningbo Institute of Marine Medicine, Peking University, Ningbo 315832, China*

[5] *Department of Pharmacology and Toxicology, College of Pharmacy, University of Utah, Salt Lake City, UT 84112, USA*

*Corresponding authors

E-mail addresses: gweiwu@njfu.edu.cn (Guangwei Wu); jaclyn.winter@utah.edu (Jaclyn M. Winter);

List of Supporting Information

1. **Figure S1.** Identification of the *ascw* gene cluster in *Acremonium* sp. 5

   Figure S2. GNPS Molecular Networking analysis of *Acremonium* sp. 6

   Figure S3. GNPS Molecular Networking of ASCs in *Acremonium* sp. 6

   Figure S4. HPLC identification of compounds **1** and **2**. 7

   Figure S5. Comparison of ^{1}H NMR spectra of compounds **1** and **2** in $CDCl_3$. 7

2. **Figure S6.** ^{1}H NMR (600 MHz) spectrum of compound **1** in $CDCl_3$. 8

   Figure S7. ^{13}C NMR (600 MHz) spectrum of compound **1** in $CDCl_3$. 9

   Figure S8. HSQC NMR (600 MHz) spectrum of compound **1** in $CDCl_3$..................... 10

   Figure S9. HMBC NMR (600 MHz) spectrum of compound **1** in $CDCl_3$................... 11

   Figure S10. ^{1}H-^{1}H COSY NMR (600 MHz) spectrum of compound **1** in $CDCl_3$. 12

   Figure S11. NOESY NMR (600 MHz) spectrum of compound **1** in $CDCl_3$. 13

   Figure S12. 1D NOE Spectrum of **1** in $CDCl_3$ (H-13, δ_H = 1.44 ppm)........................ 14

   Figure S13. 1D NOE Spectrum of **1** in $CDCl_3$ (H-15, δ_H = 1.94 ppm)........................ 15

   Figure S14. 1D NOE Spectrum of **1** in $CDCl_3$ (H-19, δ_H = 2.44 ppm)........................ 16

   Figure S15. ^{1}H NMR (600 MHz) spectrum of compound **1** in DMSO. 17

   Figure S16. IR spectrum of compound **1**. 18

   Figure S17. HR-ESIMS of compound **1**. 19

3. **Figure S18** ^{1}H NMR (600 MHz) spectrum of compound **2** in $CDCl_3$. 20

   Figure S19. ^{13}C NMR (600 MHz) spectrum of compound **2** in $CDCl_3$. 21

   Figure S20. HSQC NMR (600 MHz) spectrum of compound **2** in $CDCl_3$.................. 22

   Figure S21. HMBC NMR (600 MHz) spectrum of compound **2** in $CDCl_3$................. 23

   Figure S22. ^{1}H-^{1}H COSY NMR (600 MHz) spectrum of compound **2** in $CDCl_3$. 24

   Figure S23. NOESY NMR (600 MHz) spectrum of compound **2** in $CDCl_3$. 25

Figure S24. 1D NOE Spectrum of **2** in $CDCl_3$ (H-13, δ_H = 1.44 ppm)........................ 26

Figure S25. 1D NOE Spectrum of **2** in $CDCl_3$ (H-15, δ_H = 1.97 ppm)........................ 27

Figure S26. 1D NOE Spectrum of **2** in $CDCl_3$ (H-19, δ_H = 2.40 ppm)........................ 28

Figure S27. ^{1}H NMR (600 MHz) spectrum of compound **2** in DMSO. 29

Figure S28. IR spectrum of compound **2**. 30

Figure S29. HR-ESIMS of compound **2**. 31

4. **Figure S30.** ^{1}H NMR (600 MHz) spectrum of compound **3** in $CDCl_3$. 32

Figure S31. ^{13}C NMR (600 MHz) spectrum of compound **3** in $CDCl_3$. 33

Figure S32. HSQC NMR (600 MHz) spectrum of compound **3** in $CDCl_3$.................. 34

Figure S33. HMBC NMR (600 MHz) spectrum of compound **3** in $CDCl_3$................. 35

Figure S34. ^{1}H-^{1}H COSY NMR (600 MHz) spectrum of compound **3** in $CDCl_3$....... 36

Figure S35. NOESY NMR (600 MHz) spectrum of compound **3** in $CDCl_3$. 37

Figure S36. IR spectrum of compound **3**. 38

Figure S37. HR-ESIMS of compound **3**. 39

5. **Figure S38.** ^{1}H NMR (600 MHz) spectrum of compound **4** in $CDCl_3$. 40

Figure S39. ^{13}C NMR (600 MHz) spectrum of compound **4** in $CDCl_3$. 41

Figure S40. HSQC NMR (600 MHz) spectrum of compound **4** in $CDCl_3$.................. 42

Figure S41. HMBC NMR (600 MHz) spectrum of compound **4** in $CDCl_3$................. 43

Figure S42. ^{1}H-^{1}H COSY NMR (600 MHz) spectrum of compound **4** in $CDCl_3$....... 44

Figure S43. NOESY NMR (600 MHz) spectrum of compound **4** in $CDCl_3$. 45

Figure S44. IR spectrum of compound **4**. 46

Figure S45. HR-ESIMS of compound **4**. 47

6. **Figure S46.** ^{1}H NMR (600 MHz) spectrum of compound **5** in $CDCl_3$. 48

Figure S47. ^{13}C NMR (600 MHz) spectrum of compound **5** in $CDCl_3$. 49

Figure S48. HSQC NMR (600 MHz) spectrum of compound **5** in $CDCl_3$.................. 50

Figure S49. HMBC NMR (600 MHz) spectrum of compound **5** in $CDCl_3$................. 51

Figure S50. IR spectrum of compound **5**. 52

Figure S51. HR-ESIMS of compound **5**. 53

7. Table S1. Bacterial strains used in the antimicrobial screening assays. 54

Table S2. Cartesian coordinates of the low energy reoptimized conformers of **1** calculated at B3LYP/6 31+G(d) level of theory. 55

Table S3. Cartesian coordinates of the low energy reoptimized conformers of **2** calculated at B3LYP/6 31+G(d) level of theory. 58

Gene annotations of gene cluster *ascw*

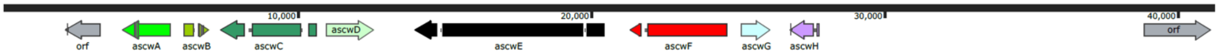

no	description	microorganism	coverage	identity	accession	size(aa)	location
ascwA	cytochrome p450	*Acremonium egyptiacum*	96%	78.57%	A0A455R4X0.1	535	4034-5691
ascwB	terpene cyclase	*Acremonium egyptiacum*	95%	68.27%	A0A455RAK9.1	227	6168-7013
ascwC	cytochrome p450	*Acremonium egyptiacum*	100%	73.03%	A0A455R5H4.1	960	7387-10668
ascwD	flavin-dependent halogenase	*Acremonium egyptiacum*	99%	83.78%	A0A455R7M0.1	553	10988-12649
ascwE	polyketide synthase	*Acremonium egyptiacum*	99%	69.81%	A0A455R5P9.1	2083	13990-20437
ascwF	non-ribosomal peptide synthetase	*Acremonium egyptiacum*	100%	73.88%	A0A455R7E6.1	1055	21328-24649
ascwG	prenyl transferase	*Acremonium egyptiacum*	99%	78.76%	A0A455R413.1	340	25122-26144
ascwH	transcriptional factor	*Acremonium egyptiacum*	100%	50.47%	A0A455R614.1	283	26598-28082

Figure S1. Identification of the *ascw* gene cluster in *Acremonium* sp. (GenBank Accession number PP795974).

Gene annotations of gene cluster *ascw* which was discovered by antiSMASH (https://antismash.secondarymetabolites.org/#!/start) and predicted at softberry (https://www.softberry.com/).

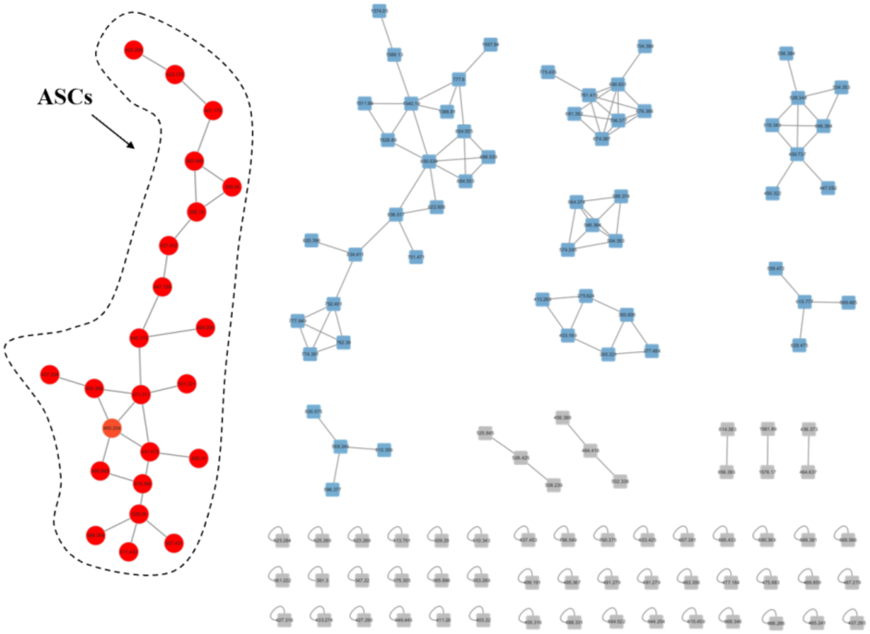

Figure S2. GNPS Molecular Networking analysis of *Acremonium* sp.

The resultant networking is visualized in Cytoscape (version 3.6.1), where one node represents one consensus MS-MS spectrum and is labeled with the precursor mass. The figure above shows the analysis results of the crude extract. The red node is a cluster of ASCs compounds, among them, the blue nodes represent compounds that form clusters. Grey nodes represent some unknown and individual compounds.

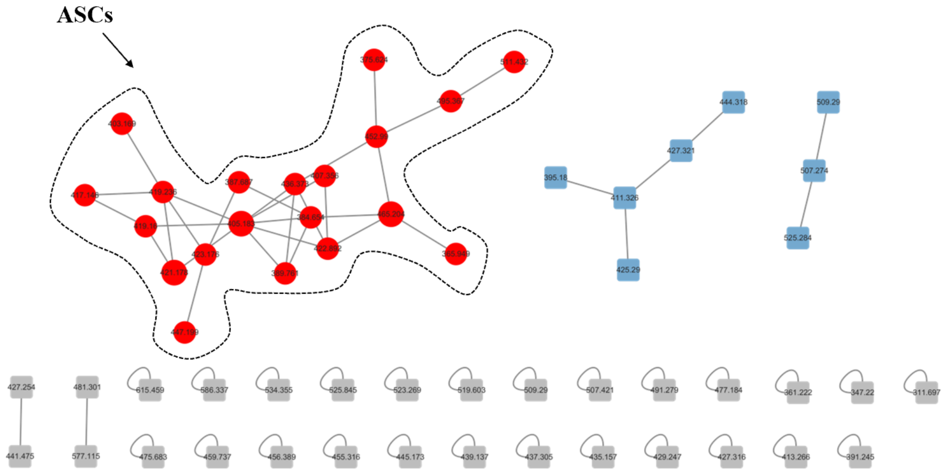

Figure S3. GNPS Molecular Networking of ASCs in *Acremonium* sp.

The figure above shows the analysis results of Fraction 2 which was eluted with 100% dichloromethane. The specific colors have the same meanings as in Figure 2.

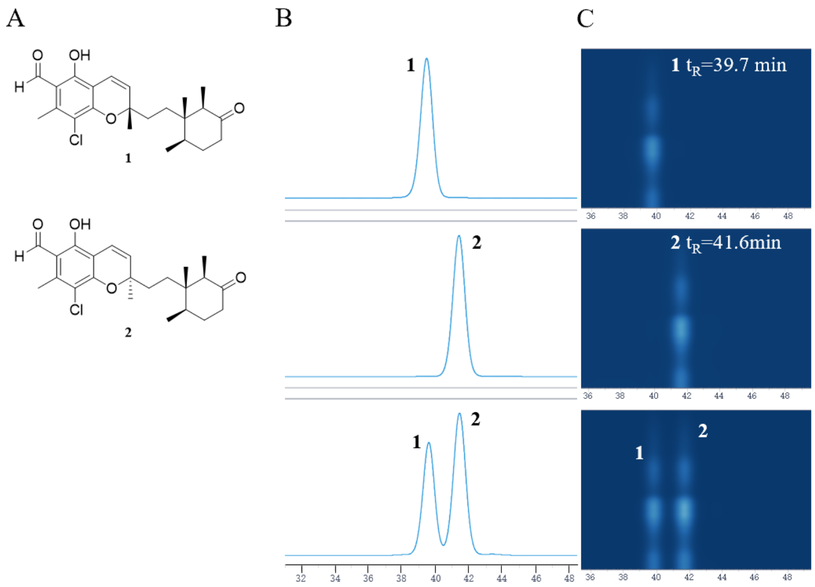

Figure S4. HPLC identification of compounds **1** and **2**.

(A) Structure of compounds **1** and **2**; (B) Retention times of compounds **1** and **2** via HPLC (65:35 MeCN−H_2O with 0.1% Formic acid, 1 mL/min, 270 nm) using an ODS column (X-bridge C_{18} column, 4.6 × 250 mm, 5 μm); (C) UV full wavelength scan spectra of compounds **1** and **2**.

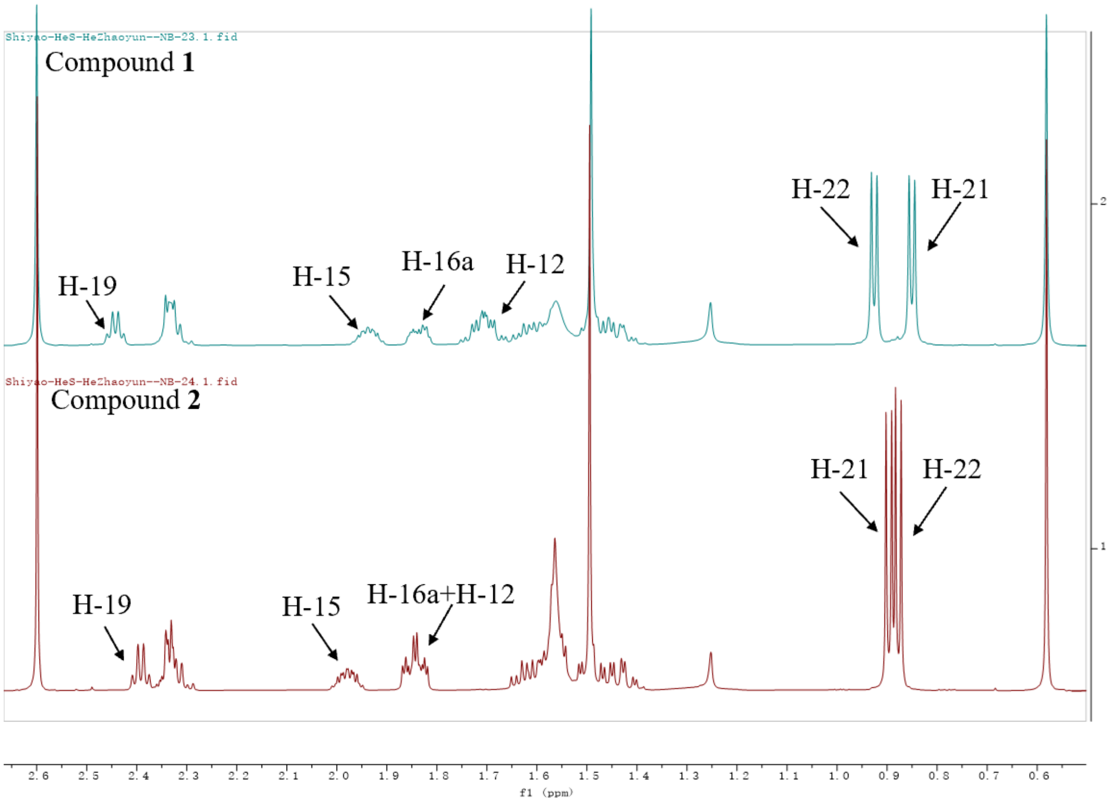

Figure S5. Comparison of ^{1}H NMR spectra of compounds **1** and **2** in $CDCl_3$.

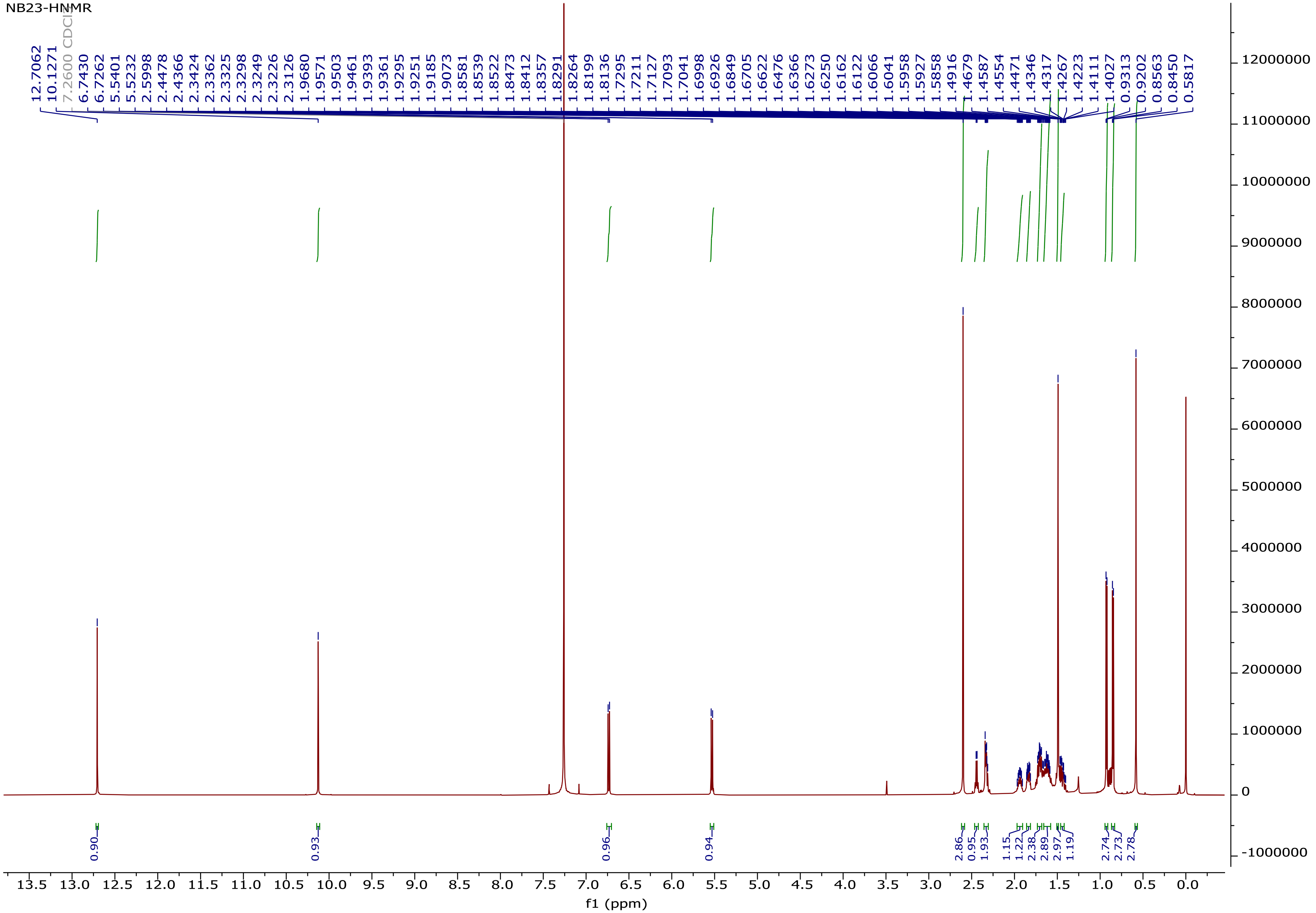

Figure S6. ^{1}H NMR Spectrum of **1** in $CDCl_3$.

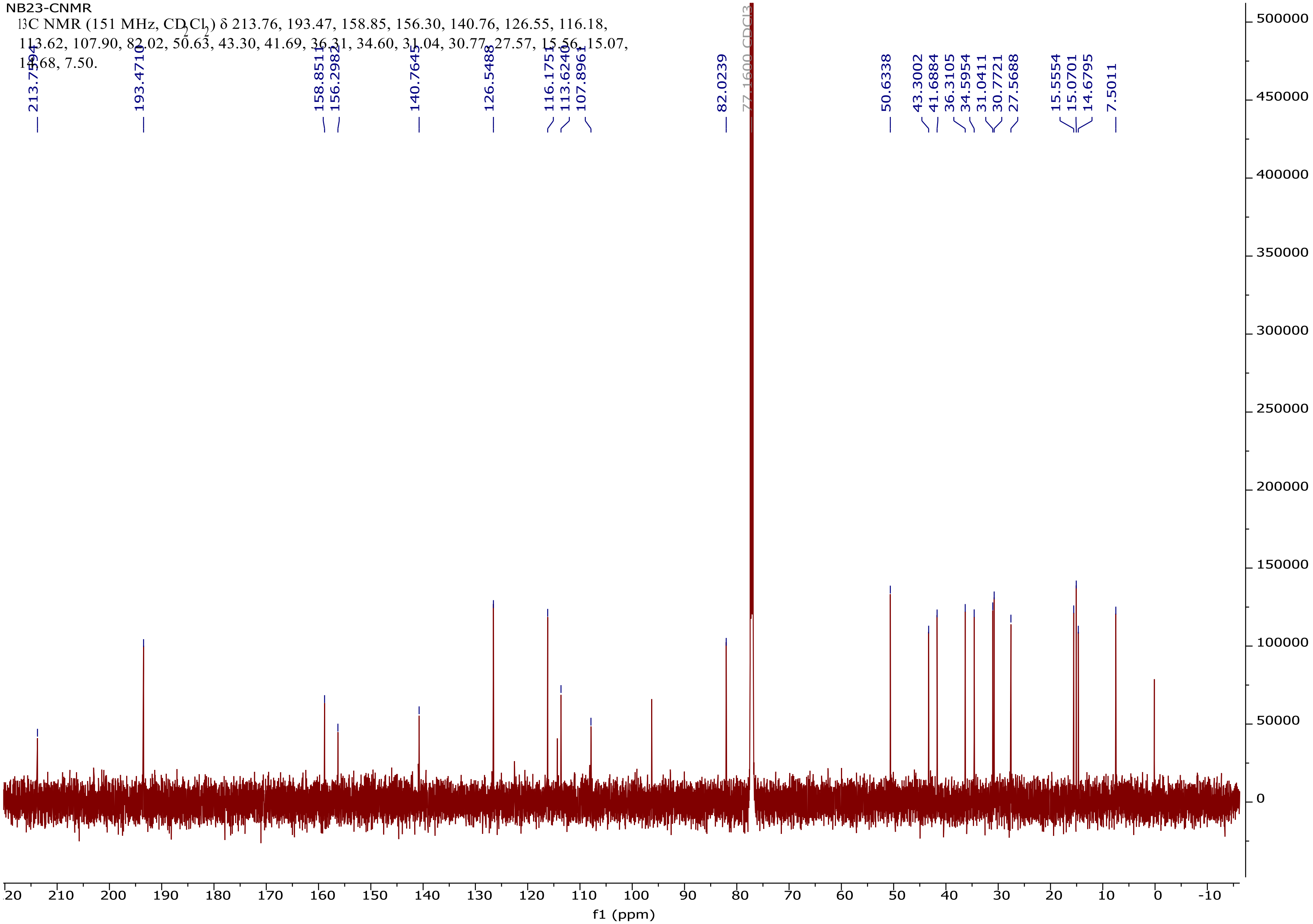

Figure S7. ^{13}C NMR Spectrum of **1** in $CDCl_3$.

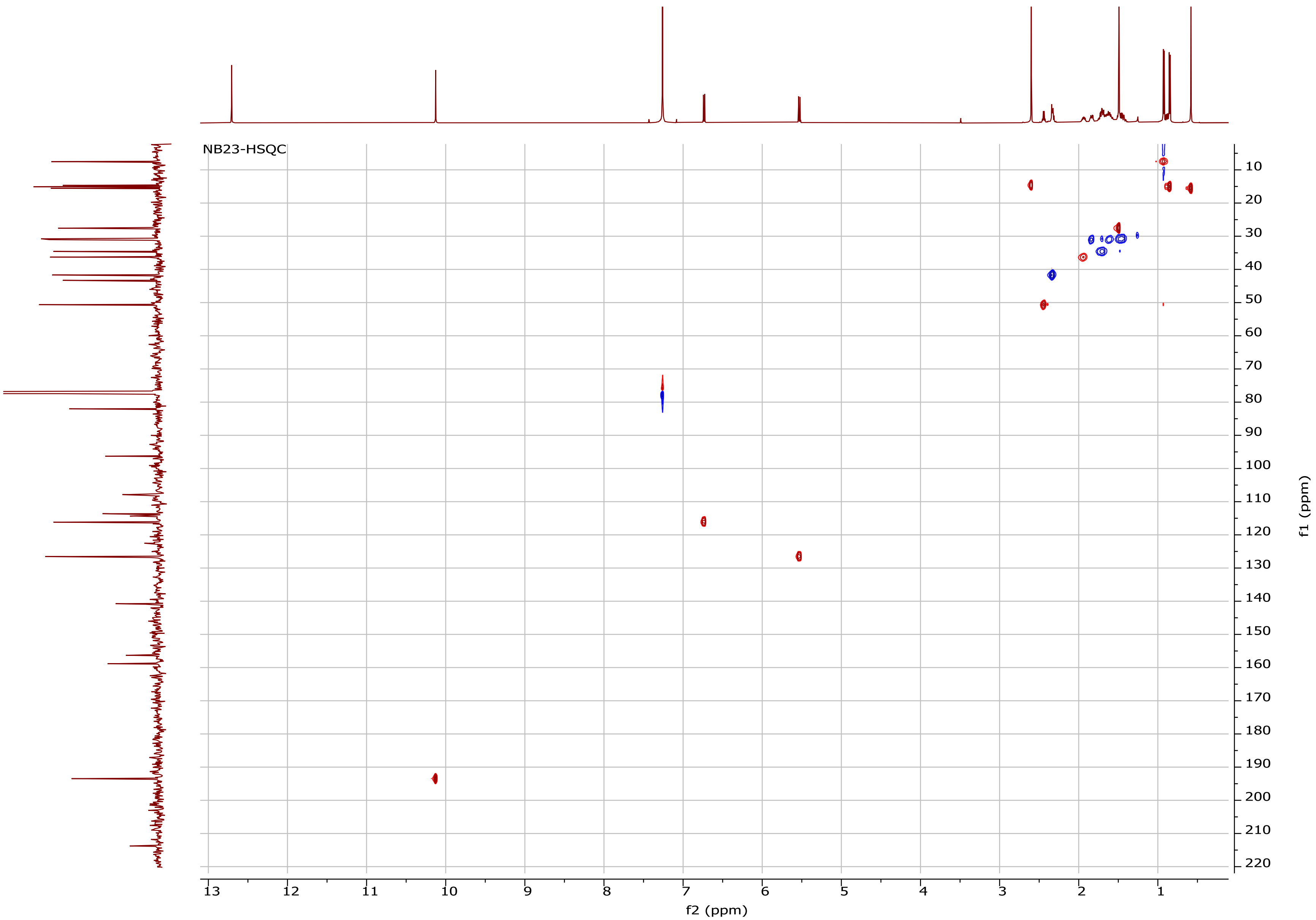

Figure S8. HSQC Spectrum of **1** in $CDCl_3$.

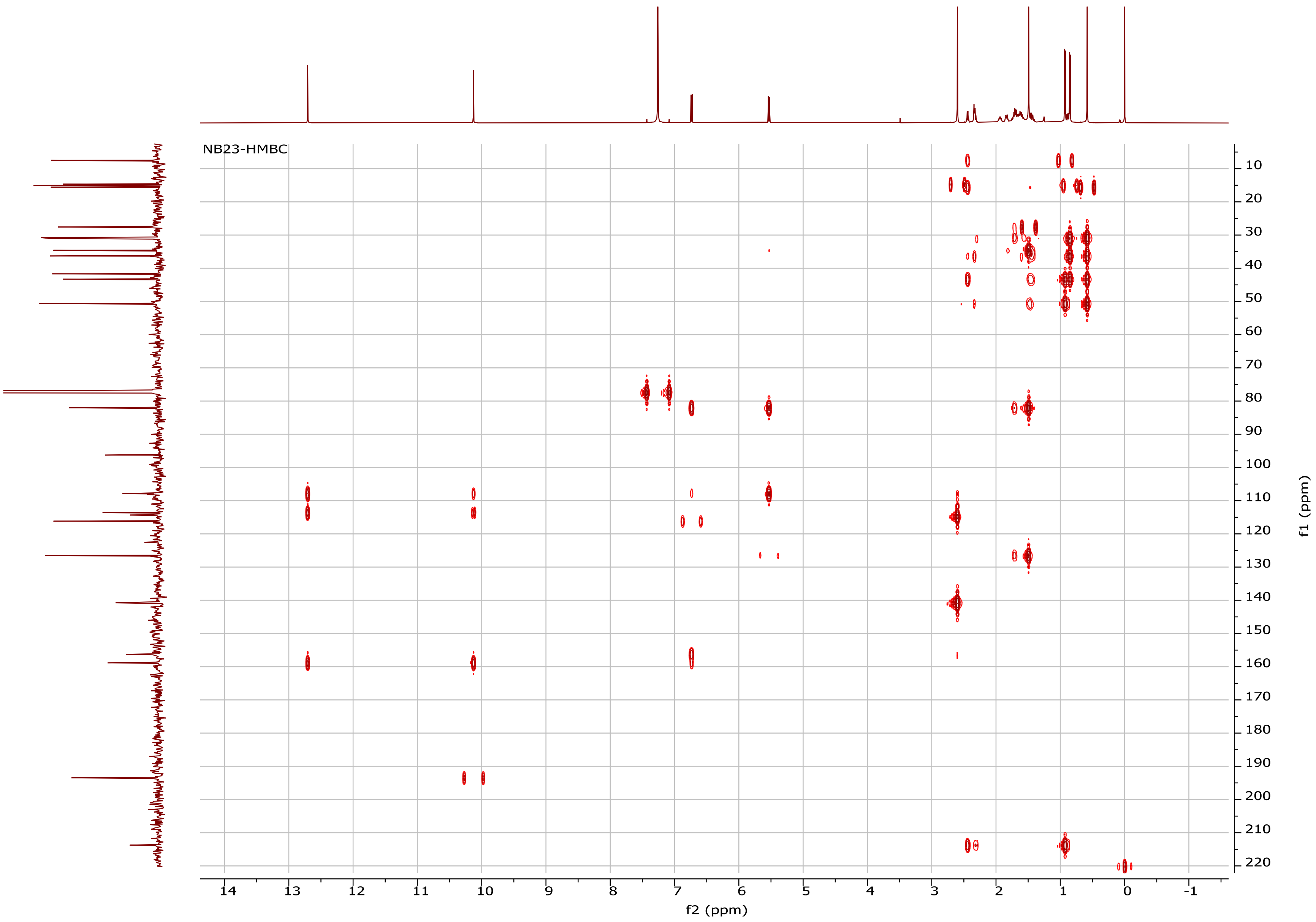

Figure S9. HMBC Spectrum of **1** in $CDCl_3$.

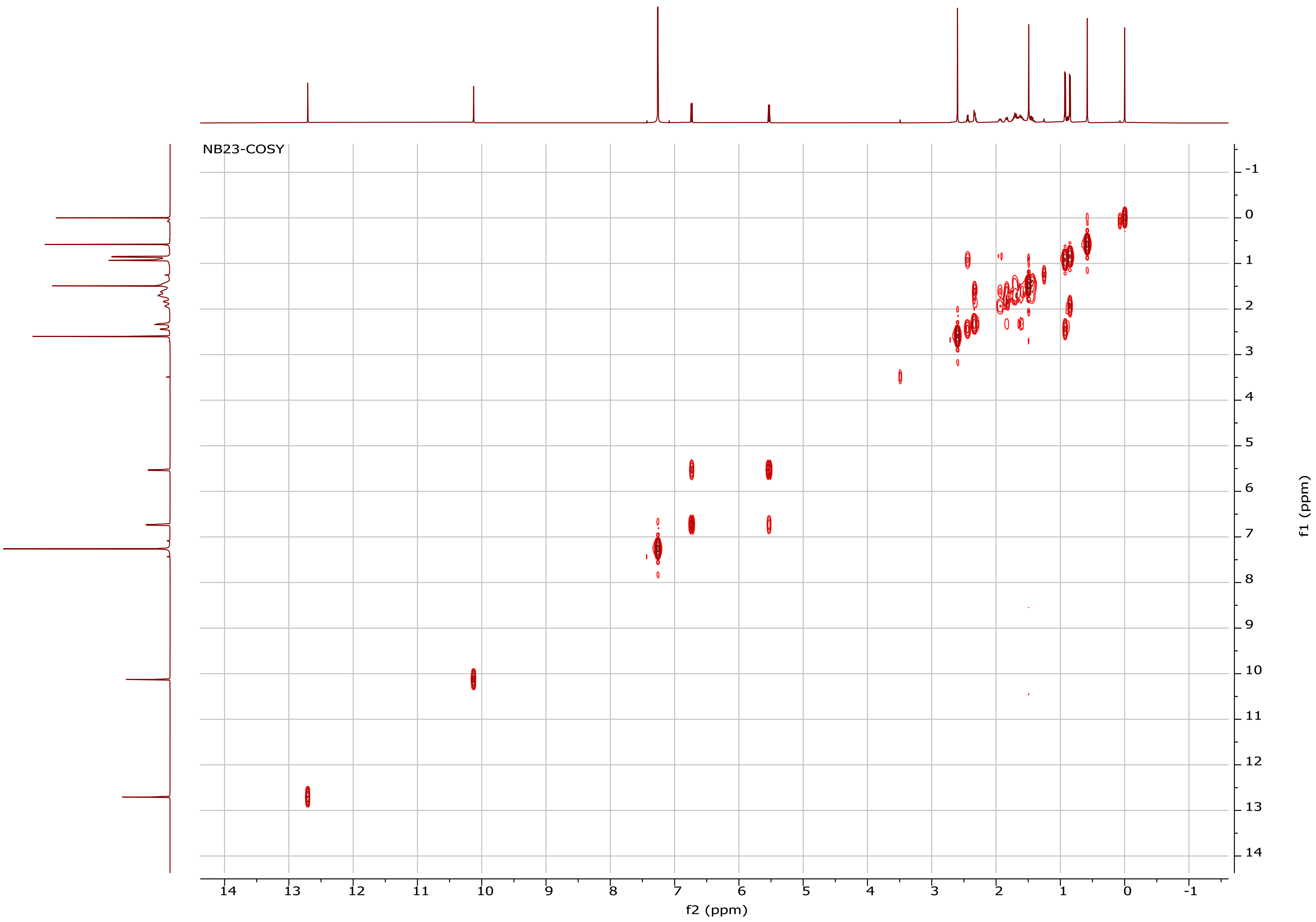

Figure S10. COSY Spectrum of **1** in $CDCl_3$.

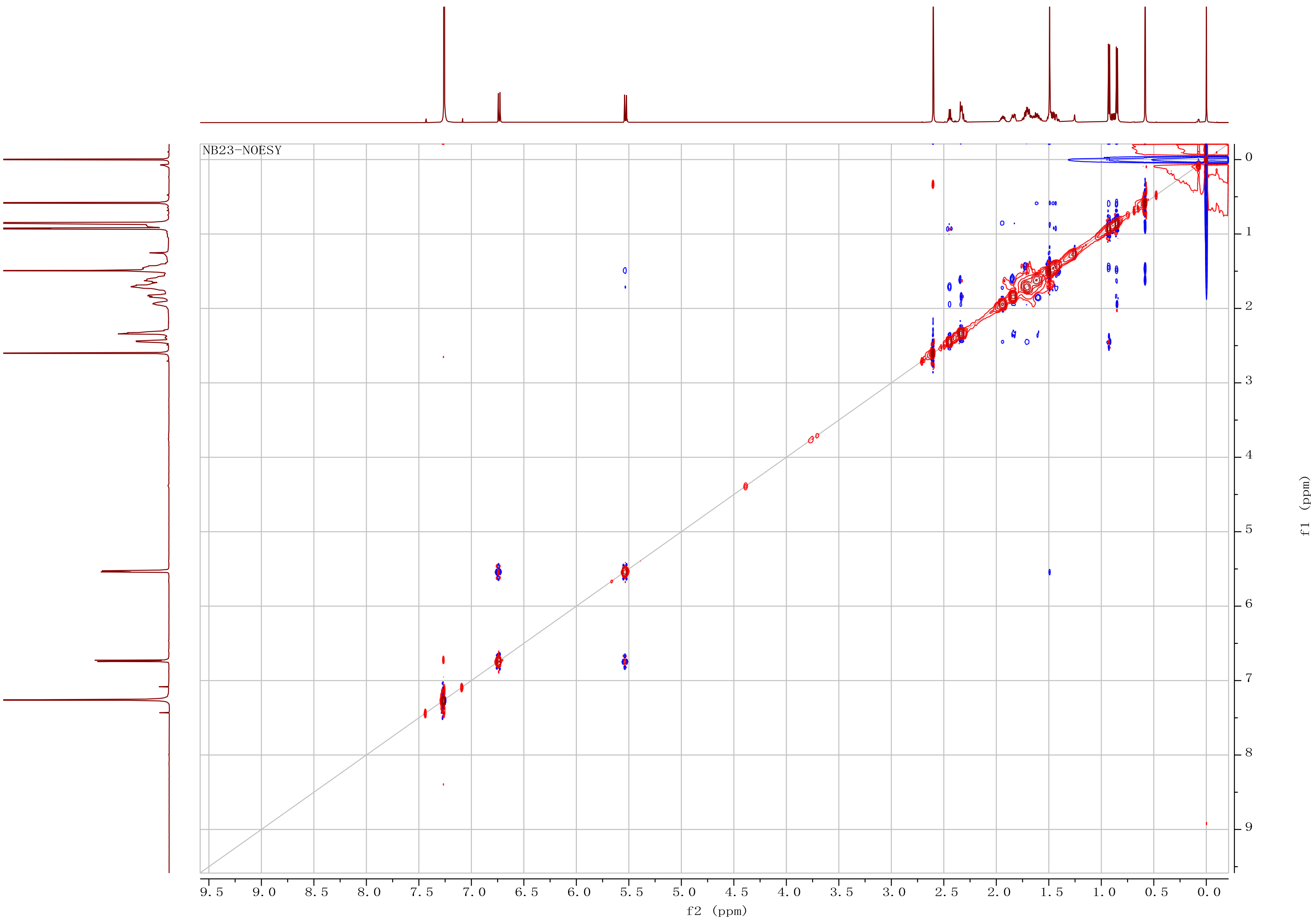

Figure S11. NOESY Spectrum of **1** in $CDCl_3$.

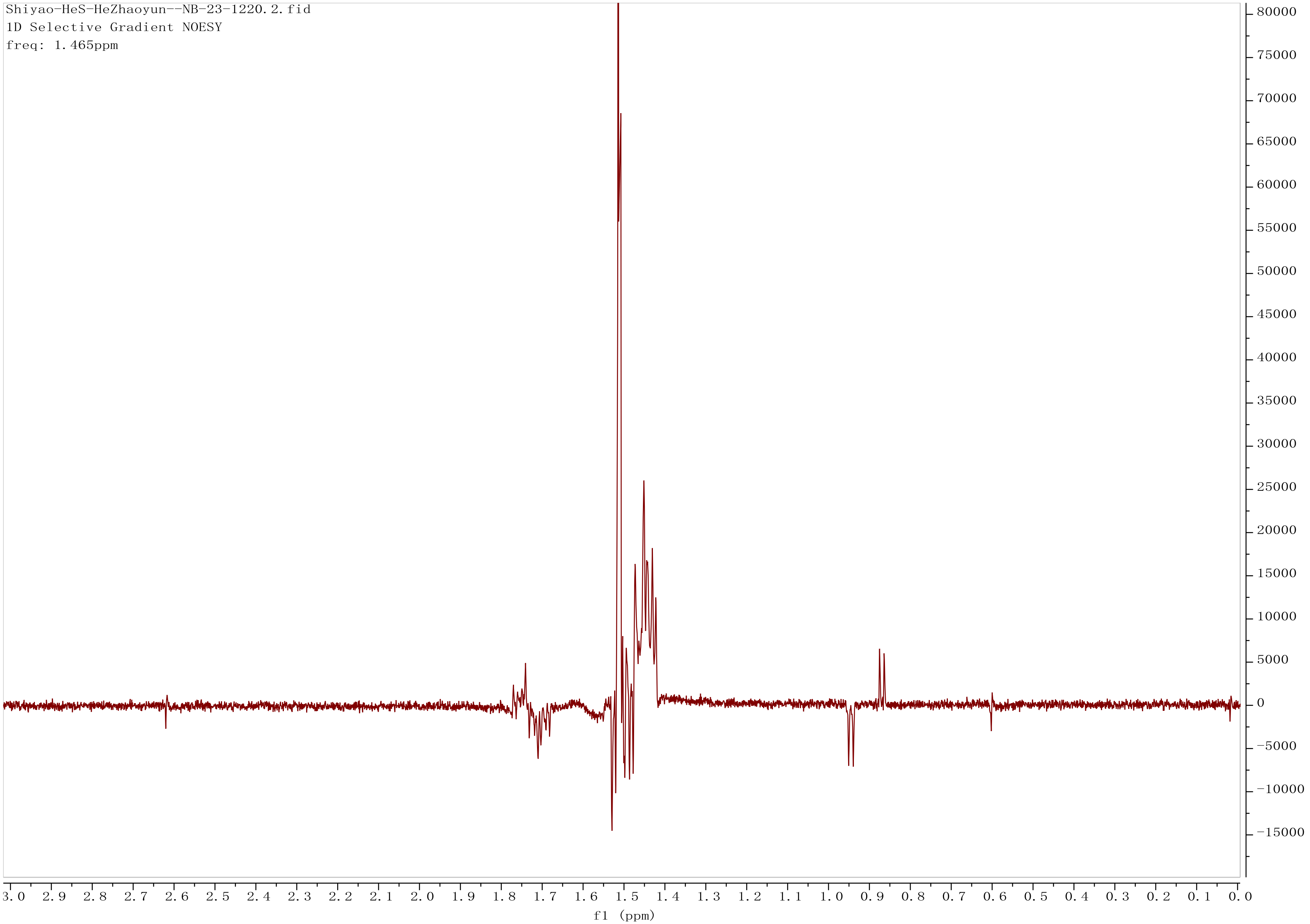

Figure S12. 1D NOE Spectrum of **1** in $CDCl_3$ (H-13, δ_H = 1.44 ppm)

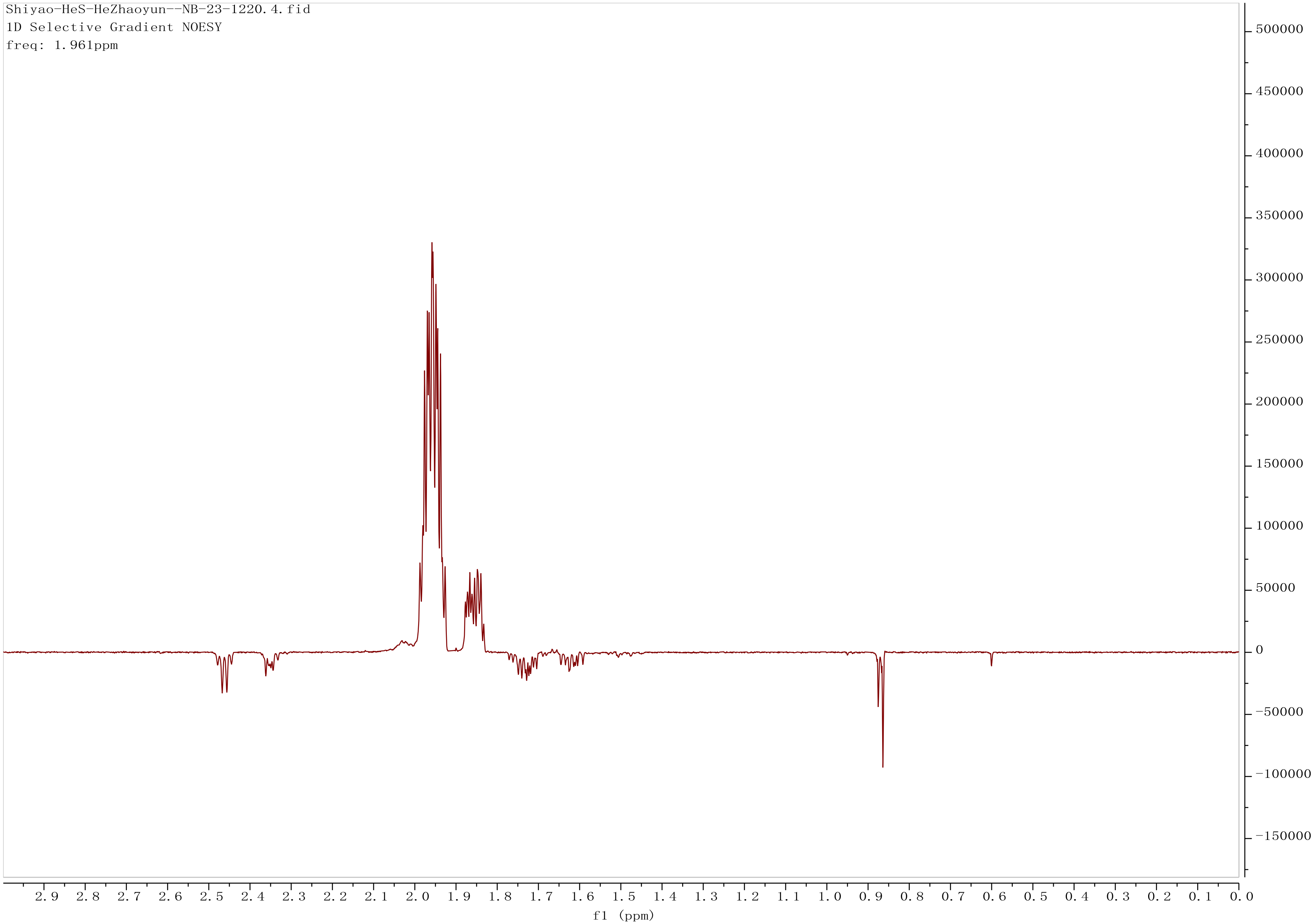

Figure S13. 1D NOE Spectrum of **1** in $CDCl_3$ (H-15, δ_H = 1.94 ppm)

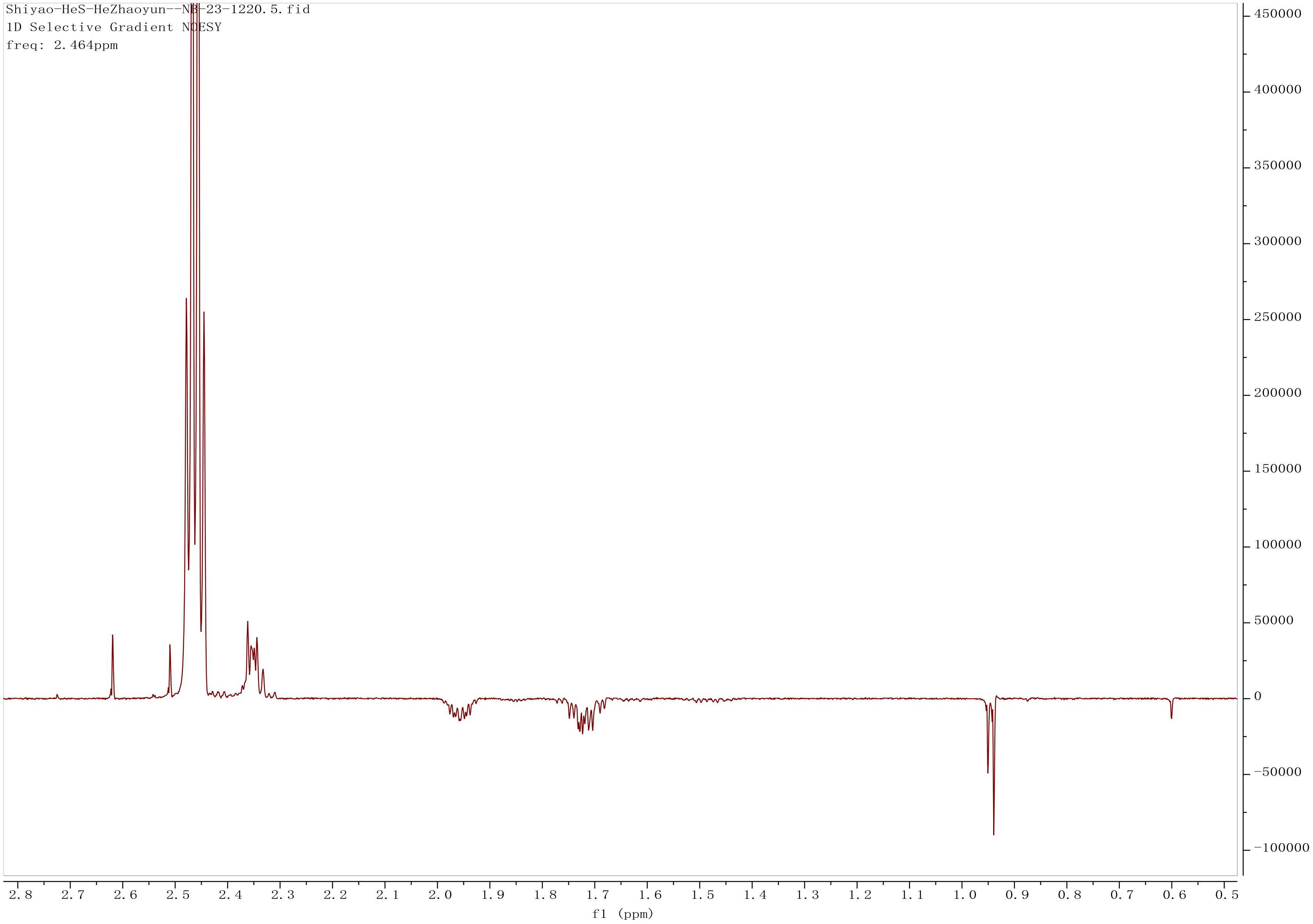

Figure S14. 1D NOE Spectrum of **1** in $CDCl_3$ (H-19, δ_H = 2.44 ppm)

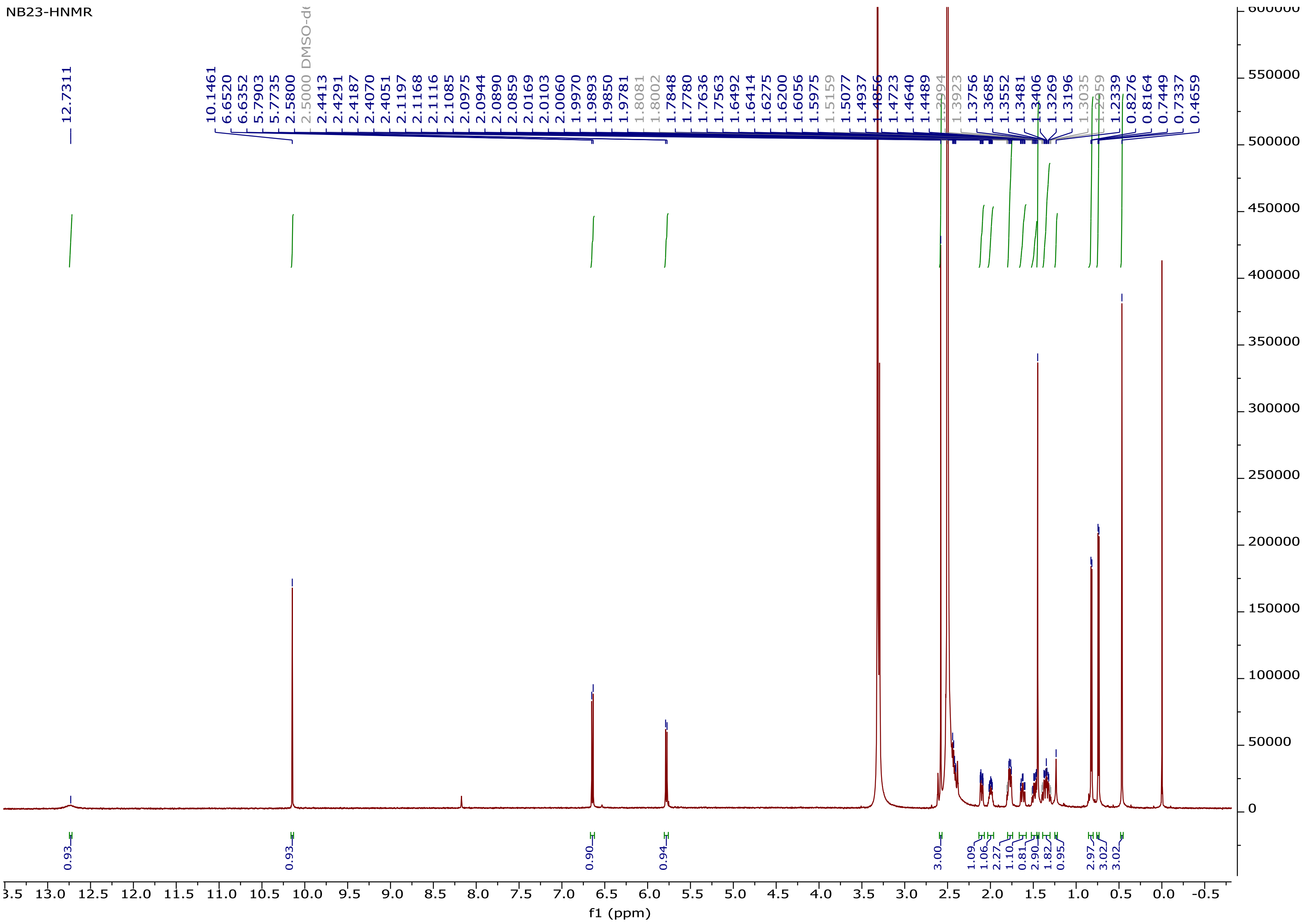

Figure S15. ^{1}H NMR Spectrum of **1** in DMSO.

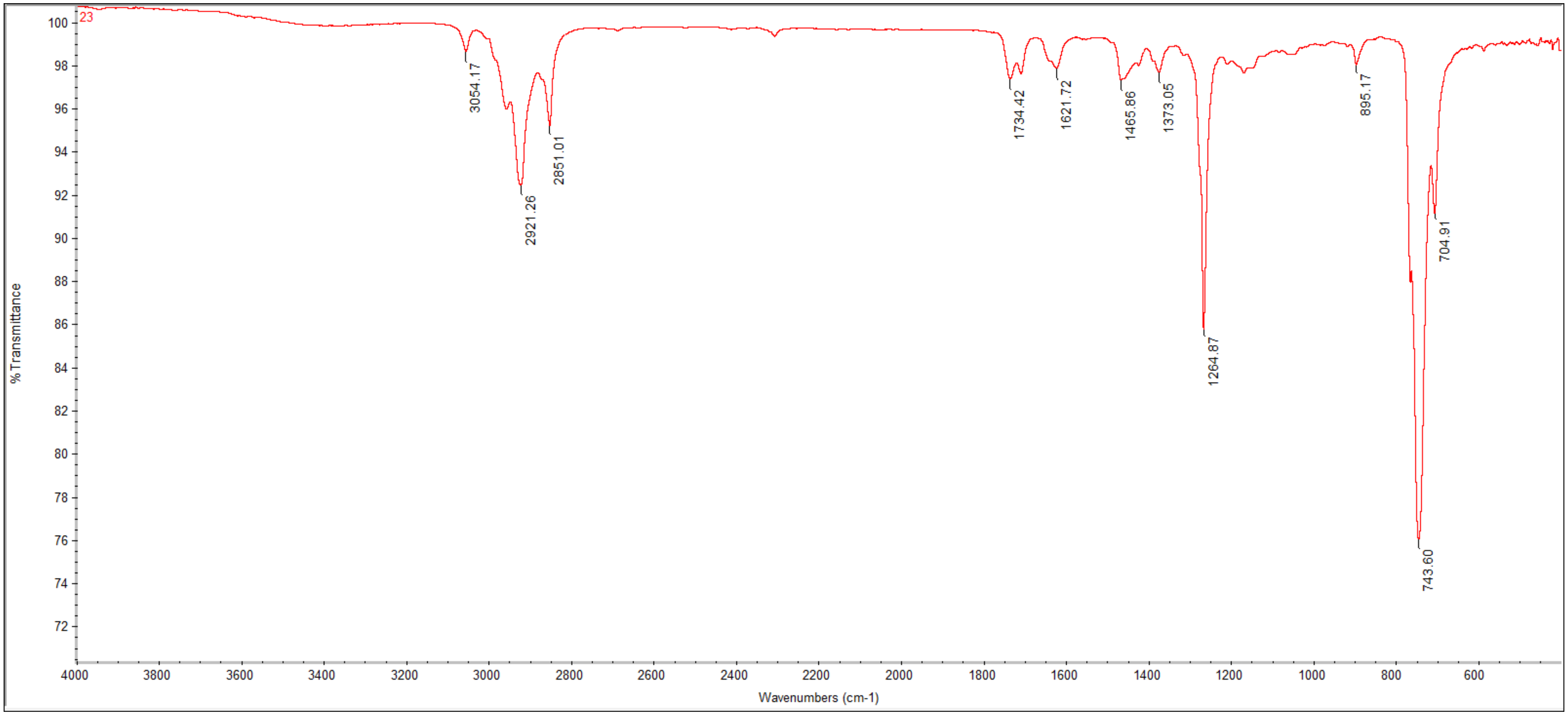

Figure S16. IR spectrum of compound **1**.

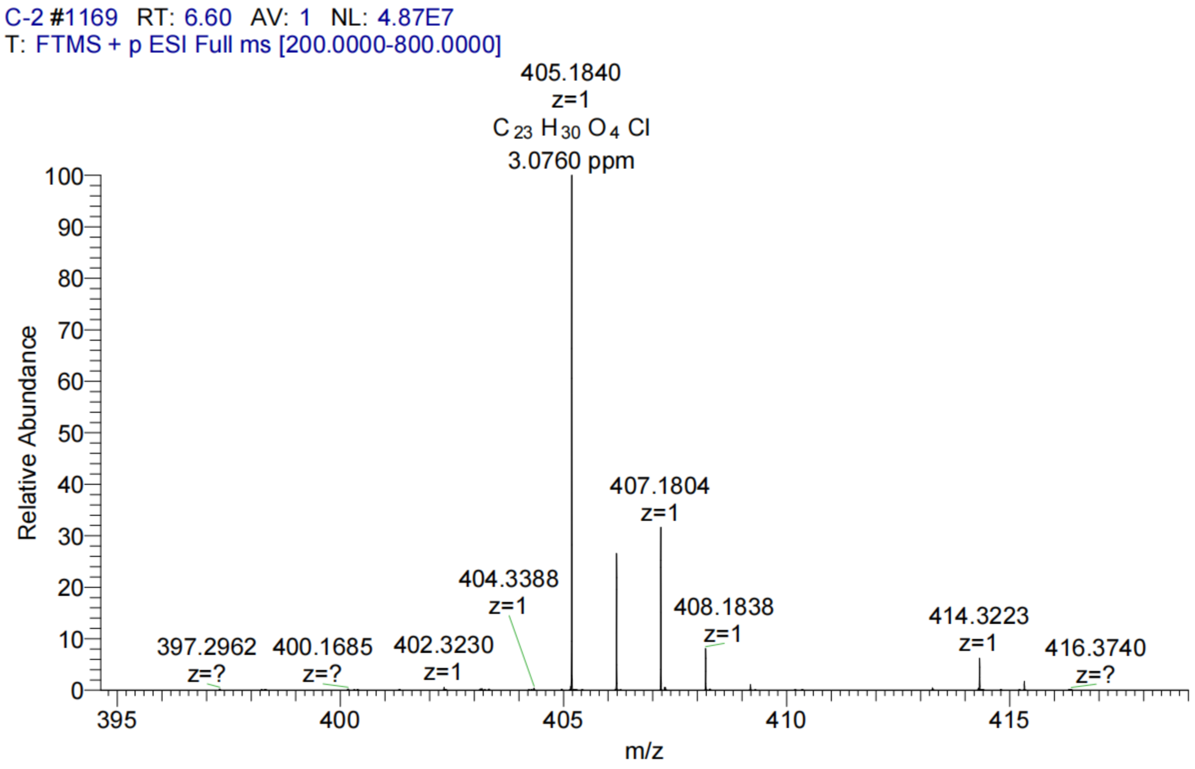

Figure S17. HR-ESIMS of compound **1**.

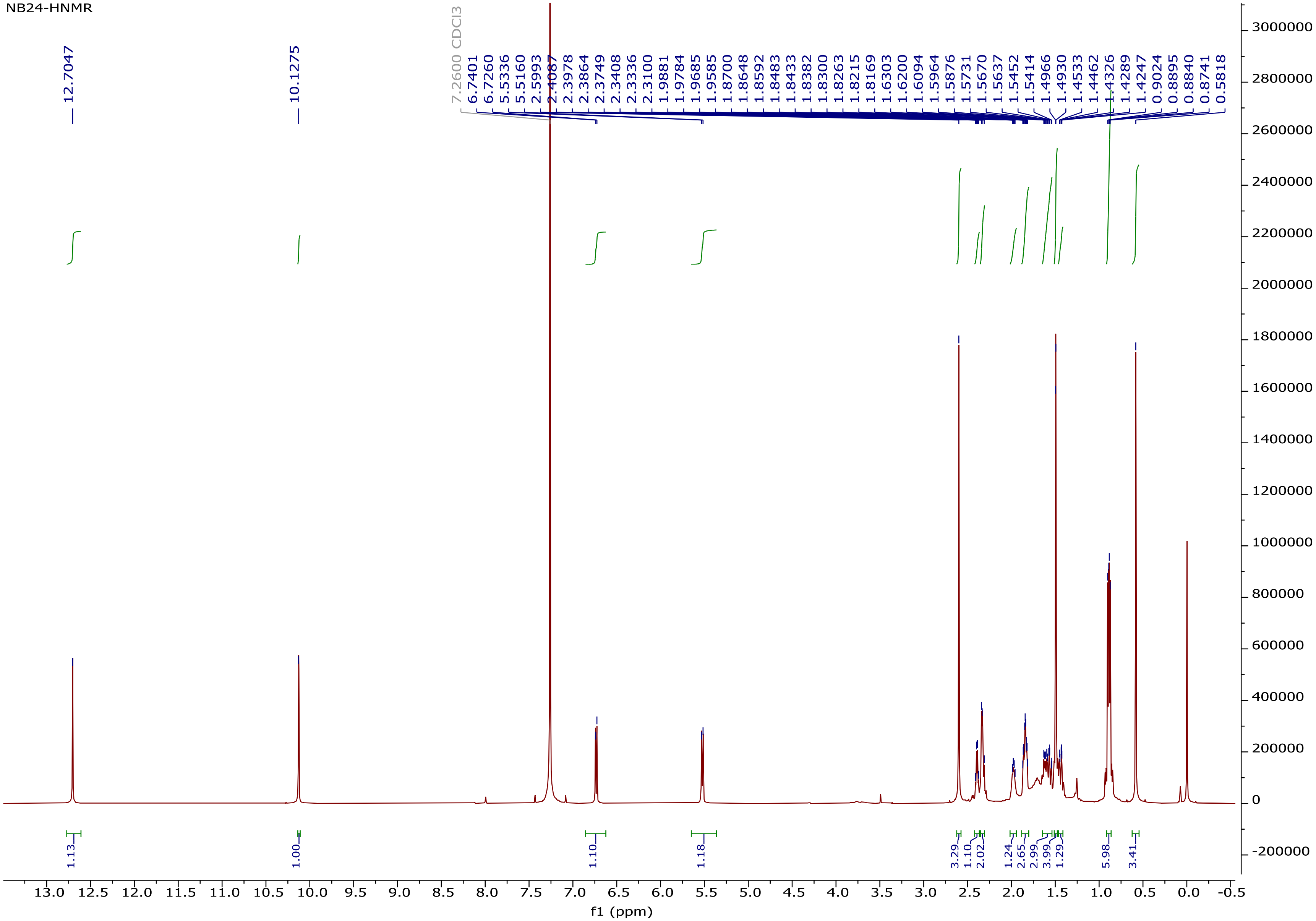

Figure S18. ^{1}H NMR Spectrum of **2** in $CDCl_3$.

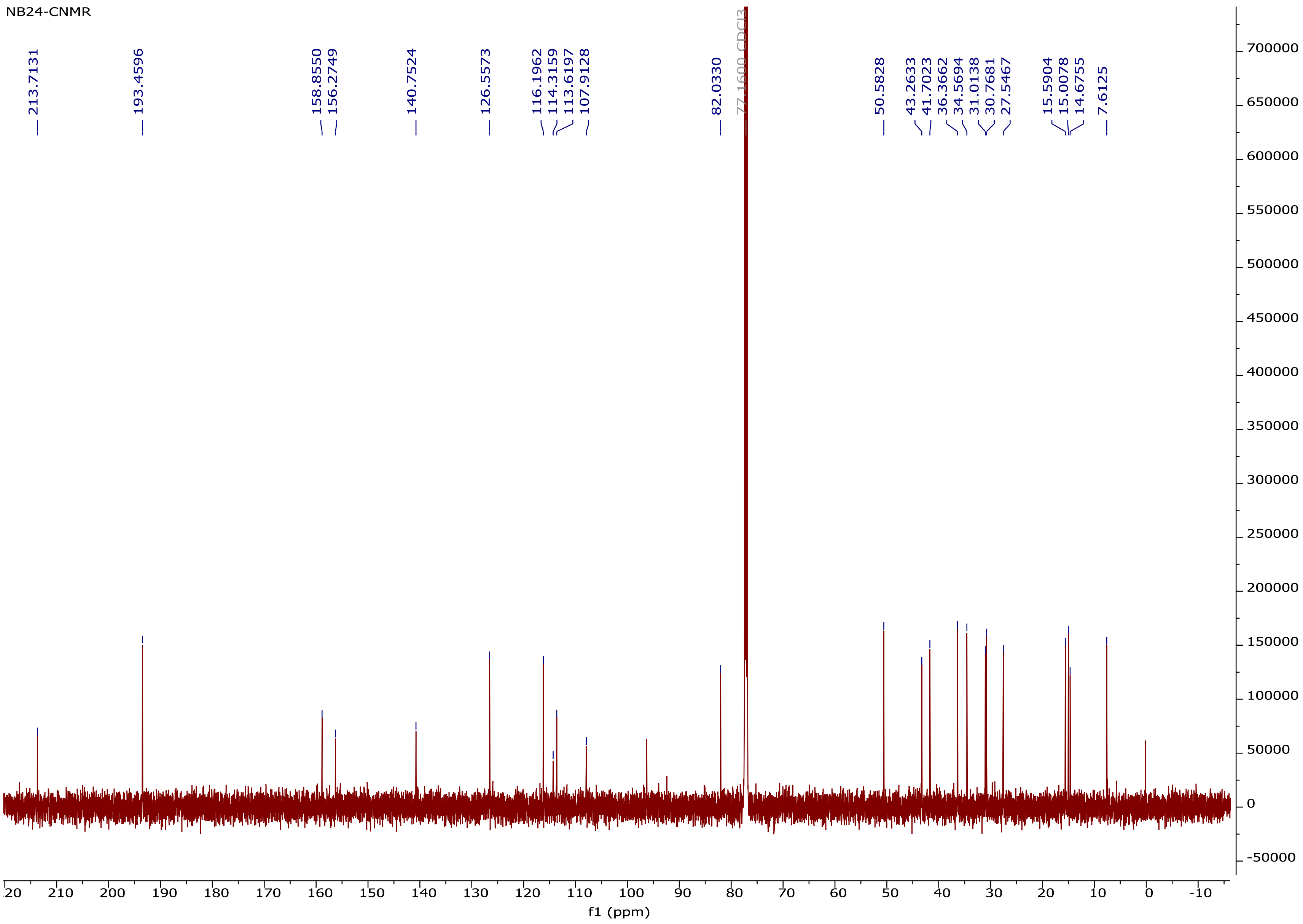

Figure S19. ^{13}C NMR Spectrum of **2** in $CDCl_3$.

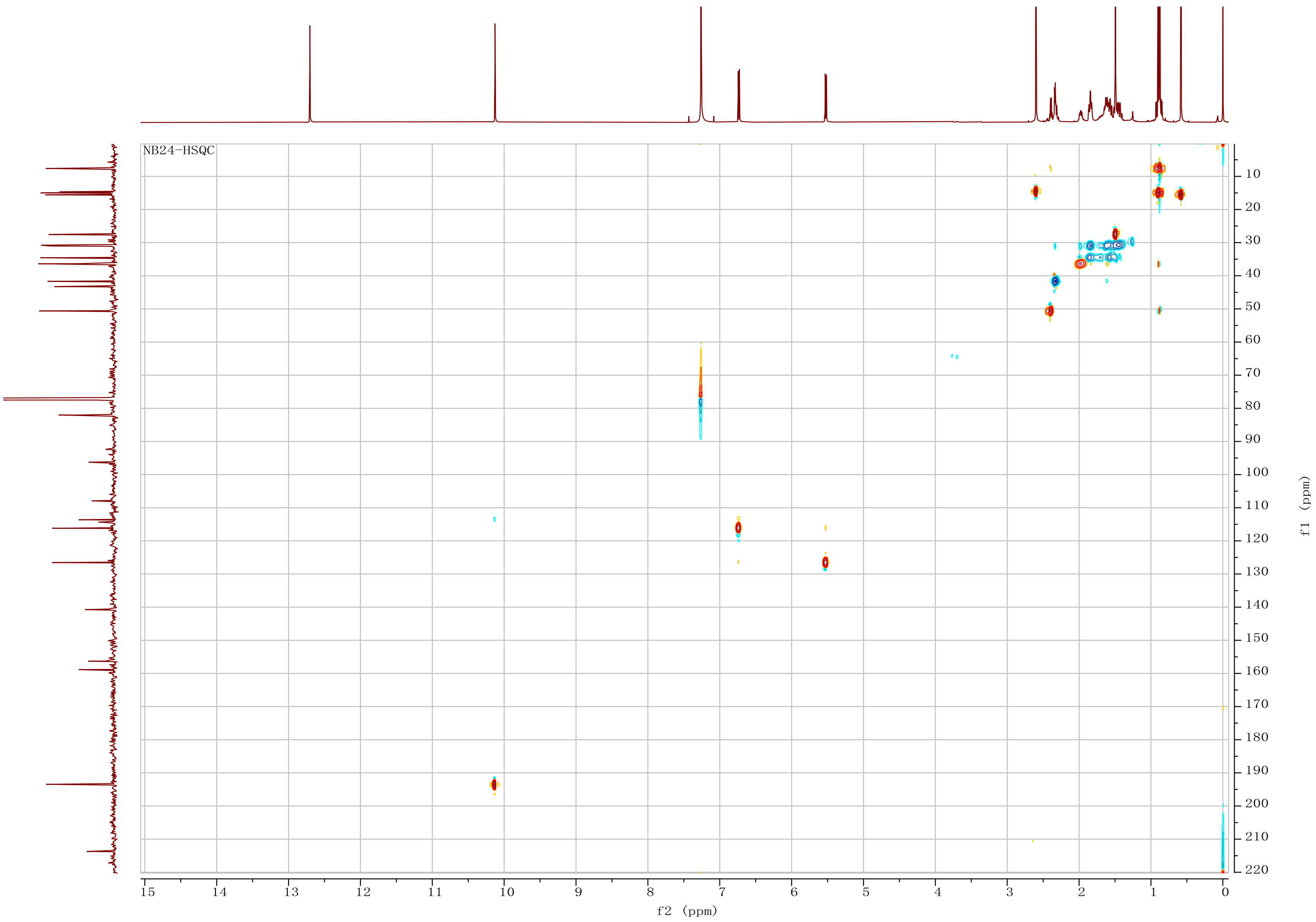

Figure S20. HSQC Spectrum of **2** in $CDCl_3$.

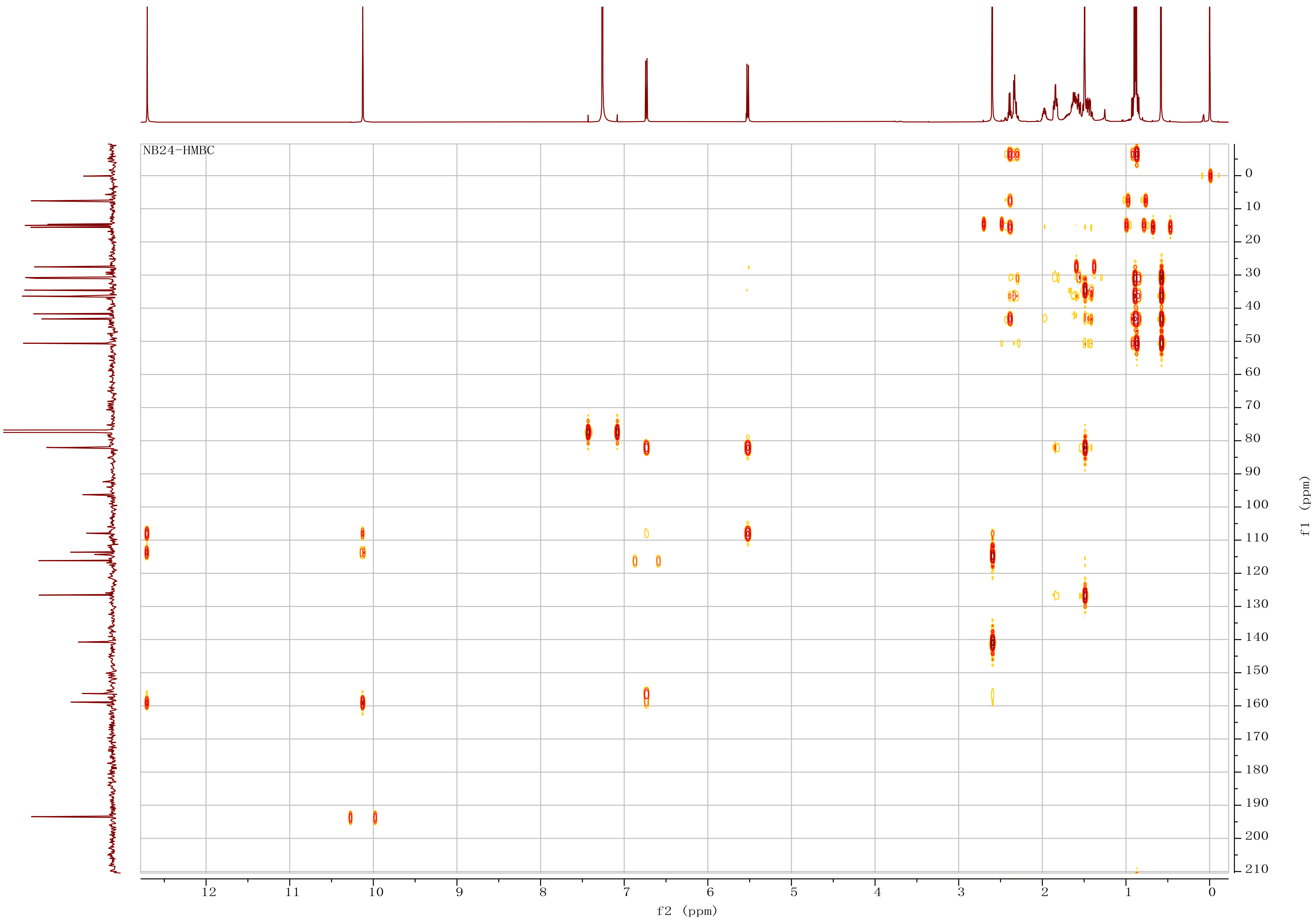

Figure S21. HMBC Spectrum of **2** in $CDCl_3$.

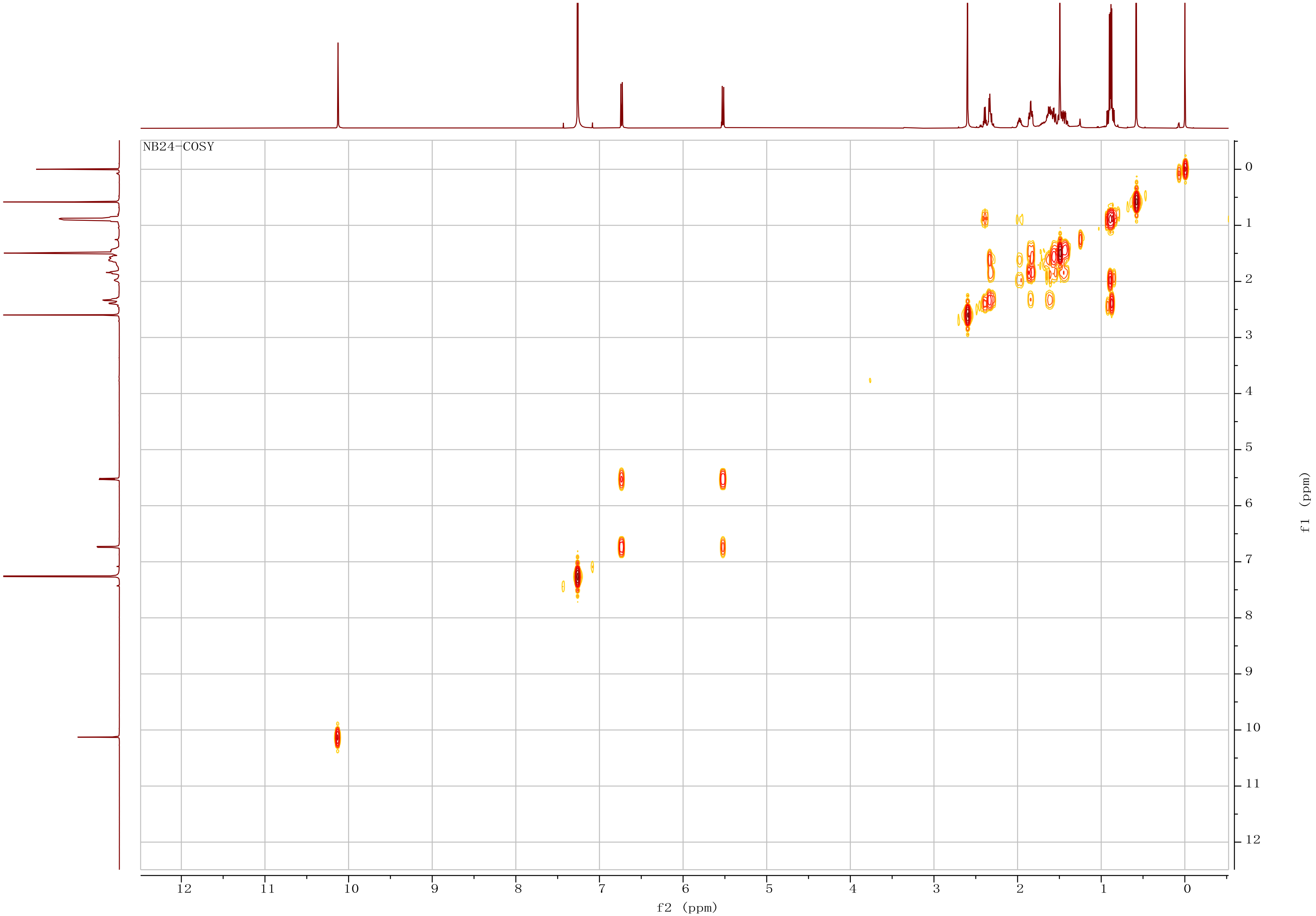

Figure S22. COSY Spectrum of **2** in $CDCl_3$.

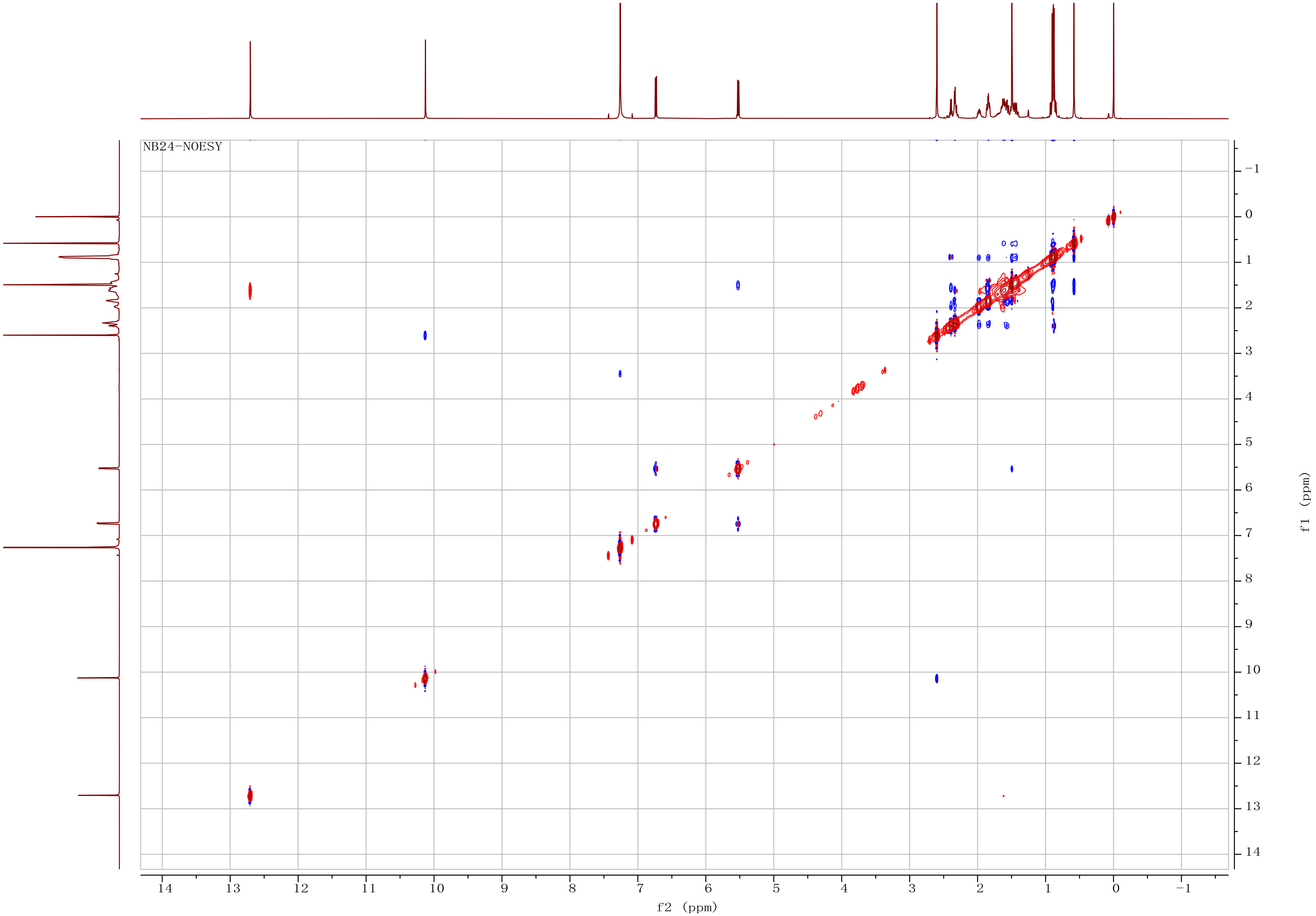

Figure S23. NOESY Spectrum of **2** in $CDCl_3$.

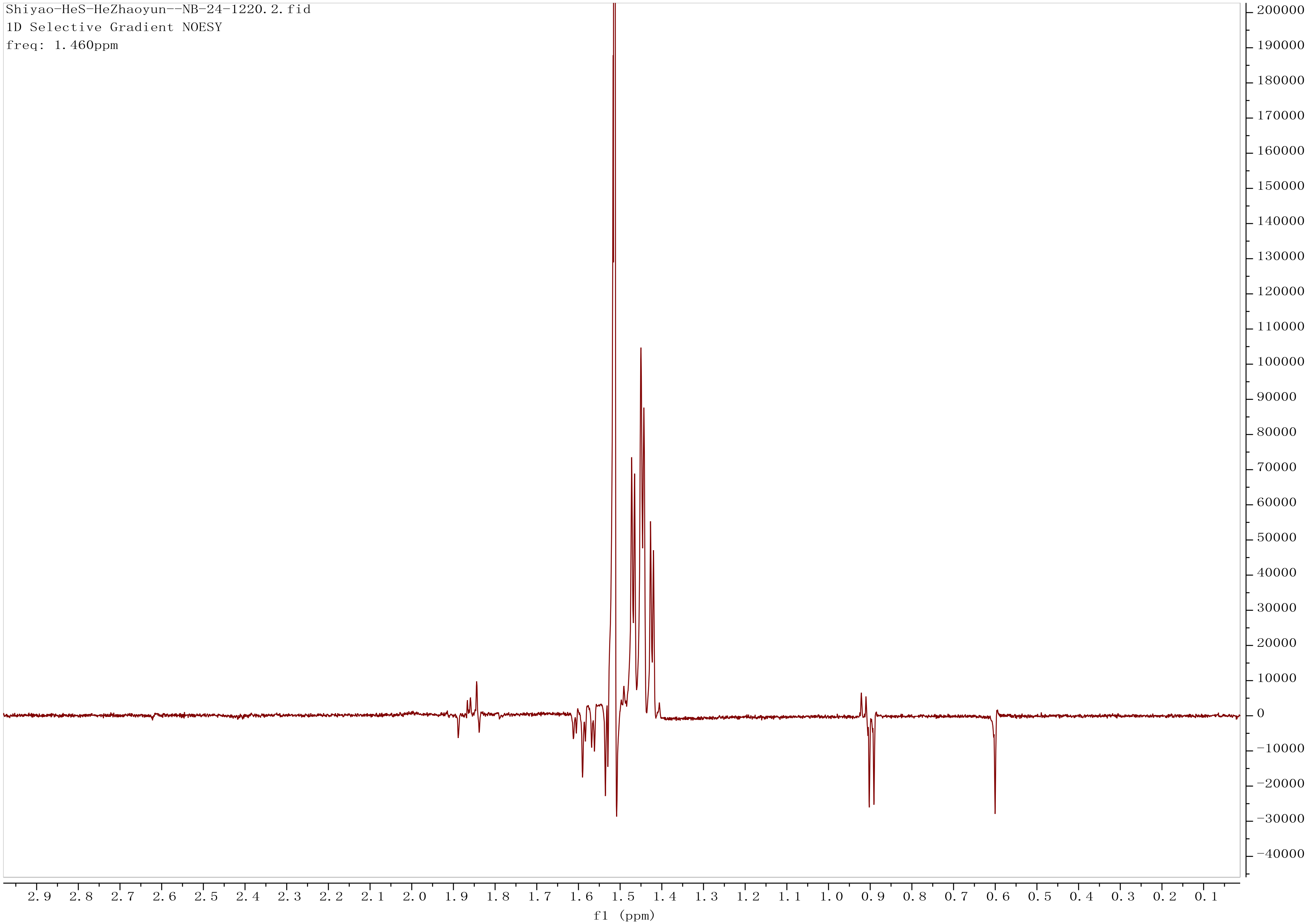

Figure S24. 1D NOE Spectrum of **2** in $CDCl_3$ (H-13, δ_H = 1.44 ppm)

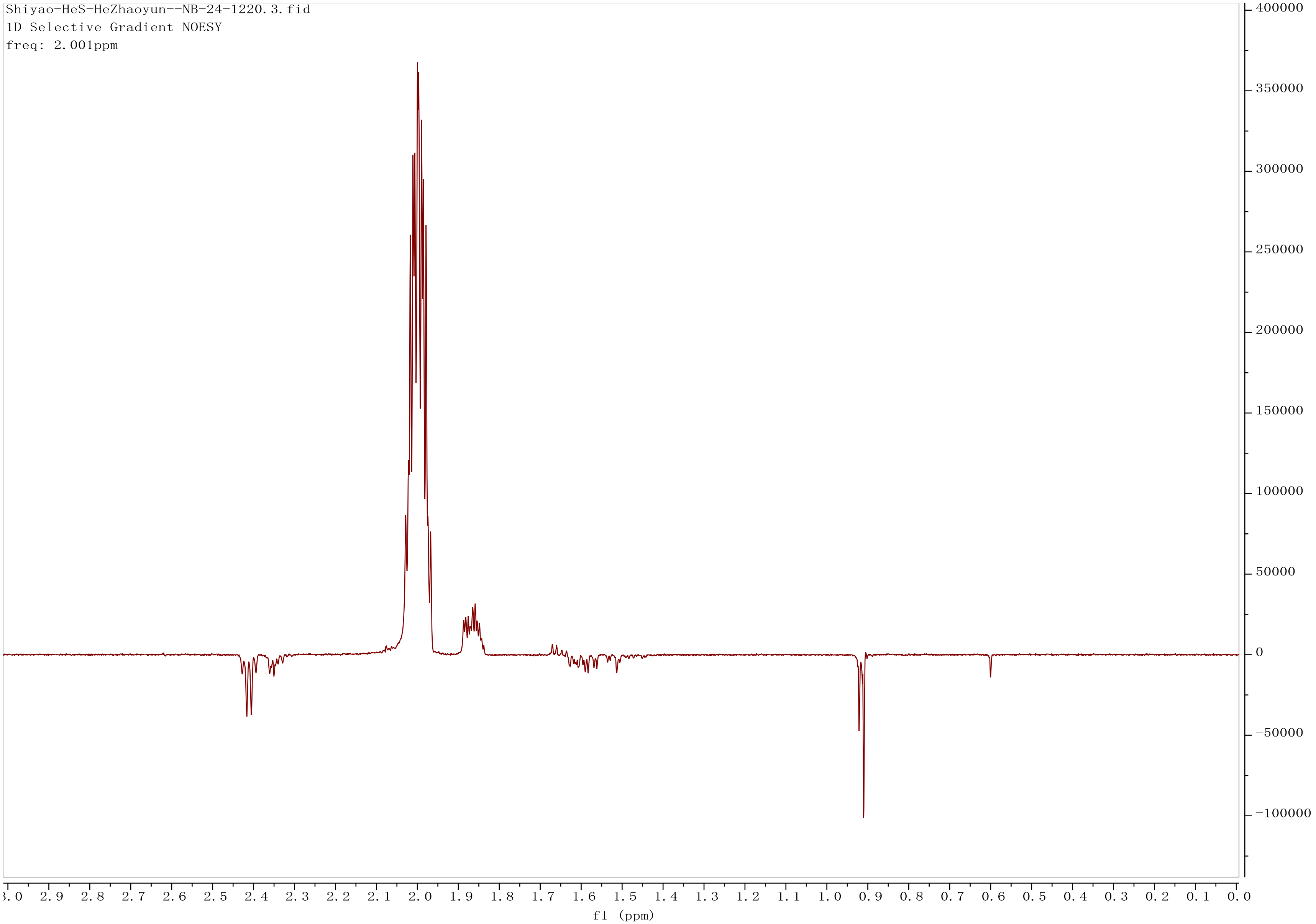

Figure S25. 1D NOE Spectrum of **2** in $CDCl_3$ (H-15, δ_H = 1.97 ppm)

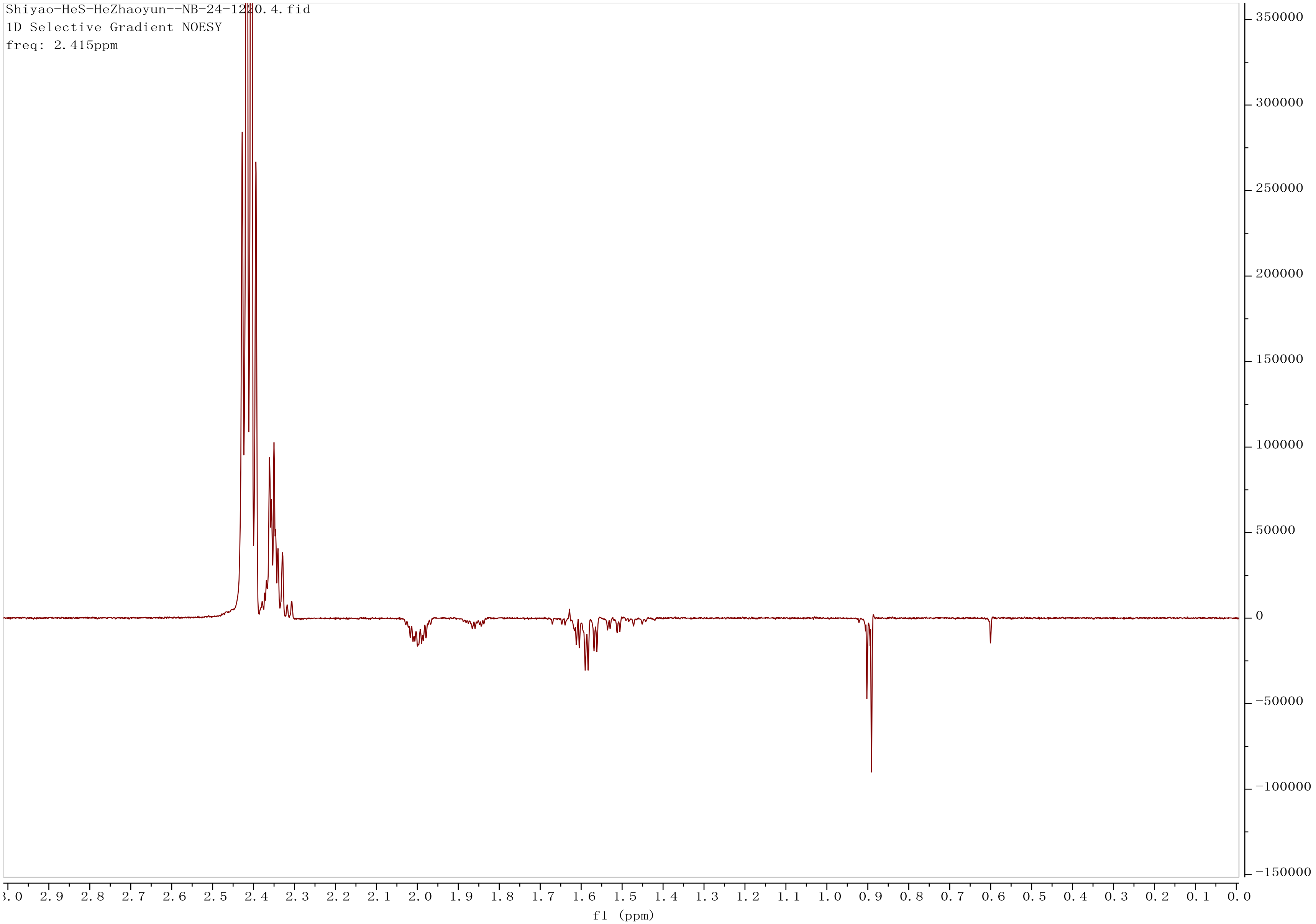

Figure S26. 1D NOE Spectrum of **2** in $CDCl_3$ (H-19, δH = 2.40 ppm)

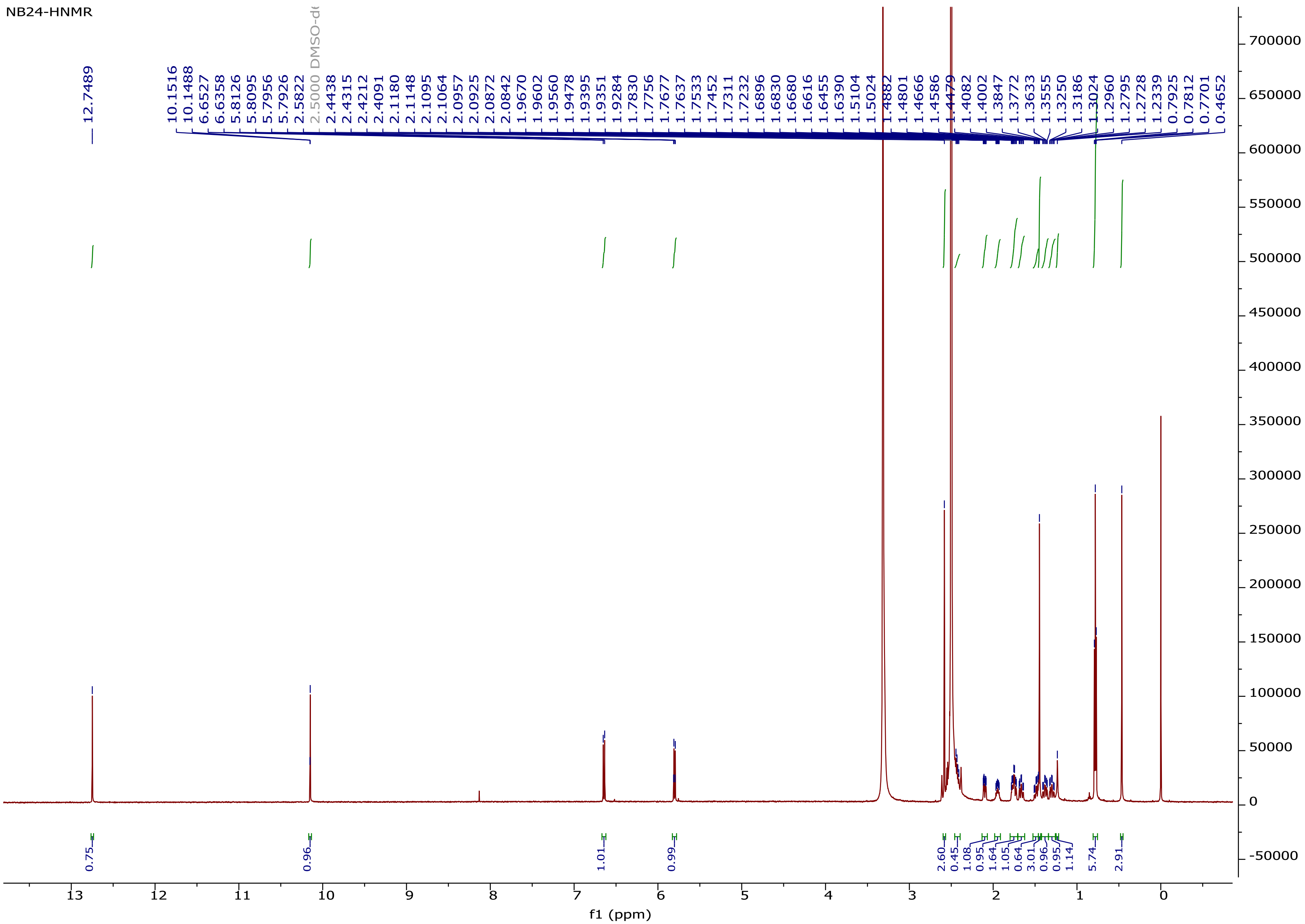

Figure S27. ^{1}H NMR Spectrum of **2** in DMSO.

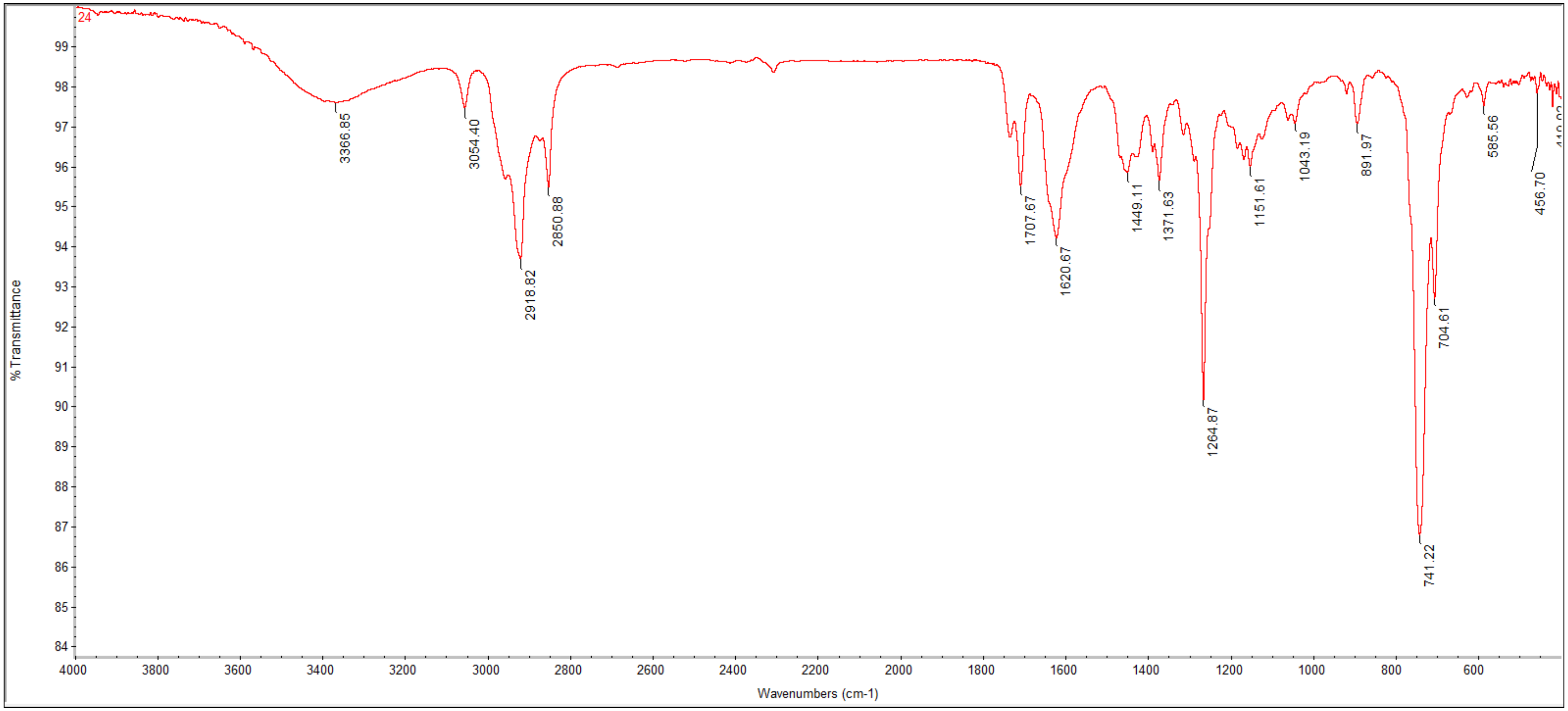

Figure S28. IR spectrum of compound **2**.

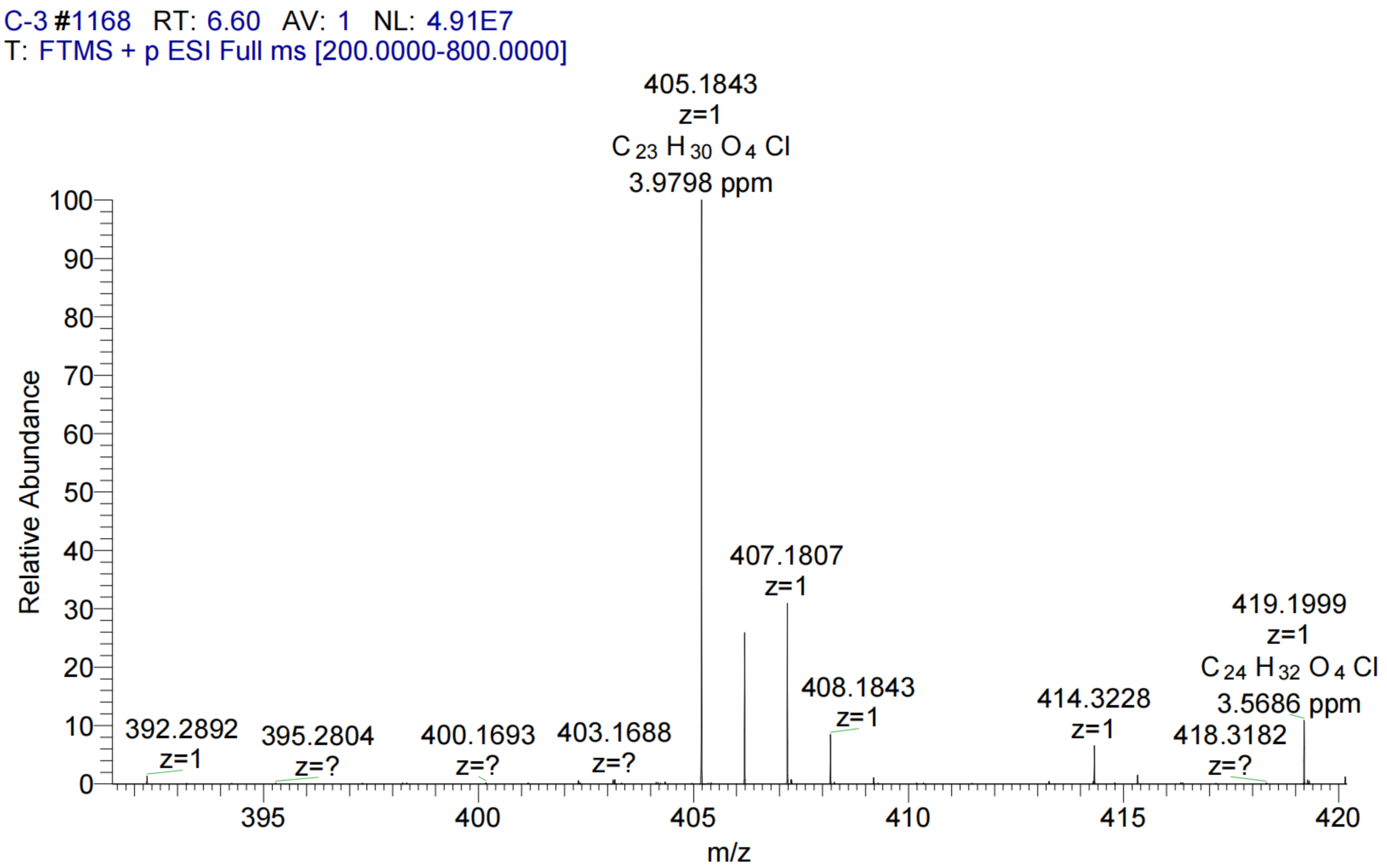

Figure S29. HR-ESIMS of compound **2**.

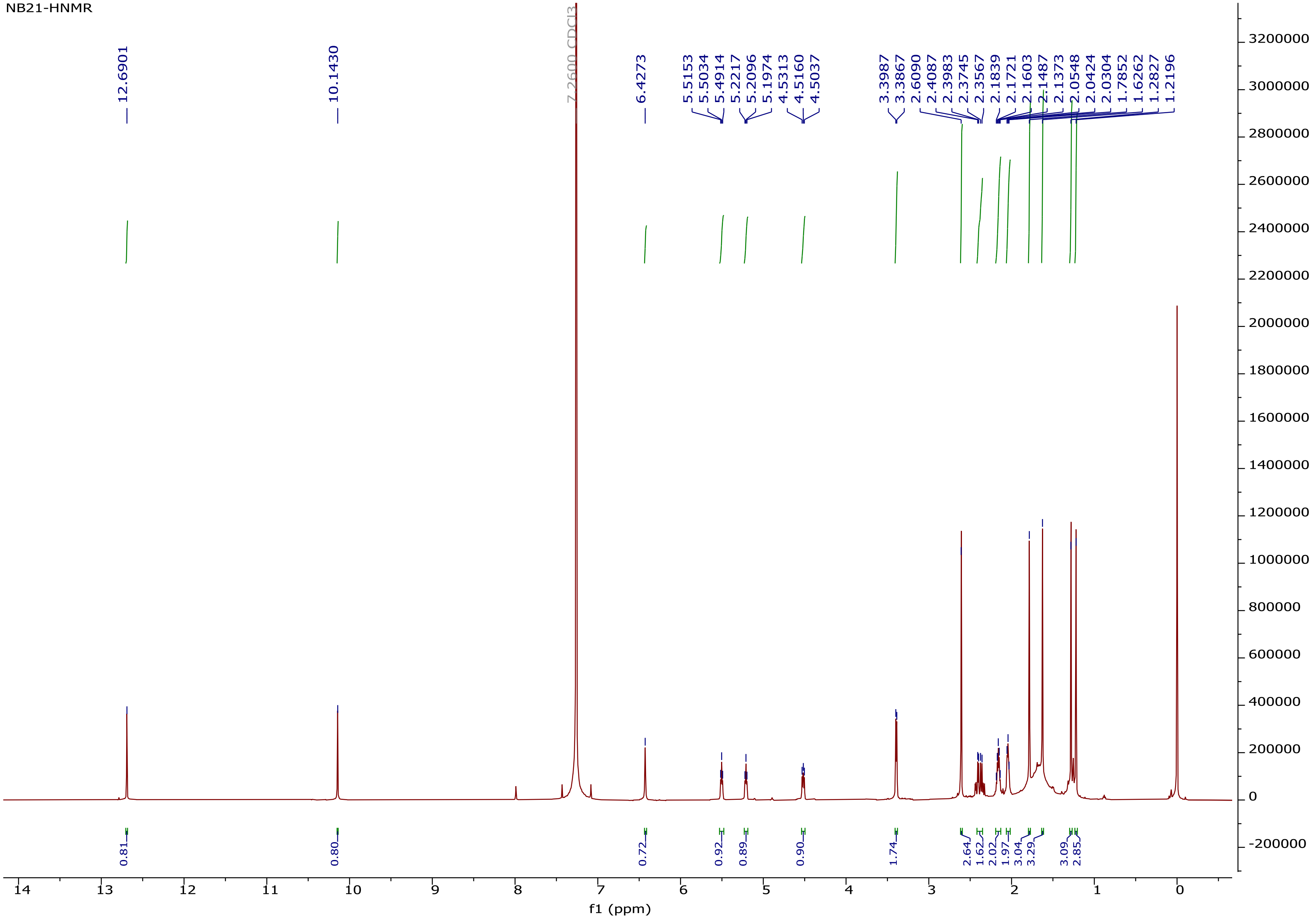

Figure S30. ^{1}H NMR Spectrum of **3** in $CDCl_3$.

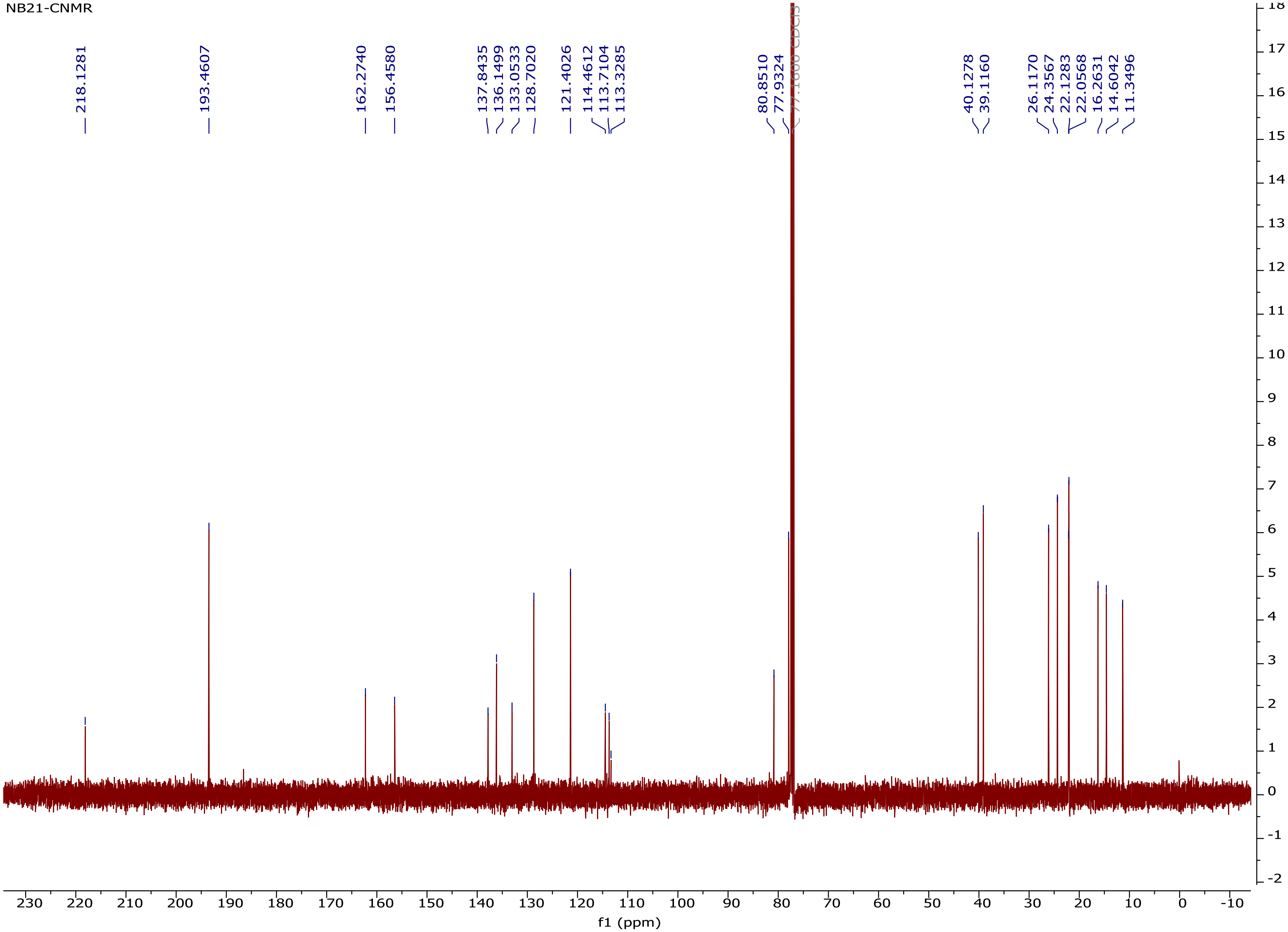

Figure S31. ^{13}C NMR Spectrum of **3** in $CDCl_3$.

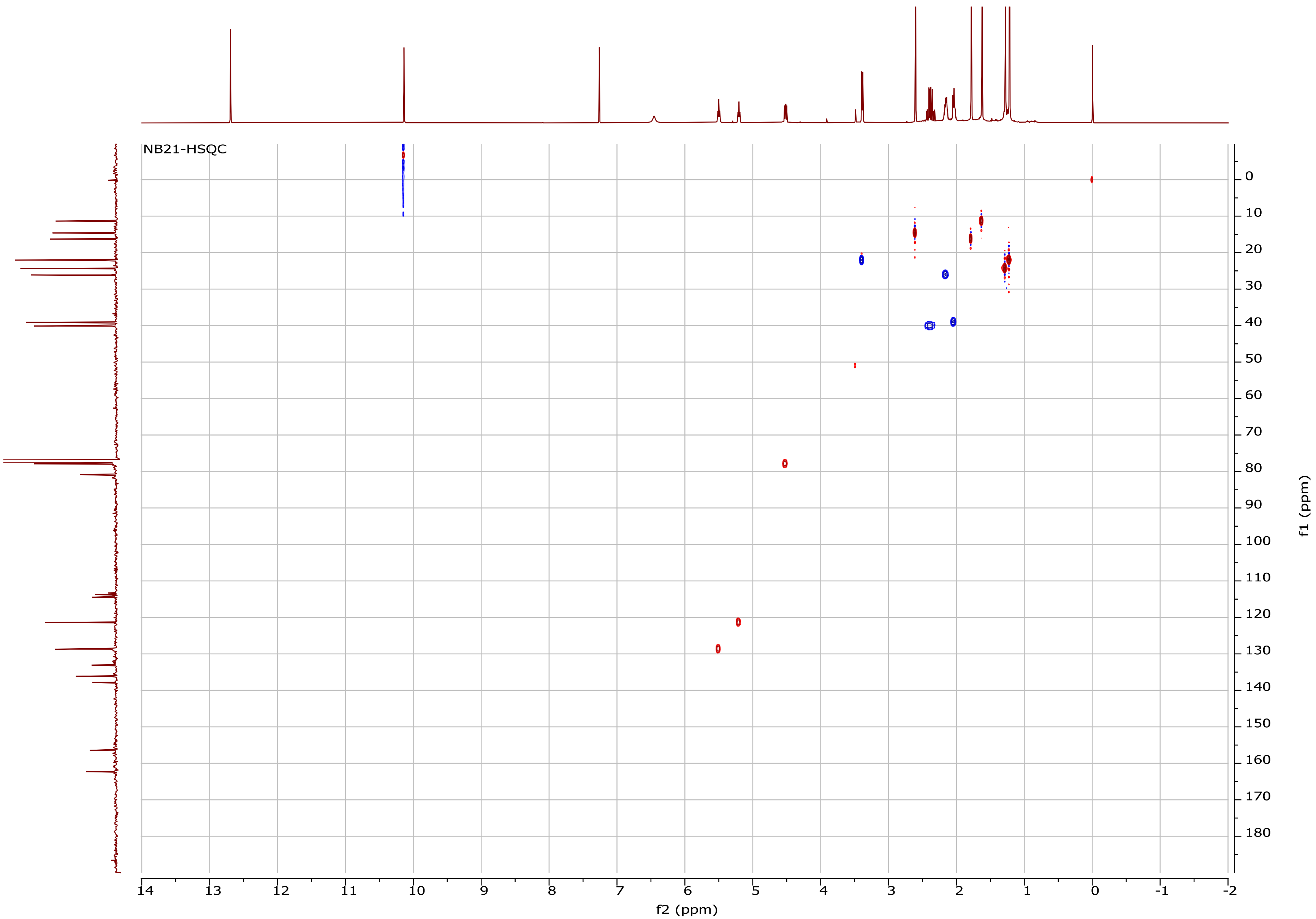

Figure S32. HSQC Spectrum of **3** in $CDCl_3$.

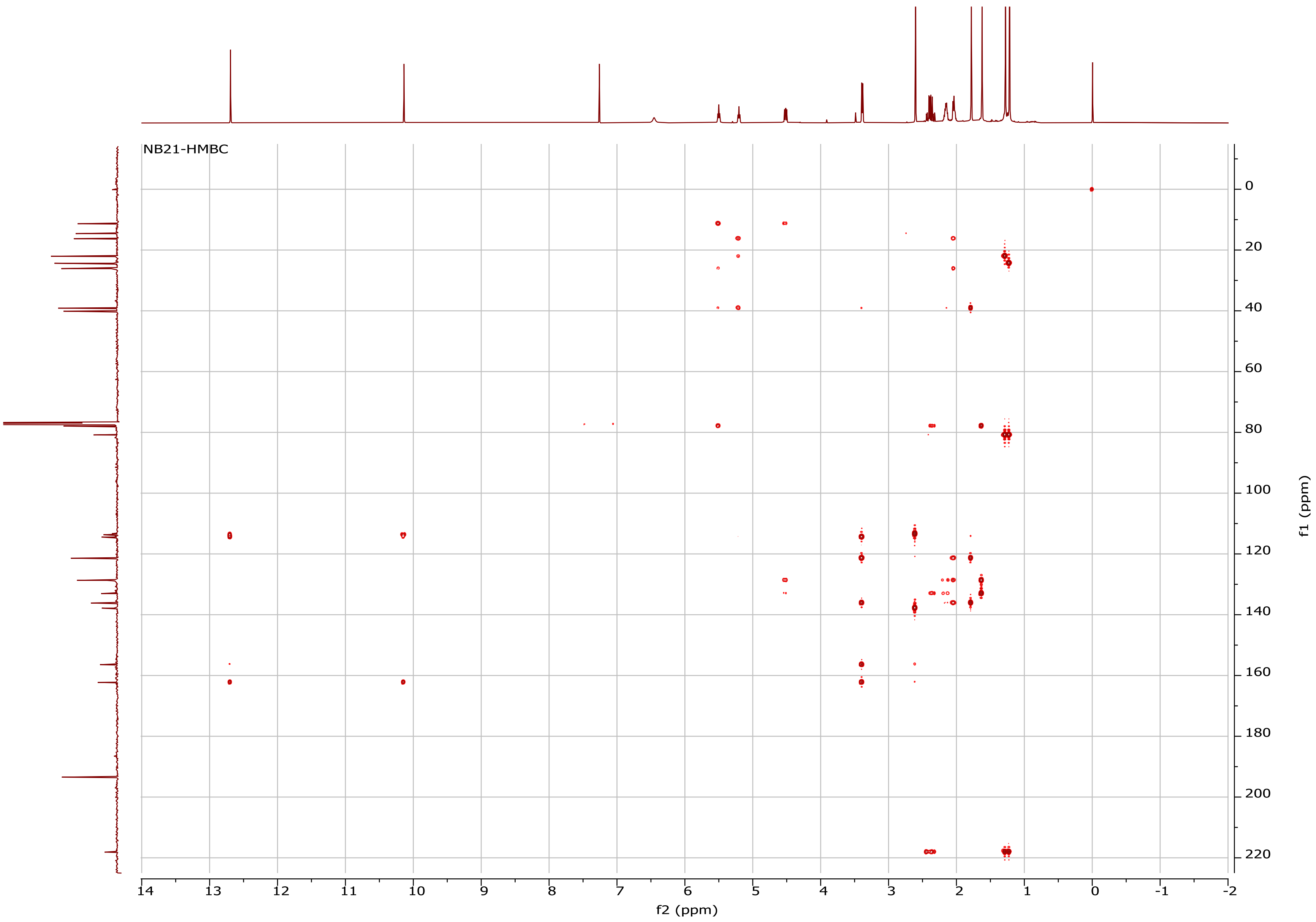

Figure S33. HMBC Spectrum of **3** in $CDCl_3$.

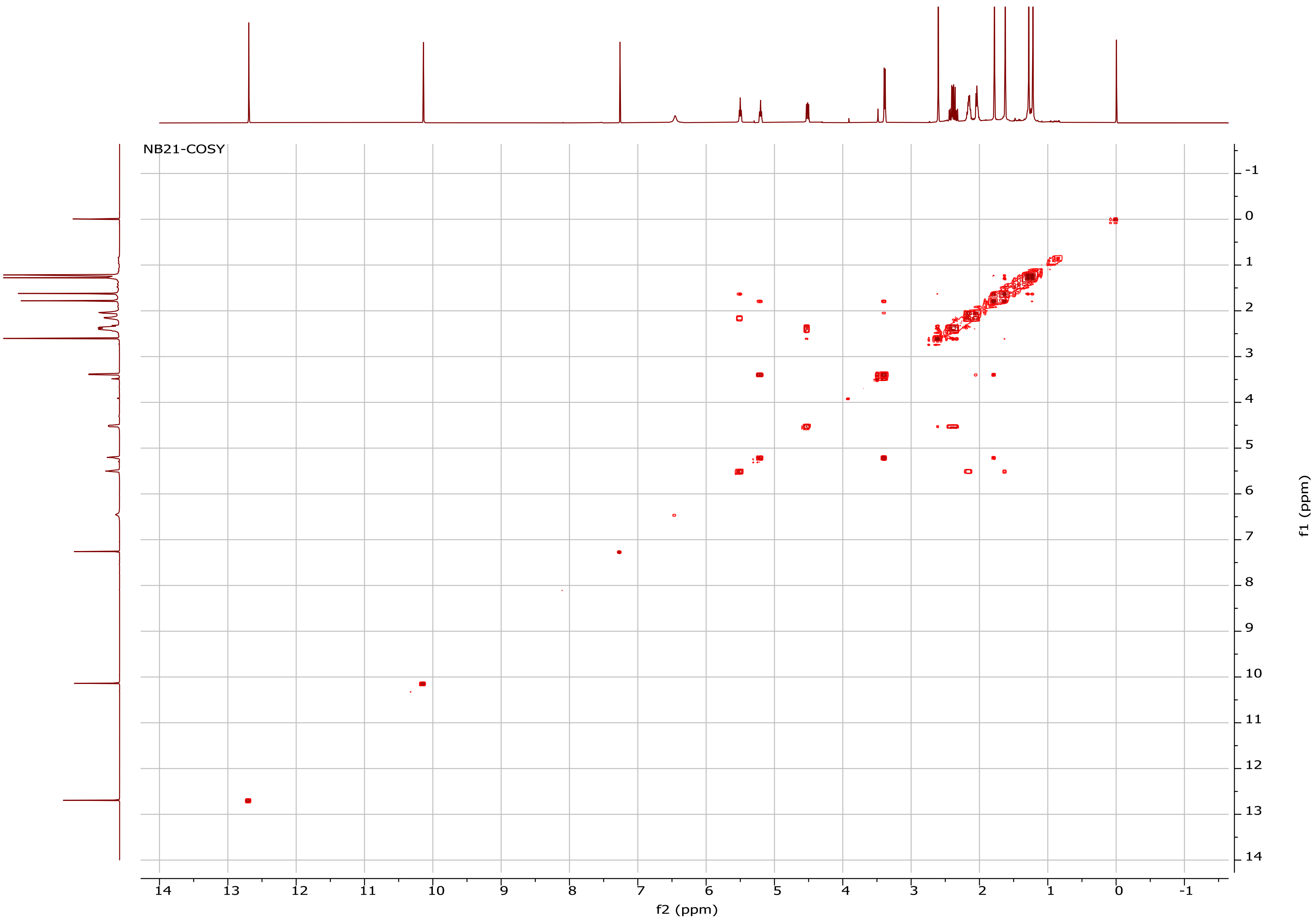

Figure S34. COSY Spectrum of **3** in $CDCl_3$.

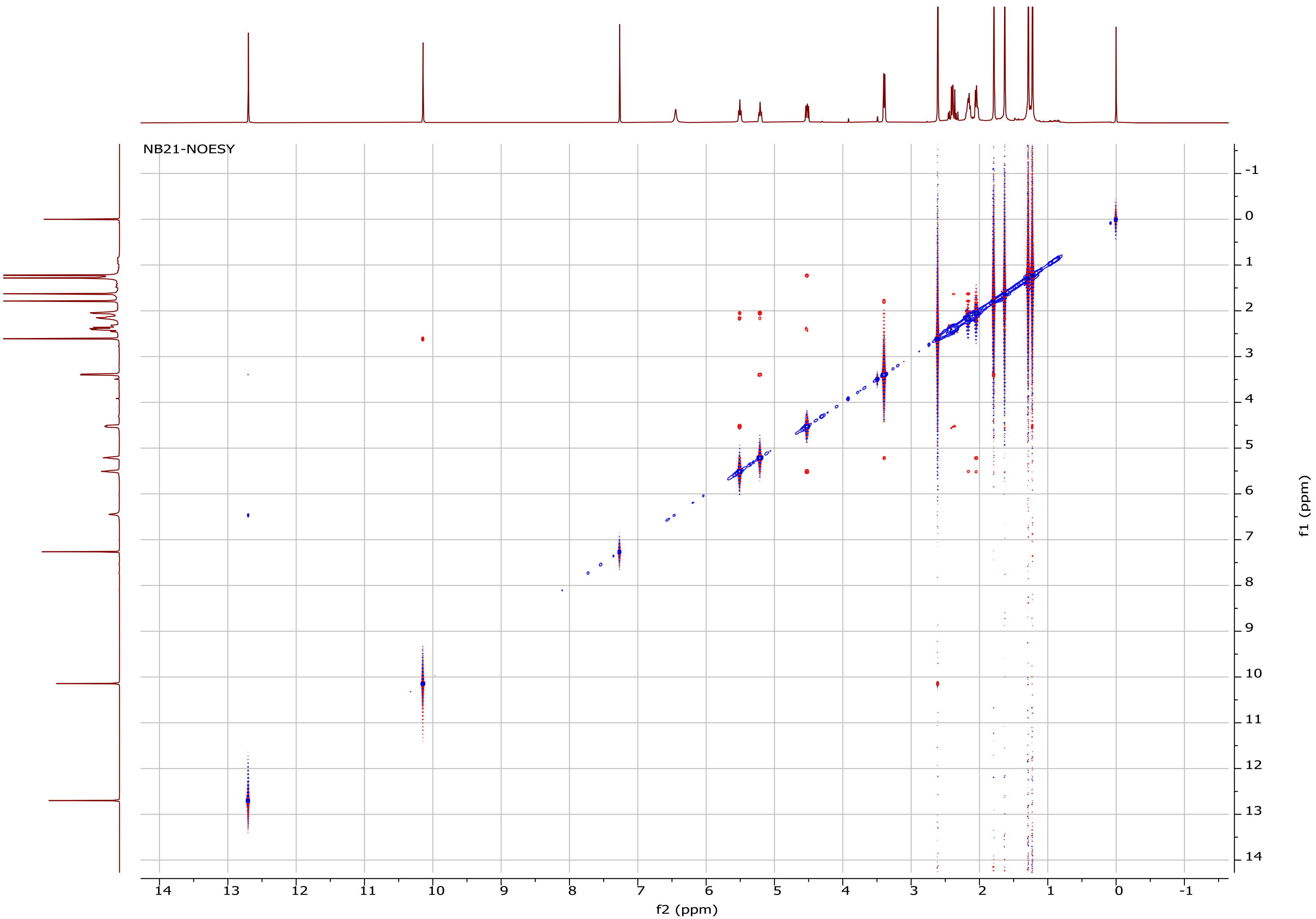

Figure S35. NOESY Spectrum of **3** in $CDCl_3$.

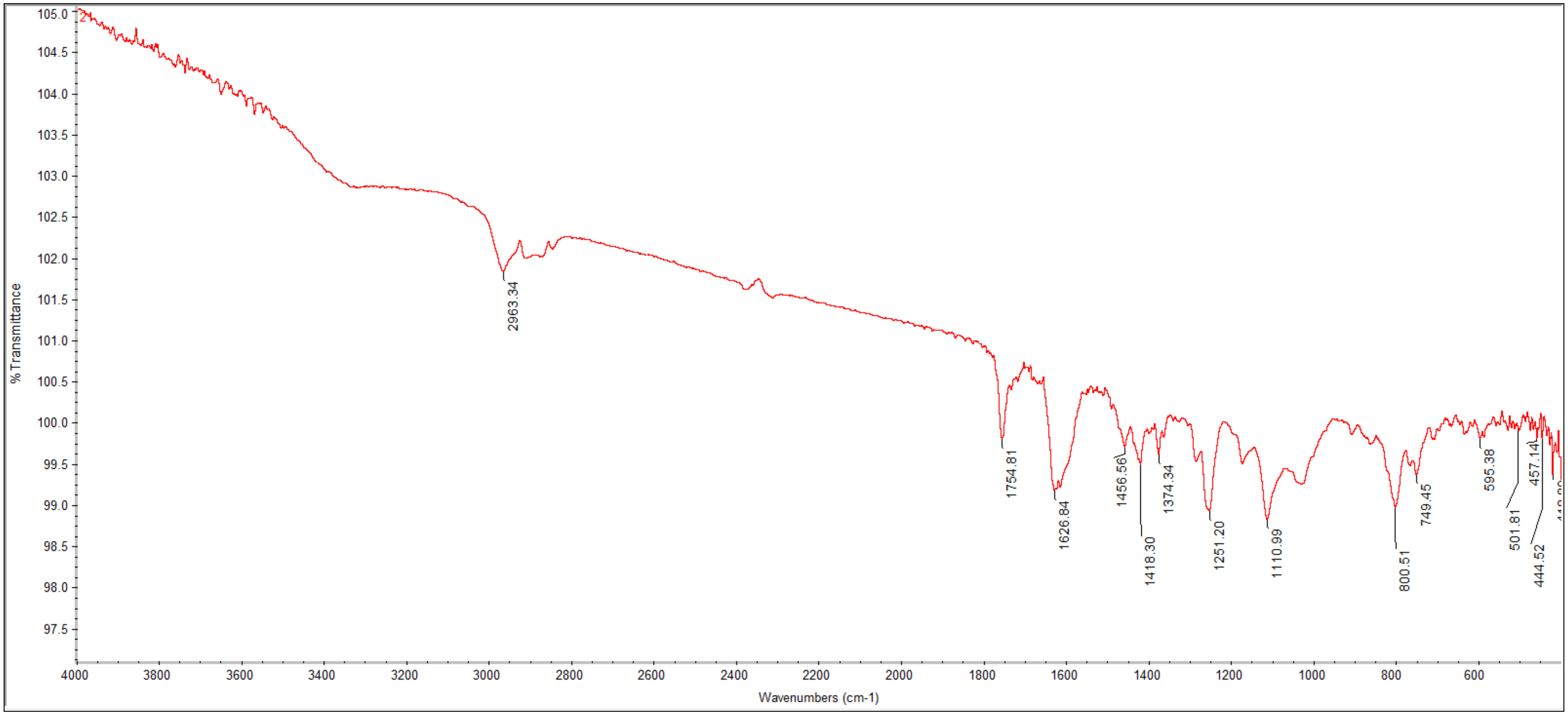

Figure S36. IR spectrum of compound **3**.

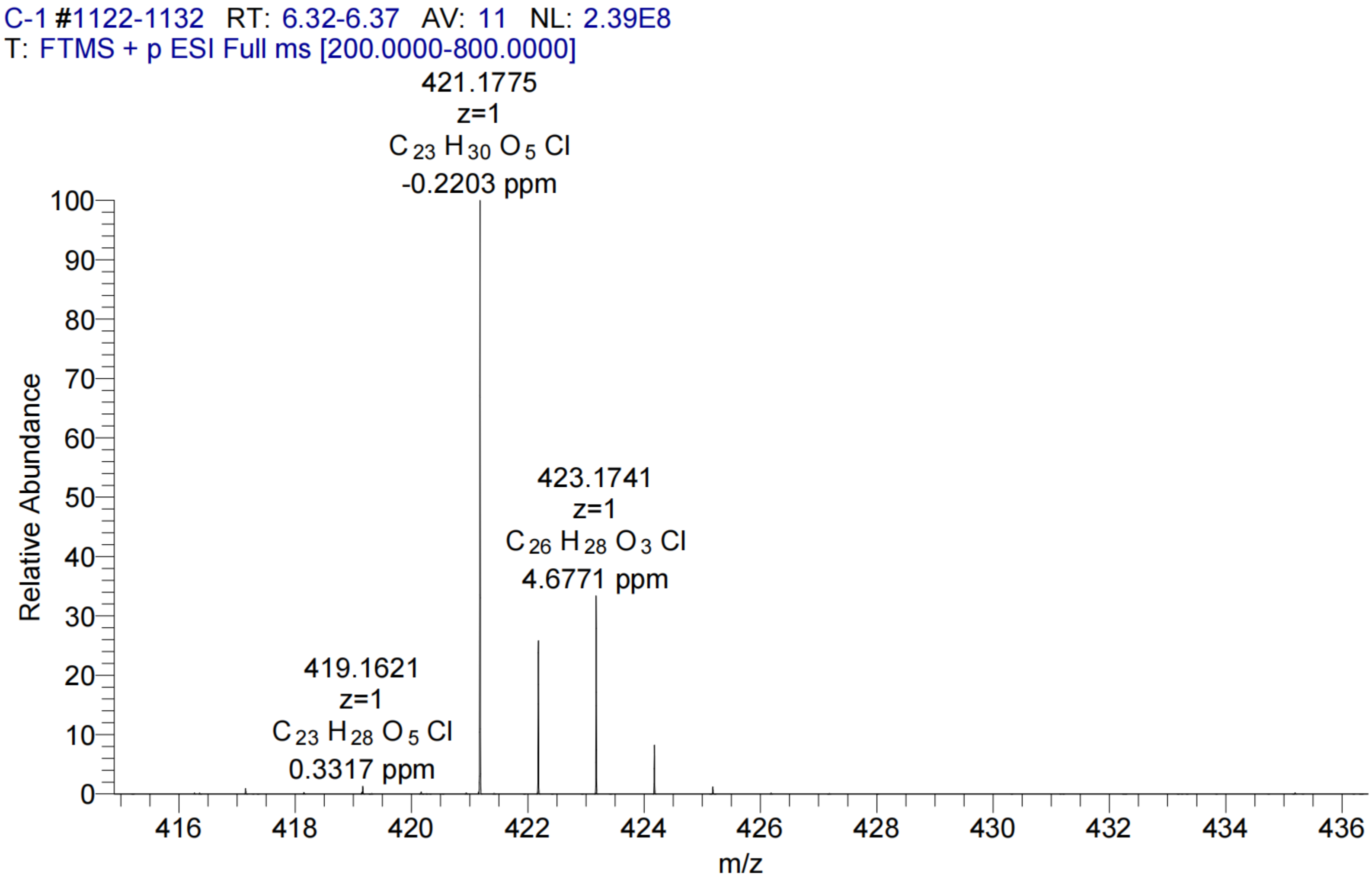

Figure S37. HR-ESIMS of compound **3**.

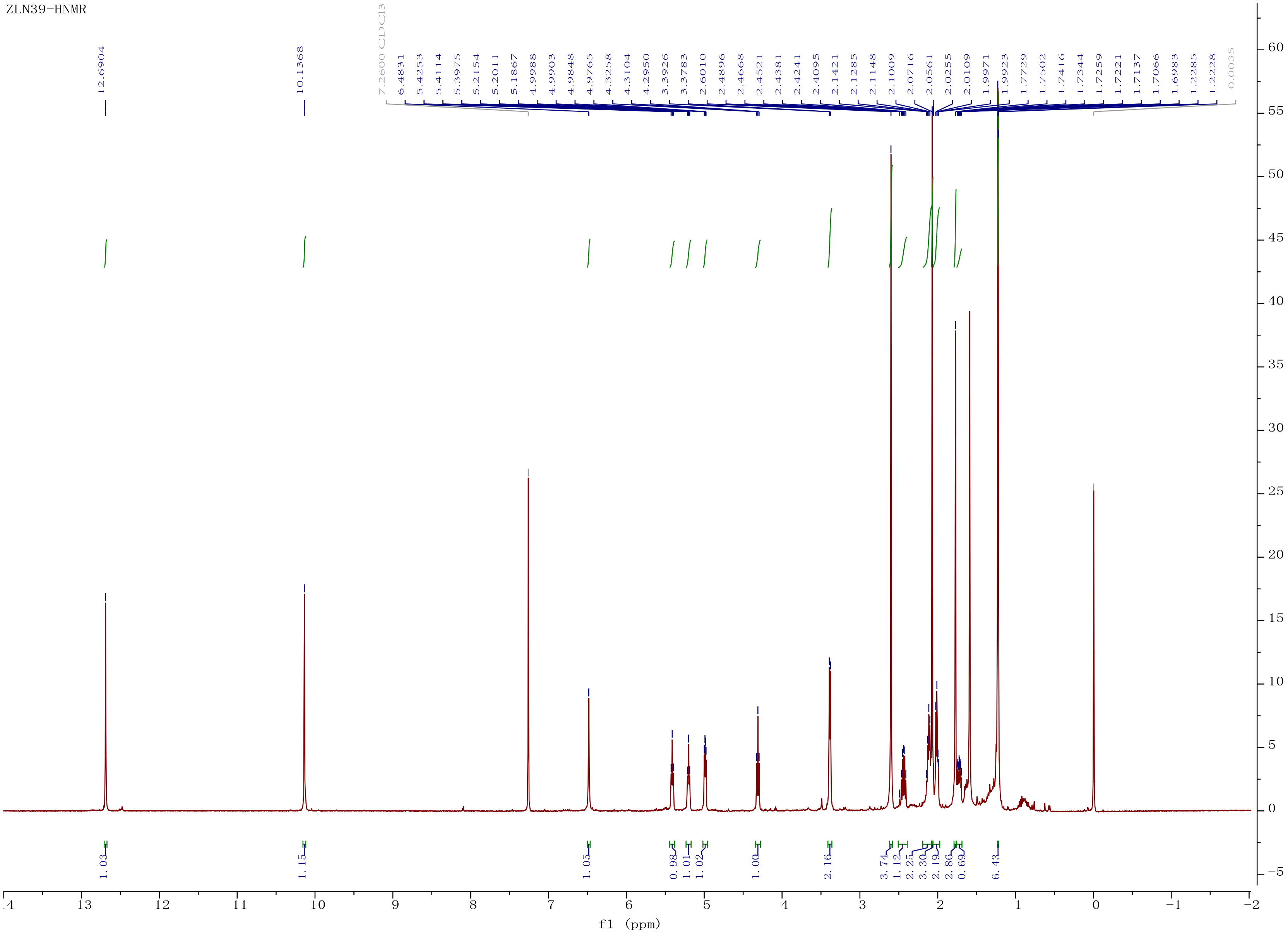

Figure S38. ^{1}H NMR Spectrum of **4** in CDCl₃.

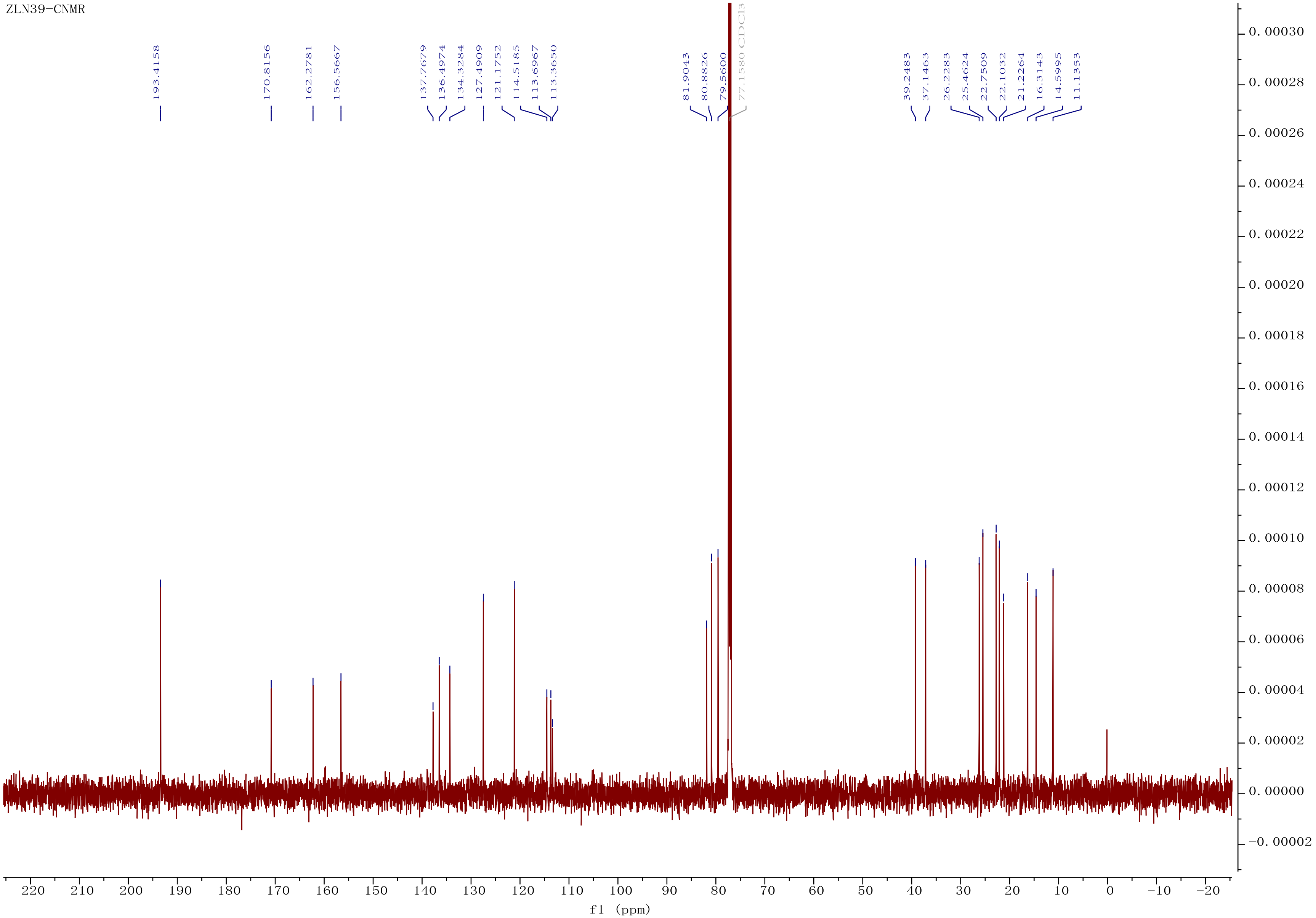

Figure S39. ^{13}C NMR Spectrum of **4** in $CDCl_3$.

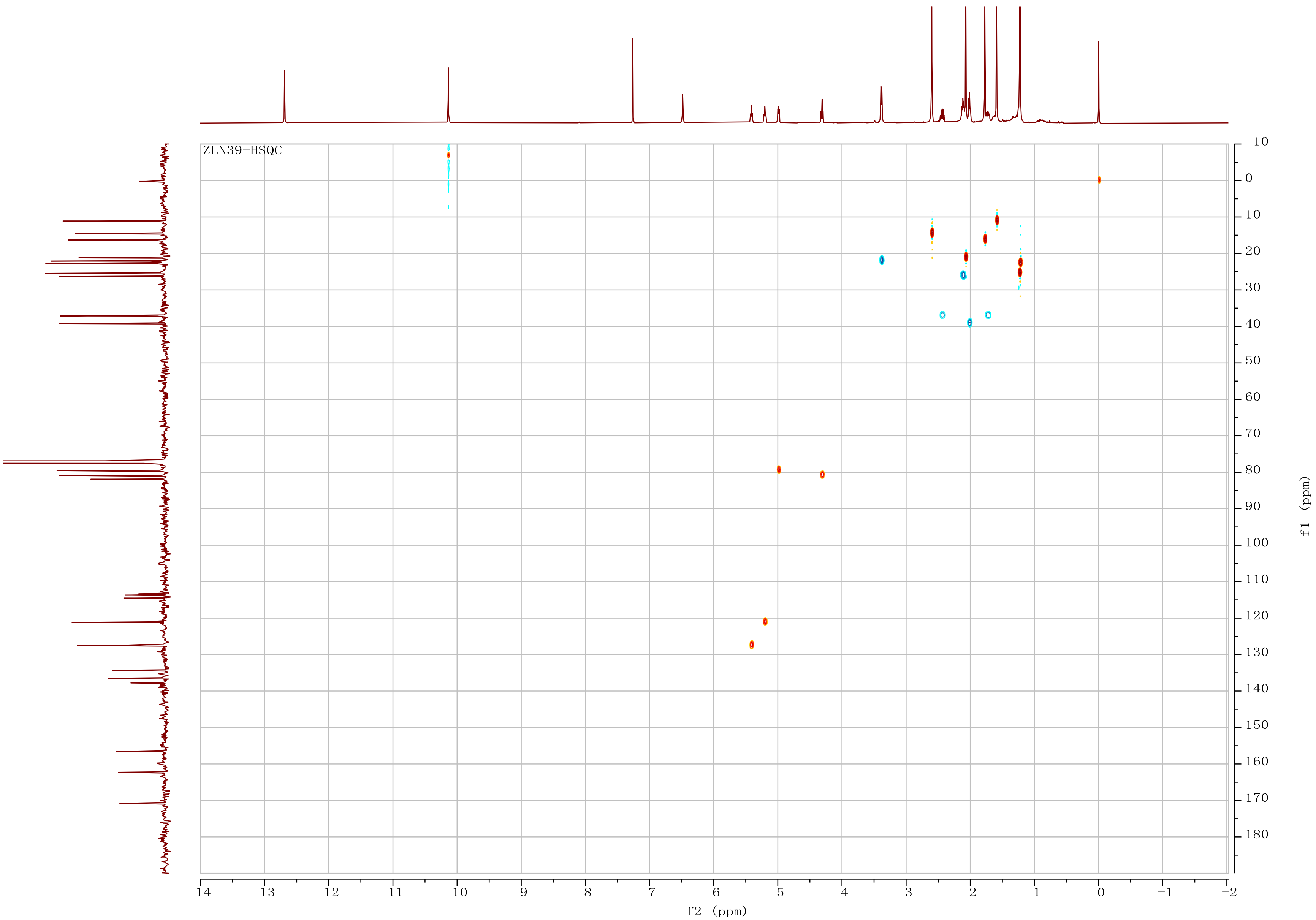

Figure S40. HSQC Spectrum of **4** in $CDCl_3$.

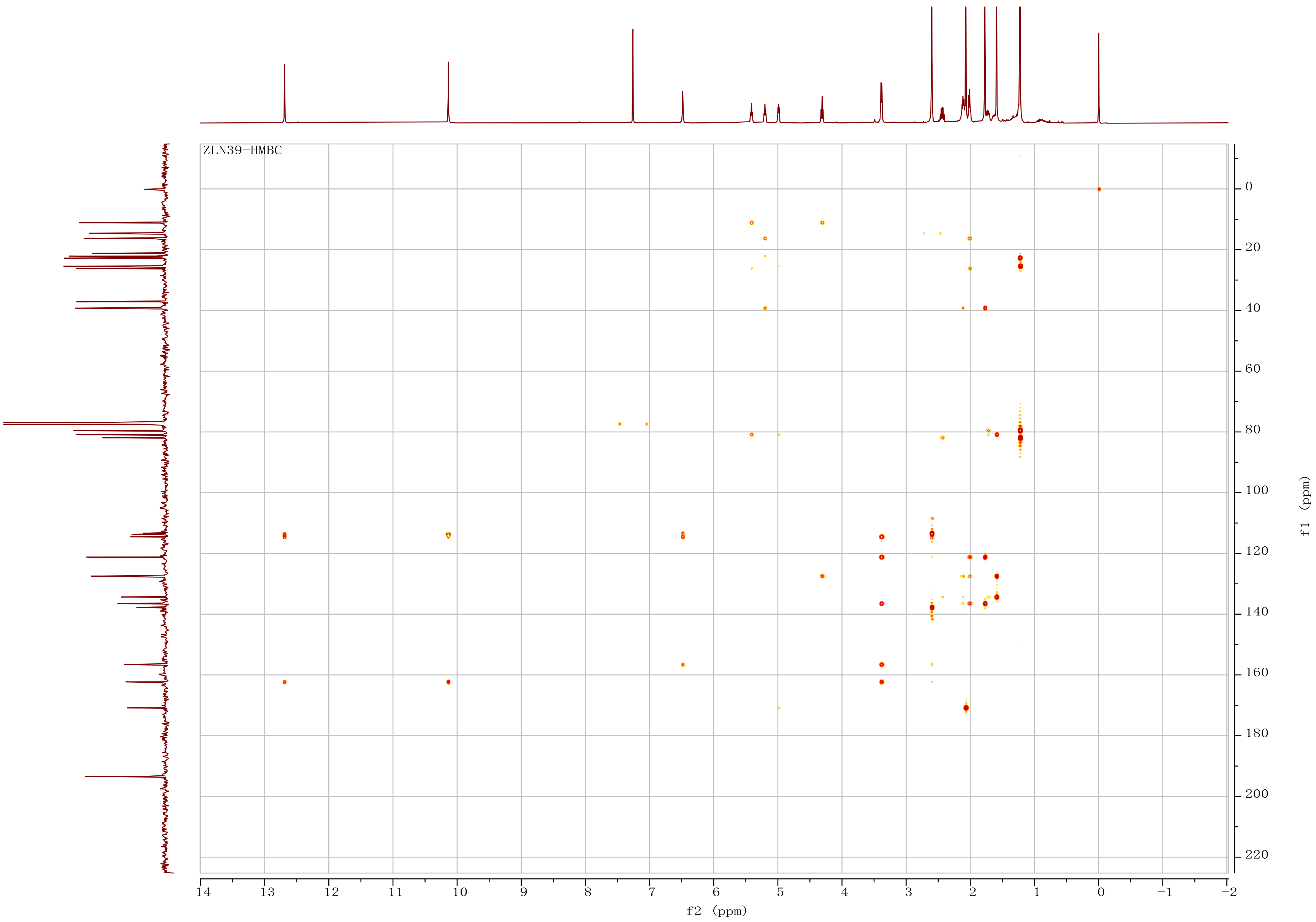

Figure S41. HMBC Spectrum of **4** in $CDCl_3$.

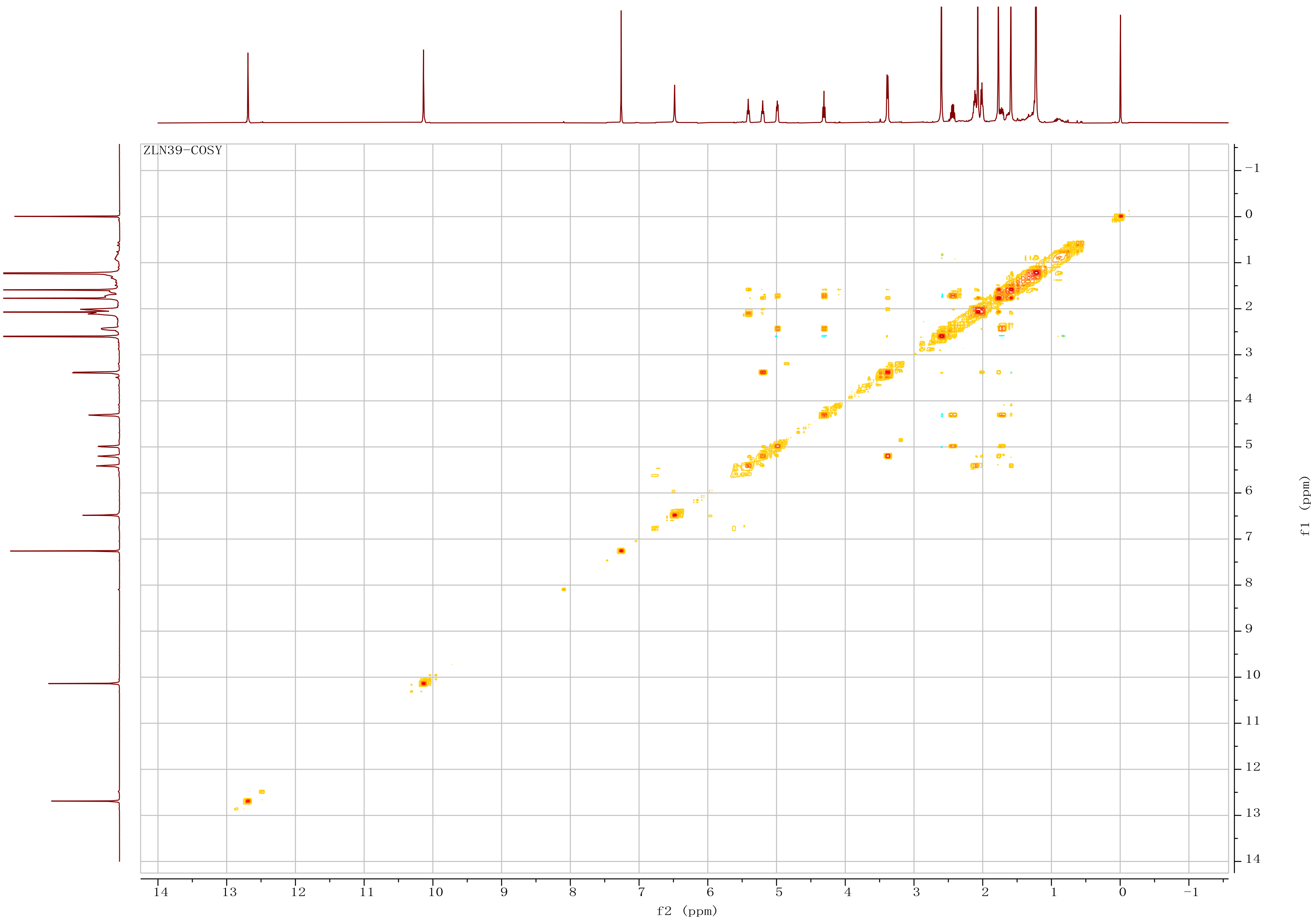

Figure S42. COSY Spectrum of **4** in $CDCl_3$.

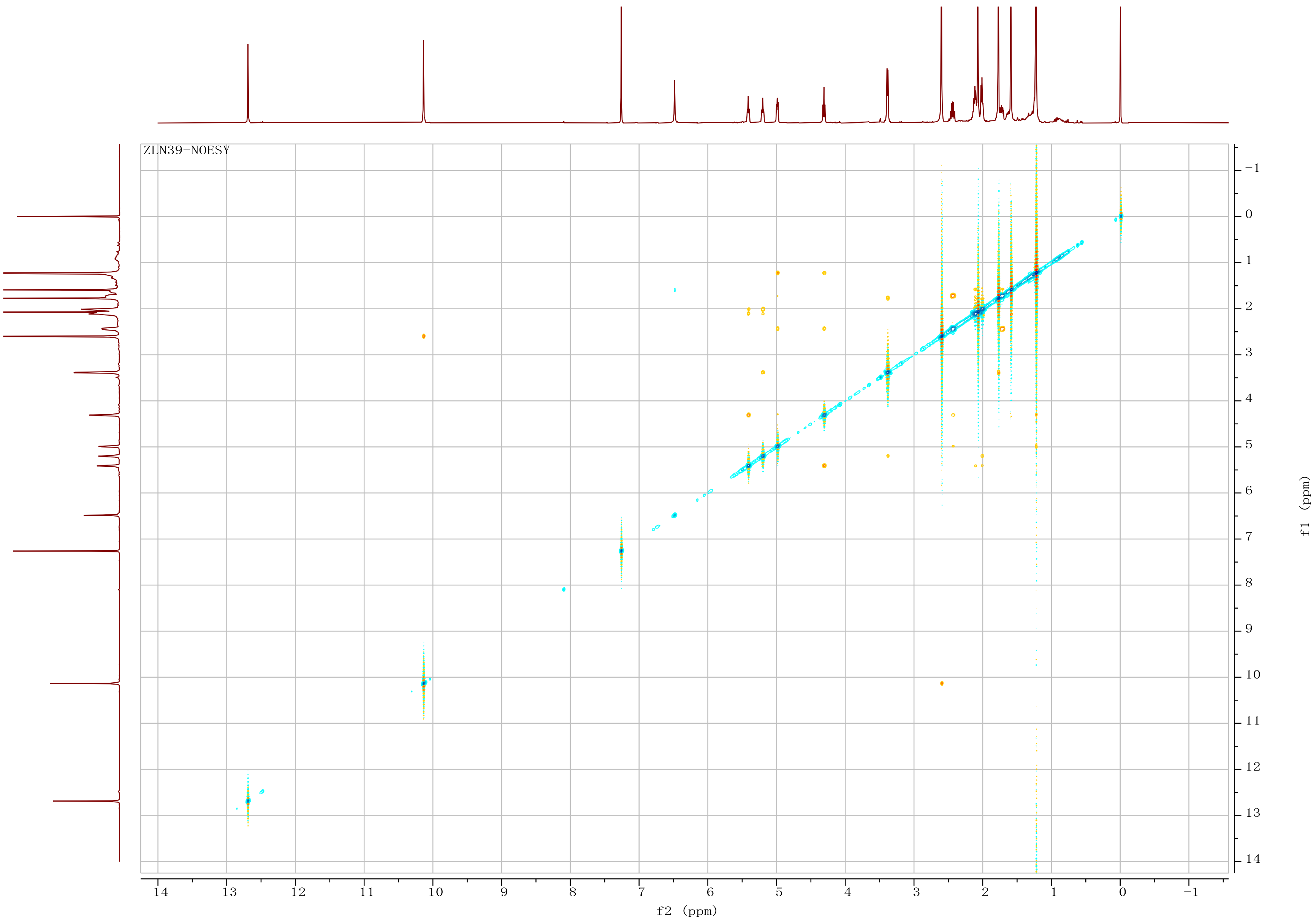

Figure S43. NOESY Spectrum of **4** in $CDCl_3$.

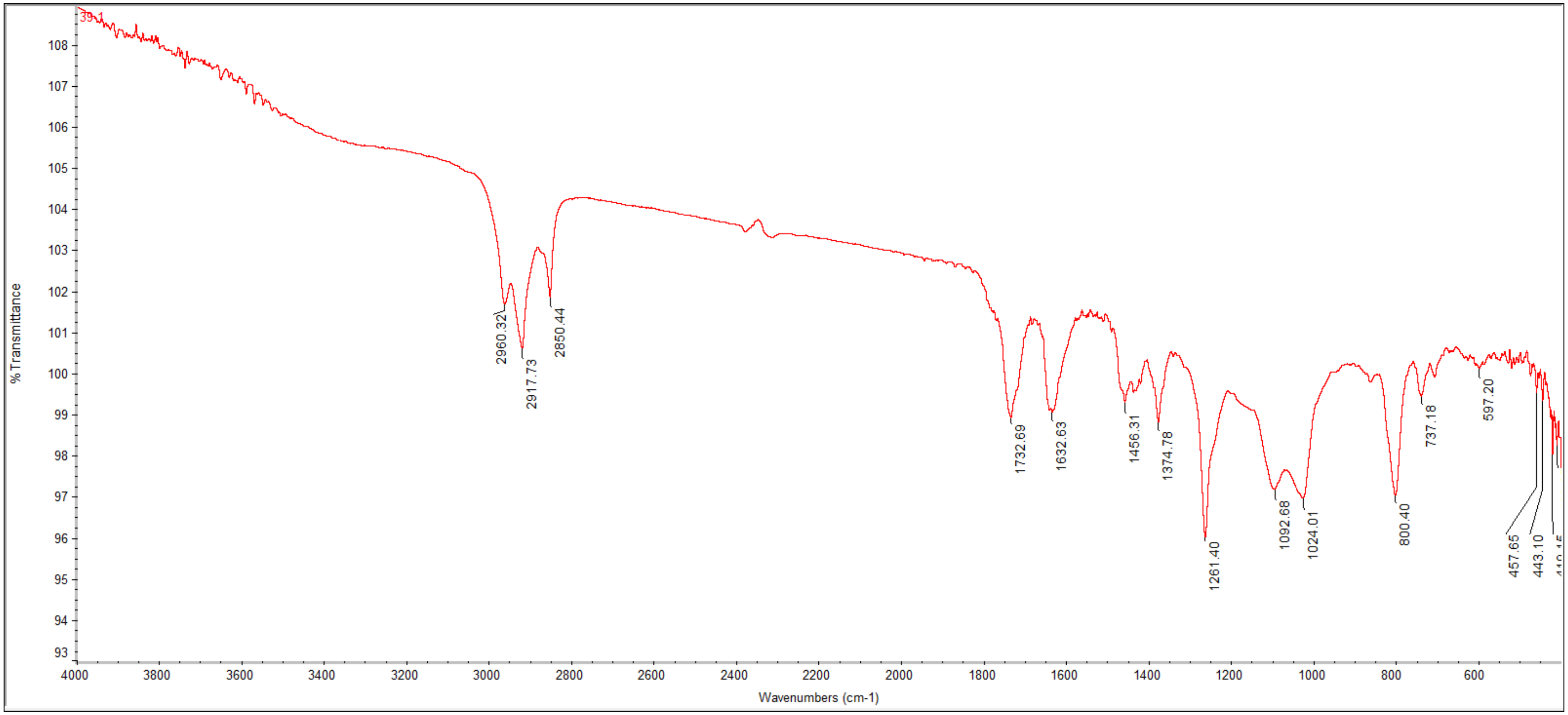

Figure S44. IR spectrum of compound **4**.

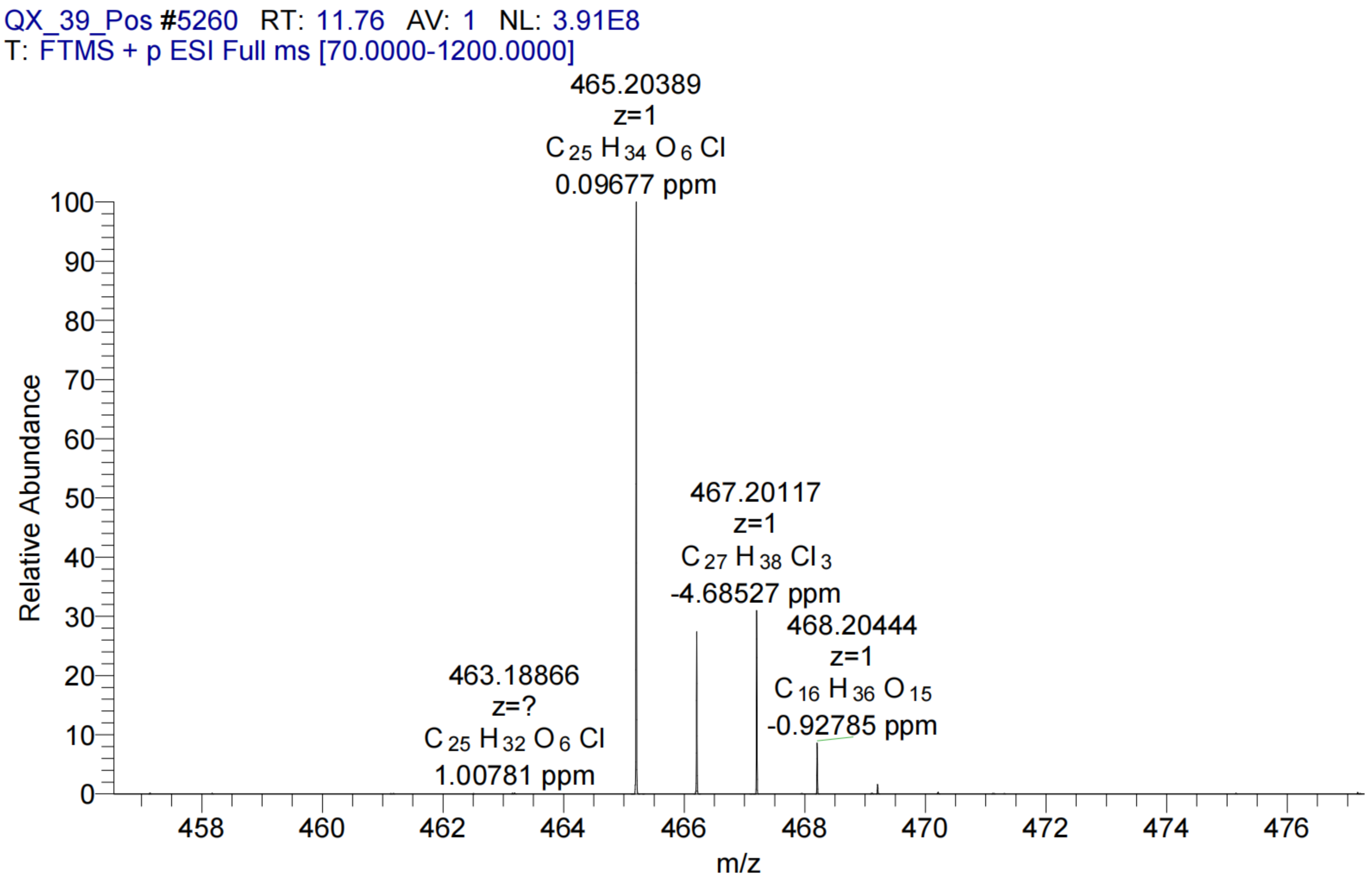

Figure S45. HR-ESIMS of compound **4**.

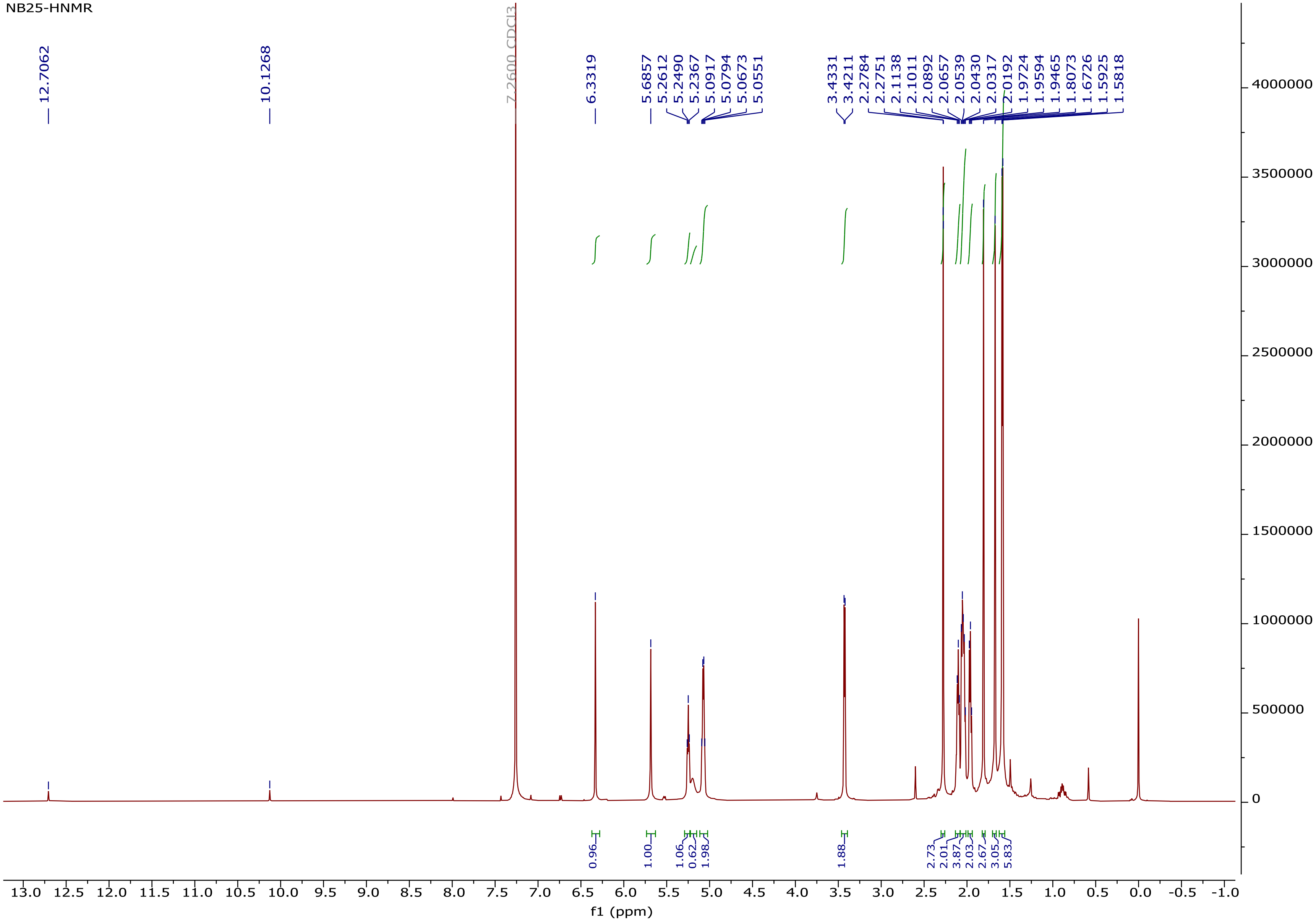

Figure S46. ^{1}H NMR Spectrum of **5** in $CDCl_3$.

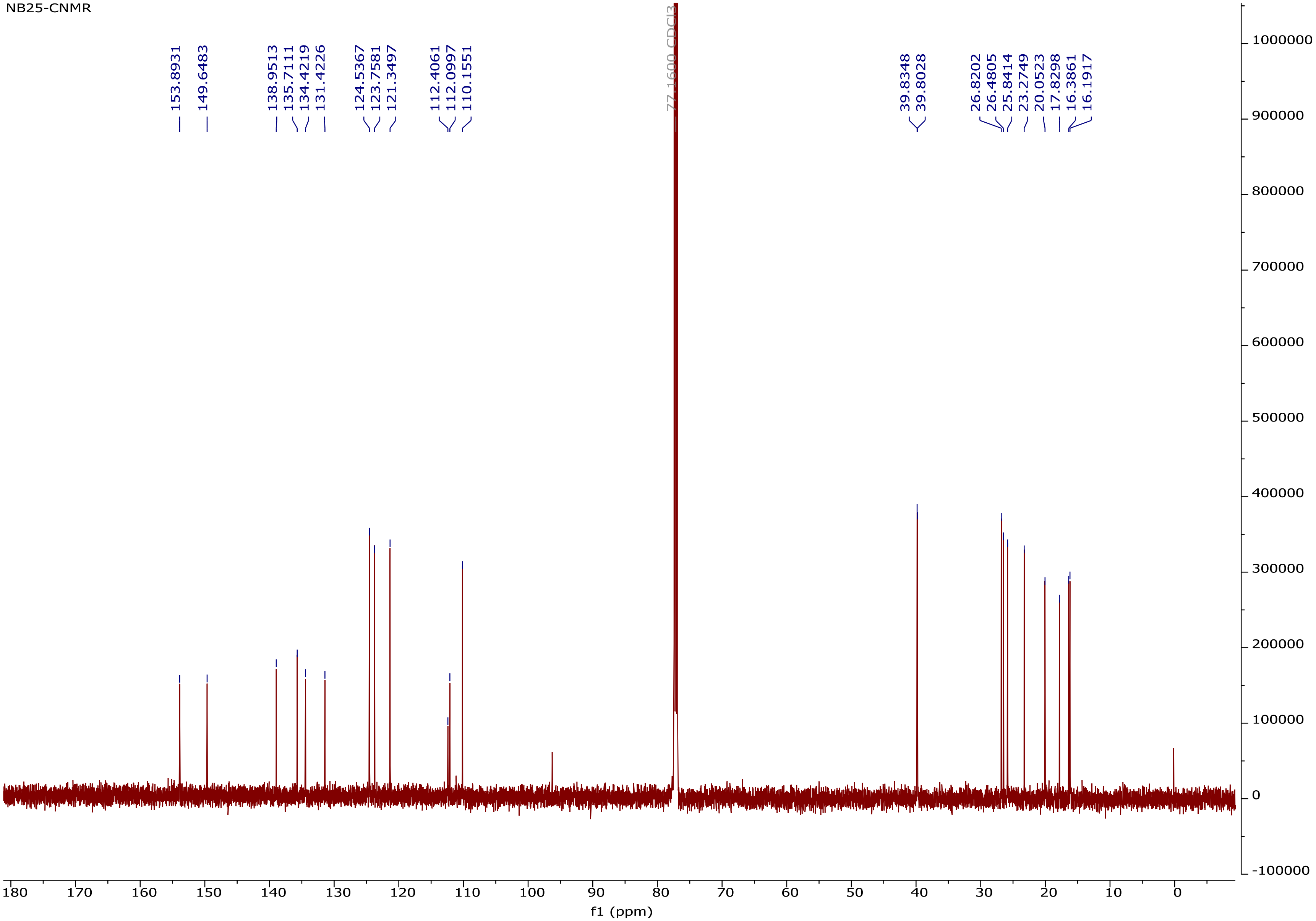

Figure S47. ^{13}C NMR Spectrum of **5** in $CDCl_3$.

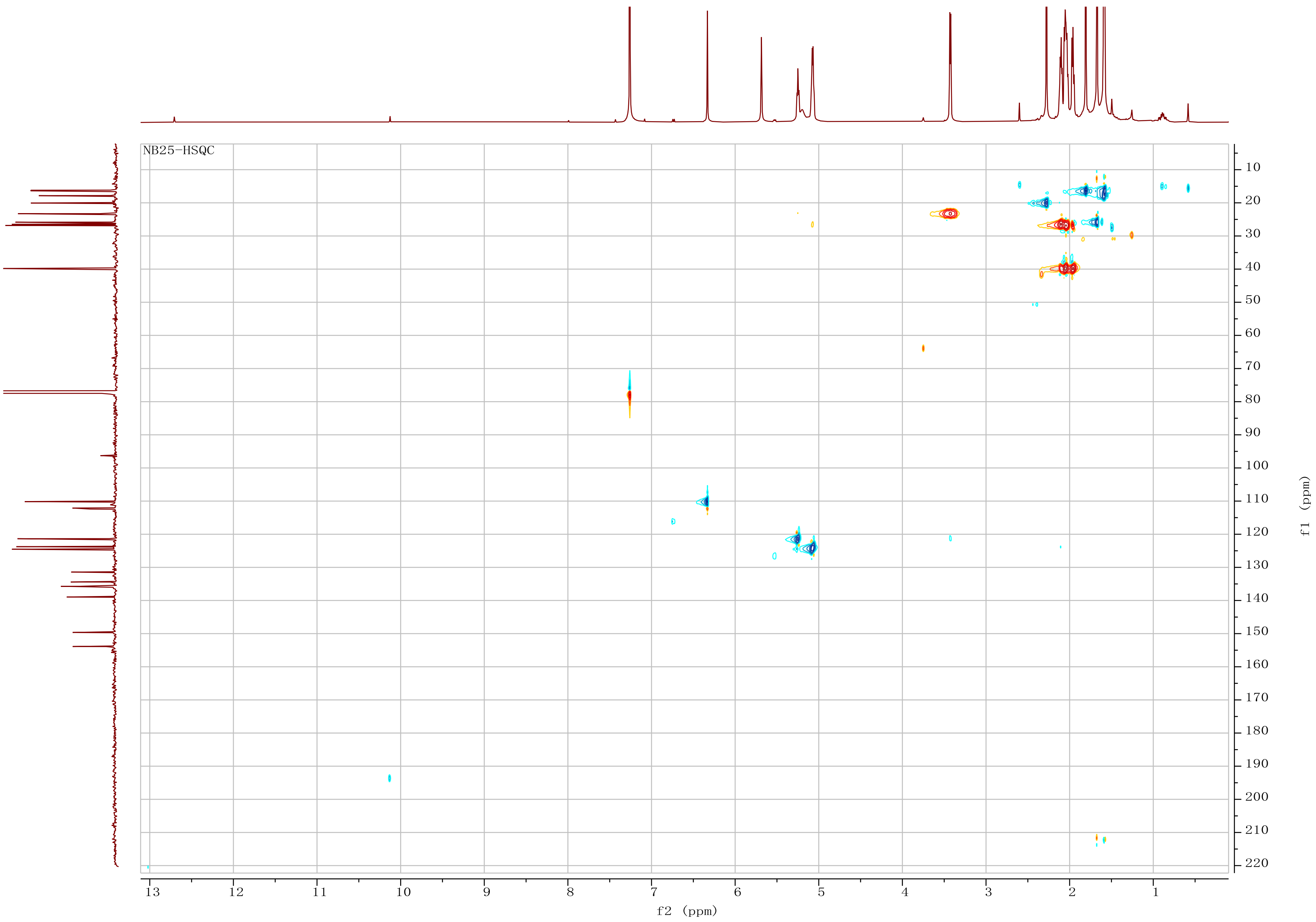

Figure S48. HSQC Spectrum of **5** in $CDCl_3$.

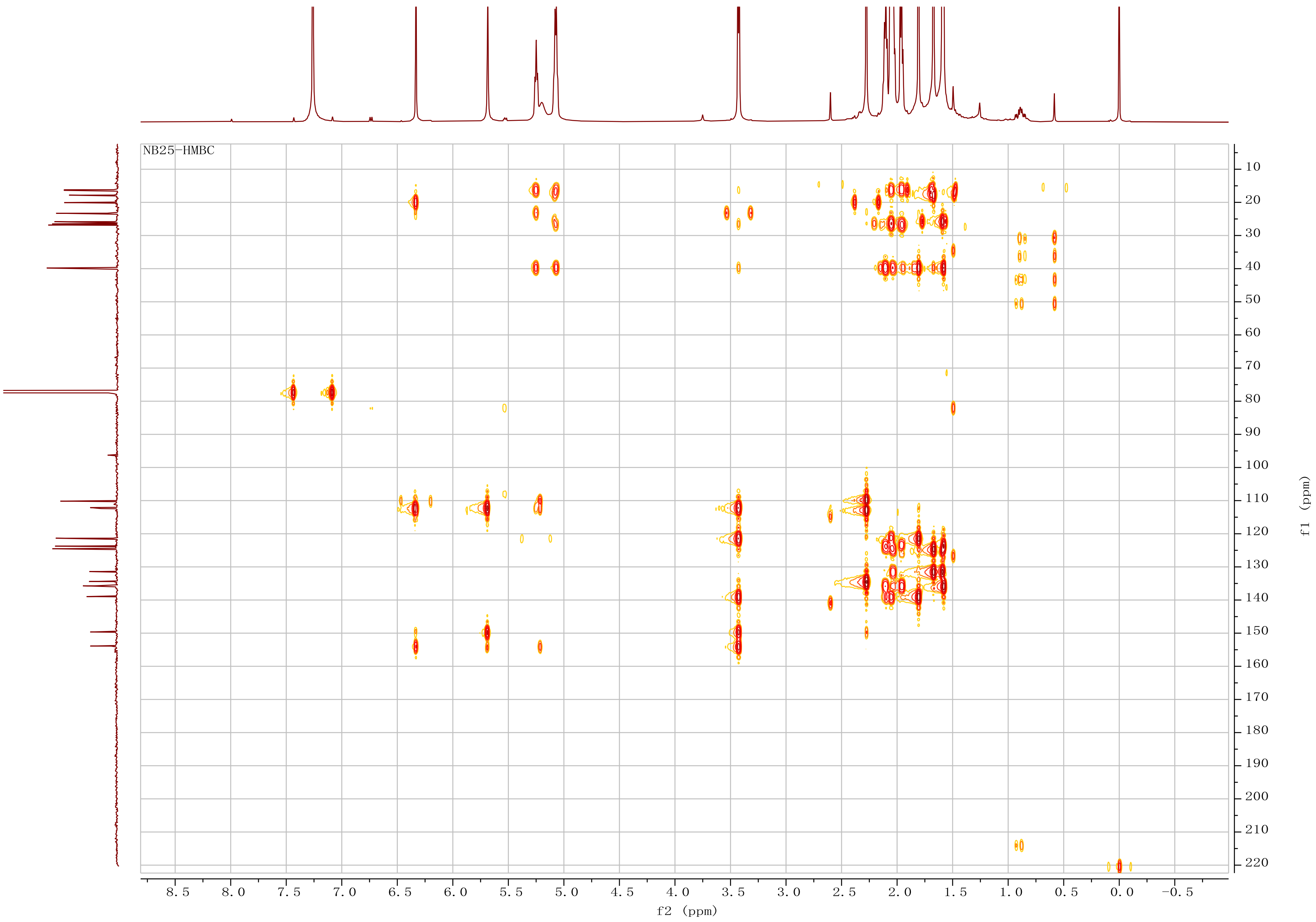

Figure S49. HMBC Spectrum of **5** in $CDCl_3$.

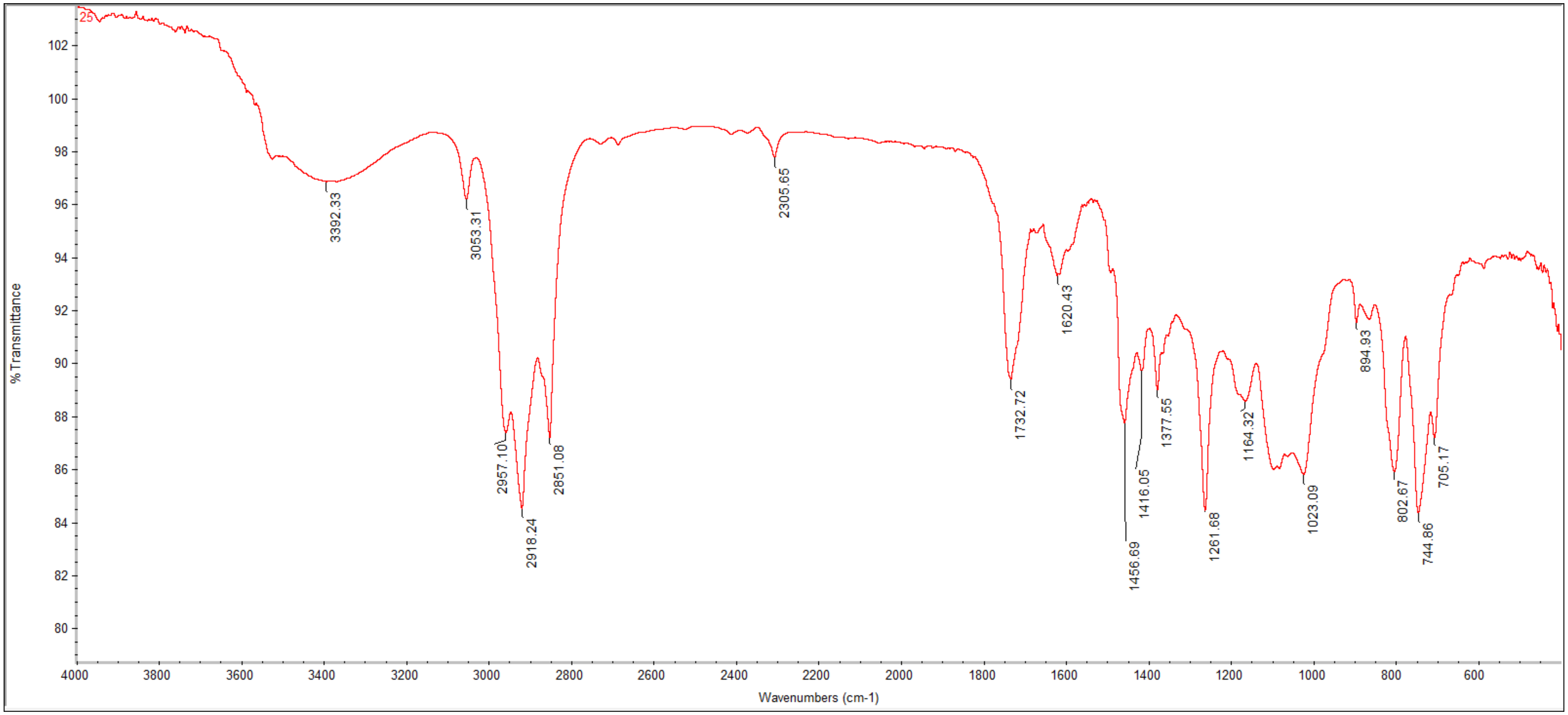

Figure S50. IR spectrum of compound **5**.

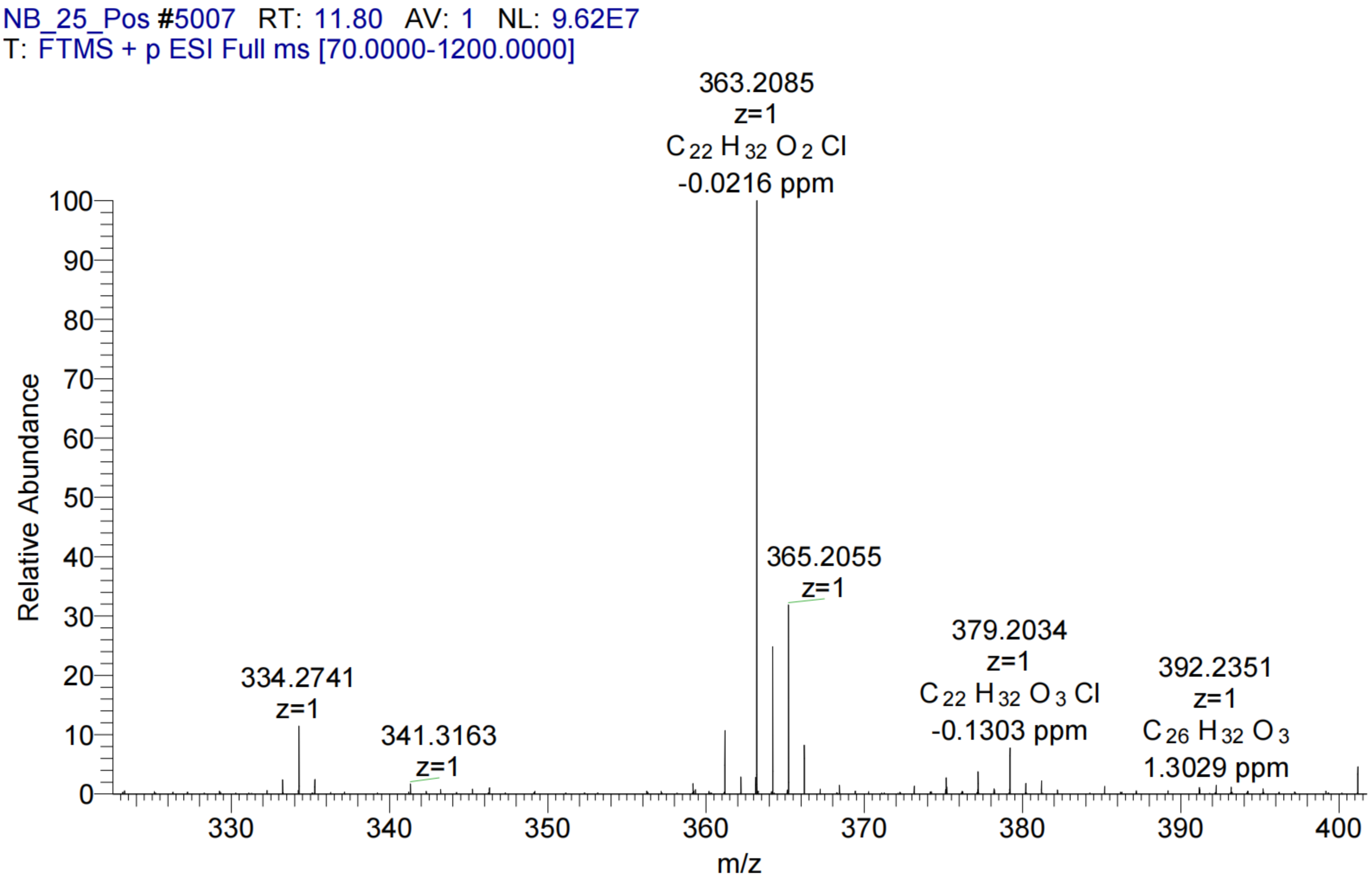

Figure S51. HR-ESIMS of compound **5**.

Table S1. Bacterial strains used in the antimicrobial screening assays.

Strains	Number	Closes sequence strain	Similarity
Staphylococcus aureus	MMBC-1011	*Staphylococcus aureus* ATCC29213	100%
Methicillin-resistant *Staphylococcus aureus*	MMBC-1010	*Staphylococcus aureus* strain 2-356MR	100%
Methicillin-resistant coagulase negative *staphylococci*	MMBC-1009	*Staphylococcus epidermidis* strain sESURV_p1_1200.	100%
Bacillus cereus	MMBC-1007	*Bacillus cereus* strain FDAARGOS_797	99.93%

Table S2. Cartesian coordinates of the low energy reoptimized conformers of **1** calculated at B3LYP/6 31+G(d) level of theory.

Conformer A		Standard Orientation (Ångstroms)		
I	Atom	X	Y	Z
1	C	3.87990100	-1.14121200	-0.47031800
2	C	4.62683400	0.06640800	-0.58604700
3	C	4.05770200	1.30543300	-0.15152900
4	C	2.77160800	1.28217700	0.37850100
5	C	2.04152400	0.07365300	0.50229100
6	C	2.58591700	-1.14494200	0.06848700
7	O	4.38617700	-2.31904500	-0.88232000
8	O	0.79609900	0.14550300	0.99619000
9	C	5.95379000	0.01863900	-1.15915600
10	H	6.50854900	0.96240600	-1.24228100
11	O	6.50645100	-1.02345300	-1.56746300
12	Cl	1.97338200	2.75197600	0.93467000
13	C	4.84753700	2.59041600	-0.26809600
14	C	1.75399300	-2.33986800	0.15129900
15	C	0.56859100	-2.29627900	0.77542700
16	C	0.07916200	-1.05564900	1.48133300
17	C	-1.40977300	-0.76491000	1.21051900
18	C	0.34879300	-1.15243000	2.99068200
19	C	-1.77232800	-0.64870200	-0.27679900
20	C	-3.24361400	-0.27585500	-0.62211700
21	C	-4.25128100	-1.30985300	-0.00432100
22	C	-5.71808400	-0.94117600	-0.29992600
23	C	-6.07775600	0.49076800	0.14098900
24	C	-5.05430900	1.49157000	-0.34991700
25	C	-3.60079300	1.15003700	-0.03358900
26	O	-5.37946200	2.50755100	-0.95601500
27	C	-2.65339500	2.29331900	-0.41564700
28	C	-3.34160800	-0.24647400	-2.16505800
29	C	-4.00237800	-2.77413900	-0.40675500
30	H	5.29813500	-2.13866600	-1.24236300
31	H	4.29849300	3.43211500	0.15150000
32	H	5.80306900	2.51912900	0.26262000

33	H	5.06663300	2.82662400	-1.31582600
34	H	2.11338500	-3.25085800	-0.31552300
35	H	-0.06793000	-3.17507000	0.83960200
36	H	-1.66080500	0.15673900	1.74901100
37	H	-1.97887100	-1.57111400	1.68869400
38	H	-0.17117900	-2.02174000	3.40828800
39	H	1.42026400	-1.27006500	3.18318100
40	H	-0.00833200	-0.25186900	3.50253800
41	H	-1.54148200	-1.59791500	-0.77540000
42	H	-1.11124000	0.08972200	-0.74279000
43	H	-4.12810300	-1.24994000	1.08754900
44	H	-5.92533100	-1.05611500	-1.37171600
45	H	-6.37880500	-1.64766100	0.21697100
46	H	-7.07172900	0.78641600	-0.20912900
47	H	-6.08101700	0.53694100	1.24040000
48	H	-3.57522100	1.03977800	1.06164500
49	H	-2.60994500	2.45007700	-1.49780300
50	H	-1.63866200	2.10491000	-0.05429100
51	H	-2.99812200	3.22976200	0.03472200
52	H	-2.61895800	0.45916600	-2.58821800
53	H	-3.11660700	-1.23064100	-2.58954200
54	H	-4.33376800	0.04943500	-2.52269000
55	H	-3.00098900	-3.12141200	-0.13315500
56	H	-4.13042200	-2.92967000	-1.48444100
57	H	-4.72282900	-3.42411400	0.10441100

Conformer B		Standard Orientation (Ångstroms)		
I	Atom	X	Y	Z
1	C	-3.59561400	0.56626200	-1.20993900
2	C	-4.20403600	-0.58076300	-0.62360200
3	C	-3.69927500	-1.11030200	0.60618800
4	C	-2.60700300	-0.47619400	1.19000500
5	C	-1.99343300	0.64960500	0.58669300
6	C	-2.49447900	1.19013800	-0.60752600
7	O	-4.06215400	1.09710600	-2.35605900
8	O	-0.95971600	1.21746200	1.22839800
9	C	-5.34393000	-1.18318000	-1.27923900
10	H	-5.79995400	-2.06233200	-0.80549200
11	O	-5.84378100	-0.76631900	-2.34422100
12	Cl	-1.92107300	-1.03099000	2.71563000
13	C	-4.34416000	-2.32546400	1.23542500
14	C	-1.88461800	2.41343400	-1.11632200
15	C	-0.75833400	2.88595500	-0.56372800
16	C	-0.03552800	2.14315800	0.53486600
17	C	1.11302200	1.30469700	-0.08172700
18	C	0.43018600	3.10400000	1.63102800
19	C	1.96433600	0.50727600	0.91845800
20	C	3.09222100	-0.39012500	0.32525400
21	C	2.51190100	-1.46037000	-0.66692800

22	C	3.61686000	-2.33122300	-1.29593400
23	C	4.71583700	-1.49989800	-1.98562000
24	C	5.21311800	-0.39244100	-1.08288700
25	C	4.12904000	0.49646500	-0.47885200
26	O	6.40767600	-0.22514000	-0.85841800
27	C	4.73292500	1.68407200	0.28022300
28	C	3.79632900	-1.06249000	1.52676300
29	C	1.42297800	-2.36914000	-0.07060100
30	H	-4.84148400	0.54492600	-2.64146800
31	H	-4.35217900	-3.17438600	0.54332500
32	H	-3.81158900	-2.63603800	2.13314900
33	H	-5.38251700	-2.11933000	1.52000300
34	H	-2.37800200	2.93545700	-1.92948100
35	H	-0.29854400	3.80692200	-0.91286000
36	H	1.73859700	2.00851200	-0.64615500
37	H	0.65877600	0.63761400	-0.82371000
38	H	1.20761600	3.76812500	1.23845700
39	H	-0.40709300	3.71780700	1.97778300
40	H	0.83761700	2.55874800	2.48650900
41	H	1.30452200	-0.12922500	1.51821500
42	H	2.43005100	1.19756000	1.62964000
43	H	2.04850300	-0.90196200	-1.49368400
44	H	4.07084500	-2.97385100	-0.53060300
45	H	3.16707400	-3.00593700	-2.03436700
46	H	5.56074200	-2.12171200	-2.29766700
47	H	4.29540000	-1.02690600	-2.88570300
48	H	3.57871700	0.89165900	-1.34702400
49	H	5.43578200	2.22065900	-0.36522700
50	H	5.28571700	1.36790000	1.17005400
51	H	3.96057200	2.39368700	0.59000700
52	H	4.18065000	-0.31095600	2.22437500
53	H	3.09710400	-1.69300800	2.08603100
54	H	4.64195500	-1.69087700	1.22680300
55	H	0.56821700	-1.80441900	0.31398400
56	H	1.81076700	-2.99070300	0.74507400
57	H	1.04411100	-3.04681000	-0.84526400

Conformer C		Standard Orientation (Ångstroms)		
I	Atom	X	Y	Z
1	C	-3.30752000	1.18213000	0.76431900
2	C	-4.12303600	0.96772900	-0.38394900
3	C	-3.94460300	-0.20681600	-1.18170600
4	C	-2.95540000	-1.10921200	-0.80266100
5	C	-2.13462600	-0.87642900	0.32811900
6	C	-2.31610600	0.26116600	1.13003800
7	O	-3.46576700	2.26894000	1.54283800
8	O	-1.22197400	-1.80876200	0.64692000
9	C	-5.13592200	1.94521900	-0.71761300
10	H	-5.75286800	1.75864200	-1.60629800

11	O	-5.35433800	2.98333300	-0.06026500
12	Cl	-2.65885100	-2.59291400	-1.70665900
13	C	-4.81004800	-0.43956700	-2.40046100
14	C	-1.51460500	0.37762100	2.34286700
15	C	-0.48501400	-0.45561200	2.55418900
16	C	-0.06991300	-1.47481400	1.51945700
17	C	1.02961300	-0.93630400	0.56966800
18	C	0.30418600	-2.81157500	2.16016100
19	C	2.34772200	-0.55756000	1.26501400
20	C	3.46319000	0.05623000	0.36659800
21	C	3.84933100	-0.91944900	-0.80196900
22	C	4.92687700	-0.32048800	-1.72634900
23	C	4.53426500	1.05975600	-2.28727300
24	C	4.04920300	1.98266200	-1.19064800
25	C	2.96272200	1.40937100	-0.28418500
26	O	4.49887900	3.11533800	-1.04973800
27	C	2.42128400	2.46499200	0.68711000
28	C	4.67081900	0.33352600	1.29177500
29	C	4.28403400	-2.32552400	-0.35271200
30	H	-4.20762700	2.80754200	1.15124400
31	H	-4.71164200	0.38085300	-3.11988100
32	H	-4.53532500	-1.36136600	-2.91110700
33	H	-5.86831900	-0.51481200	-2.12578700
34	H	-1.79232200	1.13003200	3.07371400
35	H	0.09945100	-0.40729700	3.46857500
36	H	0.61127700	-0.06336800	0.05381000
37	H	1.19963400	-1.70754900	-0.19075200
38	H	1.15108000	-2.68414700	2.84090500
39	H	-0.53930600	-3.20678700	2.73484800
40	H	0.58179700	-3.54097100	1.39205700
41	H	2.76918800	-1.44377500	1.75271100
42	H	2.13921000	0.15000300	2.07535800
43	H	2.94504000	-1.04286000	-1.41636600
44	H	5.88072600	-0.23935200	-1.18927100
45	H	5.10461300	-1.00570400	-2.56400500
46	H	5.36458800	1.53366100	-2.82006300
47	H	3.70547600	0.93363400	-2.99996000
48	H	2.14905200	1.13192300	-0.97221700
49	H	2.11156900	3.35703500	0.13324700
50	H	3.17341900	2.78064000	1.41665700
51	H	1.54832400	2.09375000	1.23142900
52	H	4.38536600	0.99175300	2.11907900
53	H	5.04619700	-0.59534200	1.73410700
54	H	5.50559000	0.81190700	0.76826100
55	H	3.51096900	-2.83932000	0.22755100
56	H	5.19811500	-2.29778500	0.25189700

Table S3. Cartesian coordinates of the low energy reoptimized conformers of **2** calculated at B3LYP/6 31+G(d) level of theory.

Conformer A		Standard Orientation (Ångstroms)		
I	Atom	X	Y	Z
1	C	-3.85042800	-1.30145700	-0.33766500
2	C	-4.67999500	-0.16891200	-0.57981200
3	C	-4.19411200	1.14811300	-0.30195100
4	C	-2.90691300	1.27387000	0.21057800
5	C	-2.09571500	0.14001600	0.46549000
6	C	-2.55595300	-1.15468200	0.17974000
7	O	-4.27679800	-2.55062400	-0.60392500
8	O	-0.85687100	0.35222100	0.93504600
9	C	-6.00741100	-0.37287300	-1.11639400
10	H	-6.62790800	0.51501100	-1.29532800
11	O	-6.49116400	-1.49045900	-1.39032600
12	Cl	-2.20793100	2.84888800	0.58192800
13	C	-5.07116600	2.35391700	-0.55761600
14	C	-1.64151500	-2.27186600	0.38588000
15	C	-0.46154200	-2.07631200	0.99109200
16	C	-0.06437500	-0.73394000	1.55484900
17	C	1.40374100	-0.37085400	1.25876300
18	C	-0.34337900	-0.68032100	3.06474700
19	C	1.76975200	-0.37092300	-0.23222800
20	C	3.22974700	0.02024800	-0.60582800
21	C	3.58301000	1.45874700	-0.08215300
22	C	5.03503400	1.85657500	-0.40955700
23	C	6.06493300	0.83291900	0.10553200
24	C	5.68520100	-0.57679800	-0.29163200
25	C	4.25580700	-0.99255300	0.04828800
26	O	6.47921700	-1.33013400	-0.84602200
27	C	4.01761200	-2.47832900	-0.24612200
28	C	3.32688100	-0.05660600	-2.14719100
29	C	2.62730700	2.56942700	-0.55192400
30	H	-5.20268600	-2.47571600	-0.96575700
31	H	-4.56500400	3.27775700	-0.28152800
32	H	-5.34382800	2.42819300	-1.61643600
33	H	-6.00001500	2.29818900	0.02084800
34	H	-1.93574700	-3.25169900	0.02476900
35	H	0.23471800	-2.89632900	1.14672700
36	H	1.58895600	0.61169700	1.70722800
37	H	2.02181300	-1.08887300	1.81230200
38	H	0.23262300	-1.45740400	3.57930400
39	H	-0.05733200	0.29540600	3.47299000
40	H	-1.40578200	-0.85287700	3.26601000
41	H	1.56222100	-1.36265100	-0.65121100
42	H	1.09302900	0.31106500	-0.75875200
43	H	3.51285800	1.41713700	1.01500400
44	H	5.15602200	1.98430800	-1.49301500
45	H	5.25140800	2.83335400	0.03982300

46	H	7.07401700	1.05271800	-0.25688500
47	H	6.08682000	0.86991500	1.20505600
48	H	4.17517900	-0.84378300	1.13649500
49	H	4.07068500	-2.70139600	-1.31606900
50	H	3.04270900	-2.80687500	0.12454600
51	H	4.78190000	-3.08444800	0.25083900
52	H	2.64180100	0.65639000	-2.61790500
53	H	3.05006700	-1.05352300	-2.50603700
54	H	4.33438700	0.15587600	-2.52093700
55	H	2.64878100	2.69462200	-1.64093100
56	H	2.92825000	3.52602900	-0.10780700

Conformer B		Standard Orientation (Ångstroms)		
I	Atom	X	Y	Z
1	C	3.50778800	0.38925600	-1.24586800
2	C	4.05574600	-0.72346600	-0.54497800
3	C	3.53232400	-1.09297700	0.73450900
4	C	2.47977200	-0.34308400	1.25027200
5	C	1.92293600	0.74467600	0.53297100
6	C	2.44602200	1.13147900	-0.71052200
7	O	3.99543400	0.77282100	-2.44086100
8	O	0.92289300	1.43004300	1.11091500
9	C	5.15246700	-1.45628100	-1.13897400
10	H	5.55919400	-2.30946100	-0.58034200
11	O	5.66636700	-1.18014100	-2.24227800
12	Cl	1.77567600	-0.69811400	2.82625200
13	C	4.11489900	-2.26912400	1.48665300
14	C	1.90175800	2.32852500	-1.34154600
15	C	0.80258000	2.91234300	-0.84322900
16	C	0.04203300	2.31763800	0.31836900
17	C	-1.13931200	1.47202500	-0.22101800
18	C	-0.38916800	3.40295900	1.30683700
19	C	-2.03952200	0.83044000	0.84604800
20	C	-3.18140900	-0.09920300	0.33509300
21	C	-4.13043900	0.66617800	-0.65541600
22	C	-5.25053200	-0.23811900	-1.20467900
23	C	-4.71120000	-1.52081800	-1.86615100
24	C	-3.71098700	-2.21971800	-0.97153100
25	C	-2.58047200	-1.34182600	-0.44028100
26	O	-3.80693500	-3.41214900	-0.69920100
27	C	-1.53509400	-2.16754300	0.31823800
28	C	-3.94748000	-0.58472600	1.58759900
29	C	-4.74671000	1.95838200	-0.09091500
30	H	4.74024200	0.15087800	-2.66920900
31	H	3.58585100	-2.44194600	2.42267400
32	H	4.05257400	-3.18903600	0.89513100
33	H	5.17061500	-2.10071400	1.72821200
34	H	2.42205600	2.74068900	-2.19981200
35	H	0.39228000	3.81776300	-1.28299000

36	H	-1.72319100	2.13842100	-0.86818900
37	H	-0.71094800	0.70629600	-0.87953600
38	H	-1.13911900	4.05234300	0.84252300
39	H	-0.82093300	2.96365500	2.21007400
40	H	0.47004000	4.01648700	1.59560900
41	H	-1.41584100	0.26212200	1.54422800
42	H	-2.50714700	1.61846400	1.44645500
43	H	-3.51206700	0.95432100	-1.51877600
44	H	-5.94698200	-0.50626000	-0.39966400
45	H	-5.83710900	0.32164600	-1.94322100
46	H	-5.51649300	-2.21595500	-2.12330700
47	H	-4.18912600	-1.25092100	-2.79633400
48	H	-2.10127200	-0.93370700	-1.34366000
49	H	-1.93984000	-2.60301600	1.23689600
50	H	-0.66379300	-1.56199700	0.58133100
51	H	-1.18739300	-2.99539900	-0.30813900
52	H	-4.39572100	0.25873300	2.12327500
53	H	-3.27143400	-1.08749800	2.28709600
54	H	-4.75112700	-1.28939400	1.34772100
55	H	-5.40524400	1.75878700	0.76256400
56	H	-5.35338500	2.44514400	-0.86406500

Conformer C		Standard Orientation (Ångstroms)		
I	Atom	X	Y	Z
1	C	-3.49845400	-1.18752500	0.52617900
2	C	-4.27547800	-0.70356500	-0.56542000
3	C	-3.98357800	0.57246000	-1.14363600
4	C	-2.92647500	1.30407500	-0.61076100
5	C	-2.14610400	0.80507200	0.46100200
6	C	-2.43642000	-0.43632600	1.04805100
7	O	-3.76188000	-2.37622000	1.10077800
8	O	-1.16212200	1.58396800	0.93963600
9	C	-5.36597600	-1.51306300	-1.06371700
10	H	-5.95279900	-1.11849300	-1.90350300
11	O	-5.68362900	-2.62702300	-0.59929800
12	Cl	-2.49062500	2.89261500	-1.23759400
13	C	-4.80578800	1.09086300	-2.30287800
14	C	-1.66437800	-0.83698200	2.21867200
15	C	-0.57212100	-0.14576500	2.57605300
16	C	-0.05876200	1.00649100	1.74542700
17	C	1.01505900	0.56036900	0.72099800
18	C	0.40260700	2.17213600	2.62106700
19	C	2.26739700	-0.08045700	1.34211000
20	C	3.38781000	-0.53095800	0.35707400
21	C	2.83848900	-1.55747200	-0.69675200
22	C	3.92148600	-1.99268100	-1.70273000
23	C	4.59079200	-0.80072500	-2.41364900
24	C	5.02656200	0.25722200	-1.42354500
25	C	3.95423000	0.71519900	-0.43773800

26	O	6.16496700	0.71446500	-1.42234000
27	C	4.43765100	1.89555100	0.41323700
28	C	4.50775400	-1.15582500	1.22119000
29	C	2.16704600	-2.80396500	-0.09410100
30	H	-4.54226000	-2.76963100	0.62123400
31	H	-4.43398800	2.05257300	-2.65334600
32	H	-4.78069000	0.39619700	-3.14954700
33	H	-5.85483200	1.22546600	-2.01528500
34	H	-2.01356500	-1.68330600	2.80107400
35	H	-0.00534500	-0.40917200	3.46458800
36	H	0.53243500	-0.14678500	0.03635400
37	H	1.28195800	1.44629700	0.13160200
38	H	1.22670200	1.86039800	3.26963300
39	H	0.74481500	3.00640300	1.99984200
40	H	-0.41884000	2.51822000	3.25614300
41	H	2.71394000	0.61271700	2.06310000
42	H	1.96906500	-0.95803300	1.92744200
43	H	2.06981700	-1.02631800	-1.27769300
44	H	4.68739800	-2.59334400	-1.19535800
45	H	3.46978000	-2.64730100	-2.45783700
46	H	5.45199500	-1.11468800	-3.01168000
47	H	3.86178600	-0.33414100	-3.09304000
48	H	3.12784400	1.07030600	-1.07292000
49	H	5.24863000	1.61257300	1.09128000
50	H	3.62297300	2.31661300	1.00894900
51	H	4.81857400	2.69105800	-0.23517800
52	H	4.14196500	-2.03807400	1.75693100
53	H	4.86159900	-0.44492900	1.97530100
54	H	5.37745100	-1.46373100	0.63080000
55	H	2.87315100	-3.40865900	0.48703300
56	H	1.77971700	-3.43919700	-0.89983100
57	H	1.32257300	-2.55377300	0.55600700
